# Identification of specific susceptibility loci for the early-onset colorectal cancer

**DOI:** 10.1186/s13073-023-01163-w

**Published:** 2023-03-03

**Authors:** Haoxue Wang, Yimin Cai, Meng Jin, Chao Qun Huang, Caibo Ning, Siyuan Niu, Linyun Fan, Bin Li, Ming Zhang, Zequn Lu, Xuesi Dong, Zilin Luo, Rong Zhong, Heng Li, Ying Zhu, Xiaoping Miao, Xiaojun Yang, Jiang Chang, Ni Li, Jianbo Tian

**Affiliations:** 1 Department of Epidemiology and Biostatistics, School of Public Health, TaiKang Center for Life and Medical Sciences, Wuhan University, Research Center of Public Health, Renmin Hospital of Wuhan University, Wuhan, 430071 China; 2 Department of Oncology, Tongji Hospital, Tongji Medical College, Huazhong University of Science and Technology, Wuhan, 430030 Hubei China; 3 Department of Gastrointestinal Surgery, Zhongnan Hospital of Wuhan University, Wuhan University, Wuhan, Hubei China; 4 Department of Epidemiology and Biostatistics, School of Public Health, Tongji Medical College, Huazhong University of Science and Technology, Wuhan, China; 5 Office of Cancer Screening, National Cancer Center/National Clinical Research Center for Cancer/Cancer Hospital, Chinese Academy of Medical Sciences and Peking Union Medical College, Beijing, China; 6 Chinese Academy of Medical Sciences Key Laboratory for National Cancer Big Data Analysis and Implement, Chinese Academy of Medical Sciences and Peking Union Medical College, Beijing, China; 7 Department of Urology, Tongji Hospital of Tongji Medical College, Huazhong University of Science and Technology, Wuhan, China; 8 Jiangsu Collaborative Innovation Center for Cancer Personalized Medicine, Nanjing Medical University, Nanjing, China

**Keywords:** GWAS, Early-onset CRC, Genetic variants, PRS, *POLA2*

## Abstract

**Background:**

The incidence of early-onset colorectal cancer (EOCRC; patients < 50 years old) has been rising rapidly, whereas the EOCRC genetic susceptibility remains incompletely investigated. Here, we aimed to systematically identify specific susceptible genetic variants for EOCRC.

**Methods:**

Two parallel GWASs were conducted in 17,789 CRC cases (including 1490 EOCRC cases) and 19,951 healthy controls. A polygenic risk score (PRS) model was built based on identified EOCRC-specific susceptibility variants by using the UK Biobank cohort. We also interpreted the potential biological mechanisms of the prioritized risk variant.

**Results:**

We identified 49 independent susceptibility loci that were significantly associated with the susceptibility to EOCRC and the diagnosed age of CRC (both *P* < 5.0×10^−4^), replicating 3 previous CRC GWAS loci. There are 88 assigned susceptibility genes involved in chromatin assembly and DNA replication pathways, mainly associating with precancerous polyps. Additionally, we assessed the genetic effect of the identified variants by developing a PRS model. Compared to the individuals in the low genetic risk group, the individuals in the high genetic risk group have increased EOCRC risk, and these results were replicated in the UKB cohort with a 1.63-fold risk (95% CI: 1.32–2.02, *P* = 7.67×10^−6^). The addition of the identified EOCRC risk loci significantly increased the prediction accuracy of the PRS model, compared to the PRS model derived from the previous GWAS-identified loci. Mechanistically, we also elucidated that rs12794623 may contribute to the early stage of CRC carcinogenesis via allele-specific regulating the expression of *POLA2*.

**Conclusions:**

These findings will broaden the understanding of the etiology of EOCRC and may facilitate the early screening and individualized prevention.

**Graphical Abstract:**

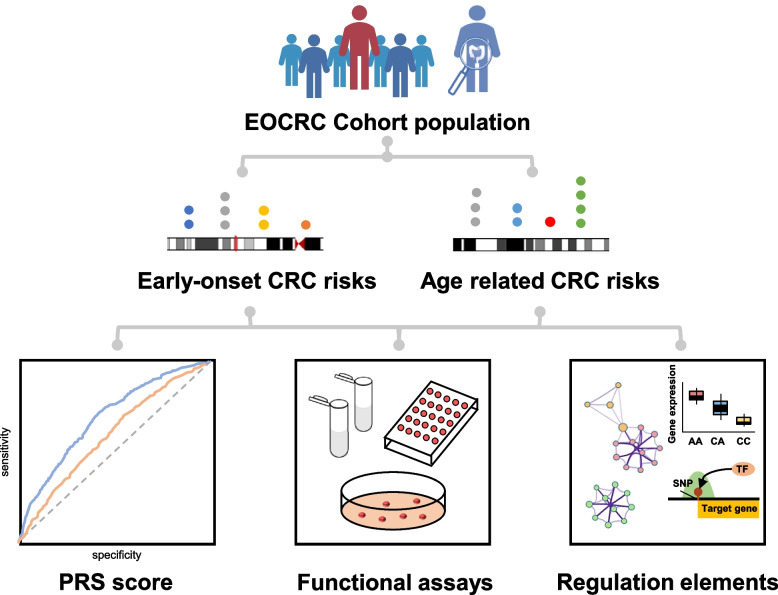

**Supplementary Information:**

The online version contains supplementary material available at 10.1186/s13073-023-01163-w.

## Background

With approximately 1.9 million new colorectal cancer (CRC) cases and 935,000 deaths, CRC is the third most common cancer and the second leading cause of cancer deaths in 2020 [1]. Over the last several decades, CRC mortality has been steadily declining in many countries, mainly attributed to a healthier lifestyle, early detection, and surveillance [2]. However, the incidence of newly diagnosed early-onset CRC cases (EOCRC; diagnosed CRC <50 years old) has increased by about 2% annually, accounting for 2% to 8% of all CRC cases [3, 4]. As estimated about 15% of all CRC cases will be diagnosed in patients aged younger than 50 years by 2030 [5]. For adults younger than 50 years old, routine CRC screening is not the standard, such as the invasive colonoscopy [6]. Precision cancer screening at an earlier age will benefit the risk discrimination for the high-risk individuals. Therefore, efforts have now focused on investigating the risk factors of EOCRC in order to elucidate more targeted screening approaches and reduced the disease burden.

EOCRC is genetically, pathologically, and molecularly heterogeneous compared with late-onset CRC [7], since EOCRC tends to show higher pathologic grade and an upward tendency of recurrence and metastasis [8]. EOCRC can be classified into three subgroups: familiar, hereditary, and sporadic EOCRC. Current genetic studies of EOCRC mainly focused on rare monogenic diseases and hereditary EOCRC [9]. First, as the typical familiar EOCRC, familial adenomatous polyposis (FAP) is characterized by numerous colorectal adenomas, and the individuals with APC germline pathogenic mutations usually developed CRC before 40 years old [2]. Moreover, Lynch syndrome is also known as hereditary nonpolyposis colorectal cancer (HNPCC) syndrome. Germline mutations encoding the DNA mismatch repair system (*MLH1*, *MSH2*, *MSH6*, and *PMS2*) might lead to the increase in the cellular mutational burden and cancer development [10]. For patients with familiar and hereditary EOCRC, routine molecular screening and prophylactic colectomy should be suggested respectively to assess the frequency of germline mutations in patients and their family members [11, 12]. For sporadic EOCRC, both environmental factors and genetic susceptibility contribute to its occurrence [13], among which the associated germline genetic variants remain unclear [14].

Genome-wide association studies (GWASs) identify causal variants in genome to facilitate the evaluation of human complex traits and diseases [15]. More than 100 CRC risk loci have been identified by GWASs [16]. A recent study revealed that polygenic risk score (PRS) based on 95 common CRC risk variants was also strongly associated with EOCRC risk [9]. These results indicated that it is crucial to conduct a GWAS to systematically investigate the genetic susceptibility specific to sporadic EOCRC. Hence, our aim was to systematically elucidate the causal genetic variants and understand sporadic EOCRC etiology, which will promote targeted early screening and treatment strategies for the high-risk individuals.

We evaluated associations between germline variants and EOCRC risk in 2 complementary GWASs in a large cohort from European ancestry and identified 49 independent genetic loci significantly associated with EOCRC risk. Furthermore, we developed a PRS model to assess the genetic effect of these identified variants and tested its prediction value in the UK Biobank cohort. Moreover, we investigated the biological plausibility of the top significant EOCRC-specific susceptibility loci. rs12794623 allele-specific facilitated the expression of DNA repair genes *POLA2* and enhanced CRC cell proliferation. These findings expanded our insights into the underlying risk of EOCRC and may help to determine surveillance strategies of EOCRC.

## Methods

### Cohort descriptions

#### GECCO cohort

In the GWAS stage, all participants were pooled from a large-scale cohort from the Genetics and Epidemiology of CRC Consortium and Colon Cancer Family Registry (GECCO) with three datasets (phs001078.v1.p1, phs001315.v1.p1, and phs001415.v1.p1) [16]. The diagnosis of cases was following the medical records, pathologic reports, cancer registries, or death certificates. Demographic characteristics were collected from a previously published study [16]. Demographic characteristics were demonstrated in Additional file 2: Tables S1 and S2. Reference age is defined as age of diagnosis of first primary CRC for CRC cases, or refers to age at sample selection in healthy controls. A total of 17,863 CRC cases, including 1490 EOCRC cases (241 EOCRC cases under 40 years old), and 20,037 controls of European ancestry were enrolled for the next analysis.

#### UK Biobank cohort

We also screened participants in the UK Biobank cohort for validation. The data regarding this project were from Application No. 51539. All participants signed an informed consent form, and the UKBB study was ethically approved by the North West Multicenter Research Ethics Committee. Demographic characteristics were demonstrated in Additional file 2: Tables S3 and S4 [17]. CRC cases were defined as subjects with primary invasive CRC diagnosed (1020–1023), or CRC deaths according to ICD9 (1530–1534, 1536–1541) or ICD10 (C180, C182–C189, C19, C20) codes. For each case, we selected 4 eligible controls from subjects without invasive CRC by nearest neighbor matching in R package MatchIt, with enrollment age, enrollment year, ethnicity, and sex as matching criteria. A total of 6,296 CRC cases and 25,184 matched controls were enrolled. After exclusion of the participants without the genotype data, we finally included 723 EOCRC cases (185 EOCRC cases under 40 years old) and 24,427 matched controls. We also collected the demographic, social and behavioral factors including information on ethnicity, drinking frequency, smoking status, and family history of bowel cancer. Ethnicity was defined as White, Mixed, Asian or Asian British, Black or Black British, and other ethnic groups (defined by the original UK Biobank data) via the self-reported questionnaire. CRC family history was derived from the bowel cancer history of the father, mother, and siblings. Smoking status was categorized as “current” or “former” or “never”. For the alcohol intake frequency, we divided participants into heavy alcohol consumption (>3 times/week), moderate consumption as consuming fewer than these amounts, and never.

### Genotype data collection, imputation, and quality control

GECCO genotype data were obtained from the database of Genotypes and Phenotypes (dbGaP) under accession numbers phs001078.v1.p1, phs001315.v1.p1, and phs001415.v1.p1 [16]. Imputation was conducted using Michigan Imputation Server [18], with Haplotype Reference Consortium r1.1.2016 (HRC) as a reference panel [19]. We merged all the batches into a single set after imputation. Several criteria were applied to filter out redundant variants using plink1.9 [20]: (1) SNPs with imputation quality < 0.4; (2) SNPs with minor allele frequency < 1%; (3) SNPs deviating from the Hardy-Weinberg equilibrium (*P* < 10^−6^); (4) SNPs with missing call frequencies > 0.02 and SNPs located in the sex chromosome. Additionally, we removed samples without age information. A total of 37,740 individuals with 2,446,560 SNPs were finally remained. To remove the effect of population stratification and structure, we performed PCA using SmartPCA in EIGENSOFT v6.1.4 and selected the top ten PCs for subsequent analyses. No significant population stratifications were observed for cases or controls in these two stages (Additional file 1: Fig. S1).

### Association analysis

We adopted two complementary approaches of regression analyses to identify EOCRC-specific susceptibility loci by both EOCRC risks association and CRC onset age association. In the first approach, SNPs were tested for the EOCRC-specific association in 1490 early-onset cases and all healthy control using logistic regression with the adjustment of sex, recruitment center, and 10 principal components. In the second approach, to account for residual confounding of CRC onset age, we tested the association for each variant by using a linear regression model with the independent variable being diagnosed age in all 17,789 CRC cases. Sex, recruitment center, and 10 principal components were adjusted in this approach. Furthermore, to identify the specific susceptibility variants of early-onset CRC (under 40 years old), we also conducted the analysis by using 40 years old as the age cut-off of early-onset CRC. The controls were selected by R package MatchIt with matching sex and recruitment center, and the matching ratio was following the incidence of early-onset CRC risk in the GECCO cohort. A total of 241 EOCRC cases under 40 years old and 3374 matching controls were enrolled. We performed the association for variants by using logistic regression with the adjustment of sex, recruitment center, and 10 principal components.

### Polygenic risk score model building

PRS is analyzed by effect sizes estimated from a genome-wide association study, representing a quantitative metric of inherited risk. During PRS calculation, these factors should be considered, including the weights to use for each SNP, the amount of including SNPs, and correlations thresholding between the SNPs (linkage disequilibrium, LD). We calculated the PRS score in clumping and thresholding by preferentially selecting a subset of risk-associated SNPs.

$${\textrm{PRS}}_{P_T,J}=\sum_{i=1}^m{\beta}_i\ {G}_{i,j}$$

PRSice can provide the most precise threshold and best-fit PRS of the phenotype [21], through performing clumping to remove ambiguous SNPs and SNPs in LD (*r*^2^ ≥ 0.1 within 250 kilobases) with others. Effect sizes for all SNPs were derived from the association result of EOCRC in the GECCO cohort. To develop the weighted PRS_CRC_, we overlapped 87 SNPs derived from the literature and 40 SNPs previously identified within the GECCO cohort which reached genome-wide significance (*P* < 5×10^−8^) [16]. After filtering the variants LD *r*^2^ > 0.6, 86 CRC GWAS SNPs remained (Additional file 2: Table S5), and the weighted PRS was developed using previously published log-odds ratios from GWAS publications.

### Prediction model and external validation

We built risk-prediction models using logistic regression algorithms in 1490 cases and 19,951 controls, with models including sex and the weighted PRS. Furthermore, we examined the optimal values of the tuning parameters using 10-fold cross-validation from R package caret. We further evaluated the prediction model in the UK Biobank cohort (723 EOCRC cases and 24,427 matched controls) with adjustment of sex, the weighted PRS, and several sociodemographic exposure factors (ethnicity, drinking frequency, smoking status, and family history of bowel cancer) additionally. AUC was calculated to evaluate the discriminatory accuracy of the risk prediction model by R package pROC.

### Functional annotation for variants

We next performed a functional annotation for the risk SNPs by using multiple bioinformatic tools and databases, including the HaploReg database (https://pubs.broadinstitute.org/mammals/haploreg/haploreg.php), Roadmap Epigenomics Consortium (https://www.ncbi.nlm.nih.gov/geo/roadmap/epigenomics/), ENCODE database (http://compbio.mit.edu/encode-motifs/), RegulomeDB database (https://www.regulomedb.org/regulome-search), CADD database (https://cadd.gs.washington.edu/score), 3DSNP (https://omic.tech/3dsnpv2/), which integrated multiple histone modification chromatin immunoprecipitation (ChIP)-seq peaks, transcription factor (TF) ChIP-seq peaks. To be specific, (1) regulatory chromatin histone ChIP-Seq were characterized by using the data from the Roadmap Epigenomics Consortium (Epigenome ID as E106 Sigmoid Colon; E075 Colonic Mucosa; E101 and E102 Rectal Mucosa); (2) regulatory motifs were predicted by the ENCODE TF ChIP-seq datasets following the bound sequences; and (3) prioritizing these variants with the scores of regulatory elements by using RegulomeDB, CADD, and 3DSNP prediction databases. Finally, the total functional score was the mean value of respective Z-scores of the RegulomeDB score, CADD score, and 3DSNP score.

### Cell lines

HCT116 (RRID:CVCL_0291) and SW480 (RRID:CVCL_0546) cell lines were obtained from the China Center for Type Culture Collection (Wuhan, China). Cells were cultured in Dulbecco’s modified Eagle’s medium (DMEM; Gibco, USA) supplemented with 10% fetal bovine serum (FBS; Gibco, USA) and 1% antibiotics (100 U/ml penicillin and 0.1 mg/ml streptomycin) at 37°C in a humidified atmosphere of 5% CO_2_. All cell lines that we used in this study were tested and authenticated by DNA sequencing using the AmpF/STR method (Applied Biosystems, USA) and tested for the absence of mycoplasma contamination (MycoAlert, USA) within the last 3 years.

### Dual-luciferase reporter assay

The plasmids were conducted with a 1-kb DNA sequence around rs12794623 (allele: C/A) and cloned into PGL3-basic (Promega, Madison, USA). HCT116 and SW480 cells were plated in 96-well plates 24 h before transfection (1×10^4^ cells per well). Lipofectamine 3000 (Invitrogen, Waltham, USA) was used to transfect the reporter plasmids and a pRL-SV40 luciferase plasmid (Promega, Madison, USA) into cells. After 48-h incubation with 5% CO_2_ at 37°C, cells were lysed by 1×passive lysis buffer (Promega, Madison, USA). Luciferase activity was detected by the Dual-Luciferase Reporter Assay System (Promega, Madison, USA). Data were independently conducted in triplicate. Comparisons were conducted by unpaired *t*-test.

### Quantitative real-time (qRT)-PCR

Total RNA of CRC patients’ tissues and CRC cell lines were extracted using TRIzol reagent (Invitrogen, USA). A total of 154 CRC patients were recruited from Tongji Hospital of Huazhong University of Science and Technology (HUST) and Zhongnan Hospital of Wuhan University, Wuhan, China. One hundred fifty-four matched colorectum tumor and normal colon mucosa biopsy specimens were obtained endoscopically. This study was approved by the Biomedical Ethics Committee of Wuhan University, and all participants provided written informed consent prior to data collection. And reverse transcription was performed by the PrimeScript™ RT Master Mix (TaKaRa Bio, Tokyo, Japan). The relative expression levels of *POLA2* and GR were detected by qRT-PCR using a SYBR™ Green Master Mix (Applied Biosystems, California, USA) on 7900HT Fast RT PCR System (Applied Biosystems, Foster City, CA). Using the 2^−ΔΔCt^ method, the expression levels of *POLA2* and *GR* were normalized to that of *GAPDH*, as an endogenous control (Additional file 1: Fig. S2). All primers used are listed in Additional file 2: Table S6.

### Electrophoretic mobility shift assays (EMSA)

The double-stranded DNA oligonucleotides centered on rs12794623 alleles were synthesized with biotin-labeled 3′ end (TaKaRa Bio, Tokyo, Japan) (Additional file 2: Table S6). Nuclear extracts of HCT116 and SW480 cells were extracted using a Nuclear and Cytoplasmic Protein Extraction Kit (Beyotime, Shanghai, China). The binding activity of the protein was detected by using an EMSA/Gel-Shift Kit (Beyotime, Shanghai, China). For the competitive binding experiments, specific binding was performed with a 100-fold excess of unlabeled, identical oligonucleotides. After incubated for 20 min, those reaction mixtures were separated on a native 8% PAGE gel and results were detected by SuperSignal West Femto Trial Kit (Thermo, Rockford, USA).

### Cell proliferation determination

*POLA2* pcDNA3.1 plasmid (RIBOBIO, Guangzhou, China) was transfected in HCT116 and SW480 cells by using Lipofectamine 3000 (Invitrogen, Waltham, USA). The cells were further seeded in 96-well plates after 24 h of culture, and each well contained 2000 cells. Cell viability was measured with CCK-8 assays (Dojindo, Japan) following the manufacturer’s instruction after 24 h, 48 h, 72 h, and 96 h. The absorbance at 450 nm was recorded with six technical replicates and each experiment was repeated in triplicate.

### Colony formation assay

After transfected *POLA2* pcDNA3.1 plasmid, HCT116 or SW480 cells were cultured in 6-well plates (1000 cells per well. We changed the culture medium once a week with fresh DMEM with 10% FBS medium. After incubation for 2–3 weeks, colonies were washed twice with PBS solution and fixed with 100% methanol. Then crystal violet solution (Solarbio, Beijing, China) was used to stain colonies at room temperature for 30 min. Deionized water was used to clean the colonies, then the colonies were photographed. Each experiment was repeated three times.

### Statistical analyses

Differences in demographic characteristics between cases and controls were assessed by Student’s *t*-test or Pearson *χ*^2^ test. Quantile-quantile (*Q*-*Q*) plots were assessed to determine whether the distribution of the *P value* was consistent with null distribution (except for the extreme tail). The genomic-inflation factor (λ) in distinct approaches were presented in Additional file 1: Fig. S3. Inclusion criteria of candidate SNPs were (1) attained genome-wide significance (*P* < 5.0×10^−4^) in both EOCRC risks association and CRC onset age association and (2) with odds ratio (OR) > 1 in EOCRC risks associations or beta < 0 in CRC onset age-related association. ORs and 95% confidence intervals (CI) were also estimated comparing quartiles of PRS. The data of tumor mutational burden (TMB) and gene expression data were obtained from COAD and READ tissues in the TCGA database (https://portal.gdc.cancer.gov). TMB per megabase is calculated by dividing the total number of mutations by the size of the coding region of the target. Samples were divided into two groups based on median TMB. All *P* < 0.05 calculated by unpaired two-sided Student’s *t*-test were considered significant. All statistical analyses were performed by R (4.0.3) or PLINK (1.9) software. GraphPad Prism v6.0 Software was adopted to create graphs.

## Results

### The study overview and characteristics of the participants

**Fig. 1:**
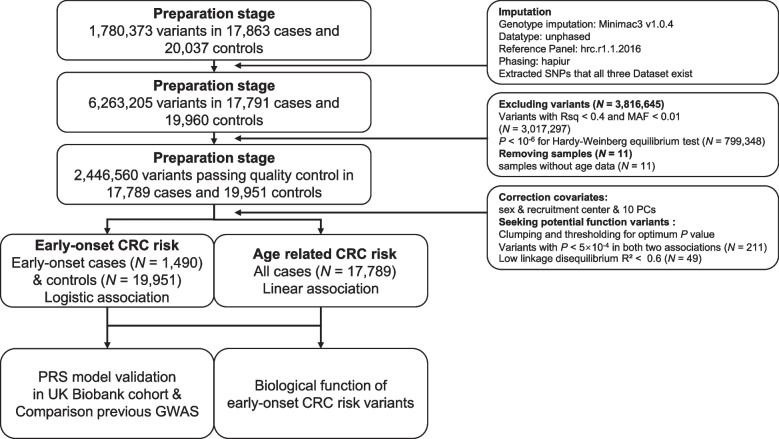
Summary of the study design and workflow. We systematically identified the genetic susceptibility of EOCRC and assessed those genetic effects by developing a PRS derived from those genetic variants in diverse populations. Moreover, by functional analysis, we investigated one of the biological plausibility of the identified variants in association with EOCRC

We conducted GWAS in a large-scale population to identify genetic variants significantly associated with EOCRC risk. The workflow was graphed in Fig. 1. Following imputation and quality control, we obtained 2,446,560 SNPs in 17,789 CRC cases and 19.951 controls. There were 1490 EOCRC cases (under 50 years old) accounting for approximately 8% of all CRC cases and about 4% of all subjects, and 241 EOCRC cases (under 40 years old) occupying around 1% of all CRC cases and about 0.6% of all subjects. Mean age at diagnosis of EOCRC cases was 44.1 years, while for late-onset CRC cases, it was 65.6 years. Men and women were nearly equivalently represented across cases and controls (Additional file 2: Tables S1 and S2).

### Identification of EOCRC-specific susceptibility loci

**Fig. 2:**
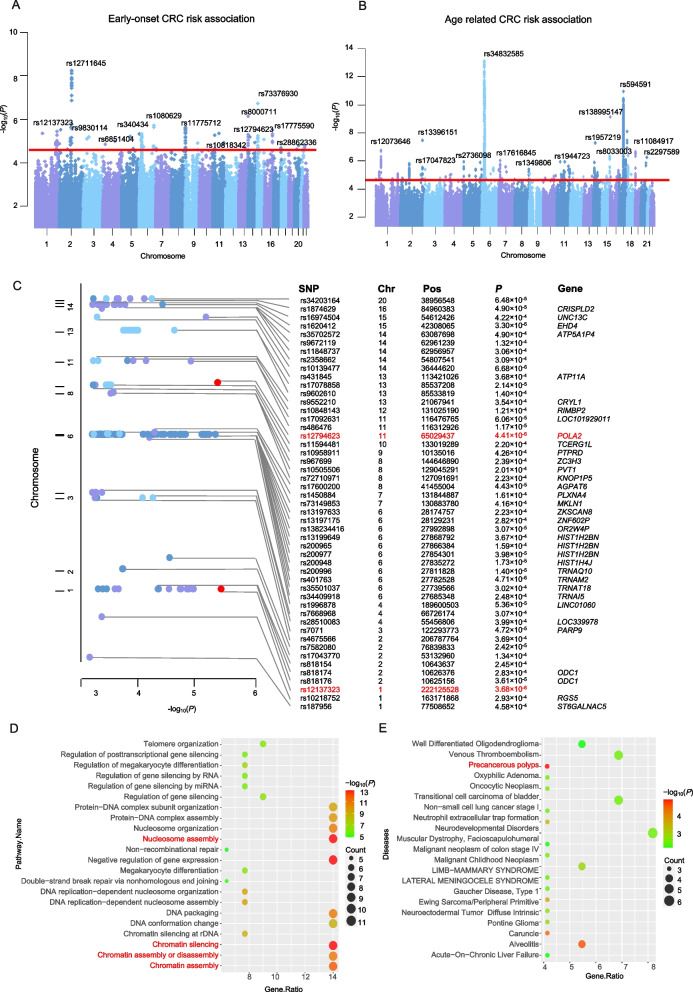
Manhattan plots for associations between genetic variants and EOCRC risk. **A** The logistic regression analysis of 1490 EOCRC cases and 19,951 controls. **B** The linear regression analysis with the independent variable being diagnosed age of 17,789 CRC cases. *P* values are two-sided, calculated by an additive model, and adjusted for sex, recruitment center, and the 10 principal components. The red line indicates the genome-wide significance threshold. The associations (–log_10_(*P*) values, *y*-axis) are plotted against genomic position (*x*-axis by chromosome and chromosomal position of NCBI build 37). **C** Manhattan plot shows the annotation of all 49 genetic risk variants independently (LD *r*^2^ < 0.6) associated with EOCRC risk in the GECCO cohorts. Gene indicates the mapped genes of variants. The red dots indicate the top two EOCRC risk variants: rs12137323 and rs12794623. The *x*-axis represents the –log_10_(*P*) values of the SNPs, and the y-axis represents the chromosomal positions. **D** Pathway enrichment analysis of target genes revealed that the majority are involved in several oncogenic pathways, such as chromatin assembly or disassembly and DNA replication (chromatin silencing and nucleosome assembly) (marked in red). **E** Disease association analysis of target genes by DisGeNET databases, and target genes are most significantly contributed to precancerous polyps (marked in red)

We performed two complementary regression analyses to identify EOCRC-specific susceptibility loci as described in methods. In the EOCRC risks association analysis, we identified 2118 loci were significantly associated with EOCRC susceptibility with *P* < 5.0×10^−4^ (Fig. 2A). And 5071 variants were significantly associated with CRC onset age in the linear association analysis (Fig. 2B). After combining these two analysis results, we identified 211 variants (49 independent signals with *r*^2^ < 0.6) were significantly associated with EOCRC risk (Table 1, Additional file 1: Fig. S4 and Additional file 2: Table S7).

**Table 1:** Results for 49 identified EOCRC risk loci in the GECCO cohort (LD *r*^2^ < 0.6)

SNP	Chr	Pos.	GWAS loci ±1 Mb region	Effect allele	Early-onset CRC risk association						Age related CRC risk association	
					Rsq	Type	EAF		OR (95% CI)	*P *^‡^	Beta (-95% CI)	*P *^#^
							Cases	Controls				
rs12137323	1	222125528	Yes	A	-	Genotyped	0.19	0.16	1.25 (1.15–1.34)	3.68×10^−6^	−0.60 (0.86-0.32)	1.42×10^−5^
**rs12794623**	**11**	**65029437**		**A**	**0.46**	**Imputed**	**0.13**	**0.11**	**1.33 (1.21–1.45)**	**4.41×10**^**−6**^	**−0.72 (1.07-0.37)**	**4.76×10**^**−5**^
rs401763	6	27782528		C	-	Genotyped	0.11	0.10	1.44 (1.28–1.60)	4.71×10^−6^	−1.58 (2.03-1.13)	7.85×10^−12^
rs10139477	14	36444620		G	0.88	Imputed	0.28	0.24	1.22 (1.13–1.31)	6.68×10^−6^	−0.45 (0.70-0.19)	4.89×10^−4^
rs486476	11	116312926		G	0.83	Imputed	0.28	0.24	1.21 (1.13–1.30)	1.17×10^−5^	−0.52 (0.77-0.27)	4.38×10^−5^
rs200996	6	27811828		A	-	Genotyped	0.10	0.10	1.42 (1.26–1.57)	1.40×10^−5^	−1.54 (1.99-1.08)	2.89×10^−11^
rs200948	6	27835272		C	-	Genotyped	0.10	0.10	1.41 (1.25–1.57)	1.73×10^−5^	−1.53 (1.98-1.07)	4.11×10^−11^
rs17078858	13	85537208		T	0.75	Imputed	0.25	0.21	1.21 (1.12–1.30)	2.14×10^−5^	−0.48 (0.73-0.22)	2.09×10^−4^
rs7582080	2	76839833		A	-	Genotyped	0.28	0.24	1.20 (1.11–1.28)	2.42×10^−5^	−0.45 (0.69-0.20)	3.17×10^−4^
rs138234416	6	27992898		A	0.93	Imputed	0.09	0.08	1.50 (1.31–1.70)	3.07×10^−5^	−2.01 (2.55-1.46)	4.06×10^−13^
rs1620412	15	42308065		G	0.60	Imputed	0.27	0.23	1.20 (1.11–1.28)	3.30×10^−5^	−0.51 (0.75-0.26)	5.31×10^−5^
rs818176	2	10625156		C	0.43	Imputed	0.17	0.17	1.21 (1.12–1.29)	3.61×10^−5^	−0.47 (0.72-0.21)	3.57×10^−4^
rs200977	6	27854301		C	0.98	Imputed	0.10	0.10	1.40 (1.24–1.56)	3.98×10^−5^	−1.54 (2.00-1.07)	9.14×10^−11^
rs17600200	8	41455004		G	-	Genotyped	0.05	0.04	1.38 (1.22–1.53)	4.43×10^−5^	−1.05 (1.54-0.54)	4.52×10^−5^
rs7071	3	122293773		T	0.45	Imputed	0.16	0.12	1.21 (1.12–1.30)	4.72×10^−5^	−0.57 (0.84-0.28)	9.89×10^−5^
rs1874629	16	84960383		A	0.83	Imputed	0.40	0.34	1.17 (1. 10^−^1.25)	4.90×10^−5^	−0.49 (0.70-0.26)	1.41×10^−5^
rs1996878	4	189600503		A	0.52	Imputed	0.03	0.03	1.41 (1.24–1.58)	5.36×10^−5^	−1.02 (1.55-0.47)	2.44×10^−4^
rs17092631	11	116476765		C	-	Genotyped	0.10	0.07	1.32 (1.19–1.46)	6.06×10^−5^	−0.98 (1.40-0.55)	6.43×10^−6^
rs34203164	20	38956548		T	0.84	Imputed	0.09	0.08	1.29 (1.17–1.42)	6.48×10^−5^	−0.69 (1.06-0.30)	3.64×10^−4^
rs10848143	12	131025190		G	0.61	Imputed	0.21	0.19	1.19 (1.10–1.27)	1.21×10^−4^	−0.55 (0.79-0.29)	2.13×10^−5^
rs9672119	14	62961239		T	0.91	Imputed	0.09	0.09	1.27 (1.15–1.39)	1.32×10^−4^	−0.84 (1.21-0.47)	9.26×10^−6^
rs17043770	2	53132960		G	-	Genotyped	0.09	0.07	1.32 (1.18–1.46)	1.34×10^−4^	−0.87 (1.29-0.44)	6.44×10^−5^
rs9602610	13	85533819		G	0.74	Imputed	0.23	0.20	1.19 (1.10–1.28)	1.40×10^−4^	−0.47 (0.72-0.20)	4.08×10^−4^
rs200965	6	27866384		A	0.97	Imputed	0.15	0.16	1.26 (1.14–1.38)	1.59×10^−4^	−1.12 (1.47-0.77)	2.38×10^−10^
rs1450884	7	131844887		C	0.83	Imputed	0.31	0.28	1.18 (1.09–1.26)	1.61×10^−4^	−0.49 (0.73-0.24)	7.33×10^−5^
rs10505506	8	129045291	Yes	G	-	Genotyped	0.47	0.44	1.16 (1.08–1.23)	2.01×10^−4^	−0.38 (0.59-0.16)	4.52×10^−4^
rs11594481	10	133019289		G	0.53	Imputed	0.08	0.07	1.28 (1.15–1.41)	2.20×10^−4^	−0.73 (1.11-0.33)	2.76×10^−4^
rs13197633	6	28174757		A	0.85	Imputed	0.08	0.07	1.48 (1.27–1.69)	2.23×10^−4^	−2.23 (2.80-1.64)	5.86×10^−14^
rs72710971	8	127091691		T	0.44	Imputed	0.03	0.02	1.48 (1.27–1.69)	2.23×10^−4^	−1.21 (1.87-0.54)	3.51×10^−4^
rs967699	8	144646890		G	0.48	Imputed	0.30	0.27	1.18 (1.09–1.27)	2.39×10^−4^	−0.54 (0.77-0.29)	1.44×10^−5^
rs818154	2	10643637		A	0.86	Imputed	0.41	0.40	1.15 (1.08–1.23)	2.45×10^−4^	−0.44 (0.65-0.22)	4.84×10^−5^
rs34409918	6	27685348		G	0.58	Imputed	0.08	0.07	1.46 (1.25–1.66)	2.48×10^−4^	−1.98 (2.53-1.42)	3.59×10^−12^
rs13197175	6	28129231		T	-	Genotyped	0.08	0.07	1.47 (1.26–1.68)	2.82×10^−4^	−2.23 (2.80-1.64)	5.35×10^−14^
rs818174	2	10626376		T	0.54	Imputed	0.38	0.35	1.15 (1.08–1.23)	2.83×10^−4^	−0.41 (0.62-0.19)	2.36×10^−4^
rs10218752	1	163171868		C	-	Genotyped	0.20	0.19	1.18 (1.09–1.27)	2.93×10^−4^	−0.48 (0.73-0.21)	3.59×10^−4^
rs35501037	6	27739566		A	0.85	Imputed	0.08	0.08	1.44 (1.24–1.64)	3.02×10^−4^	−1.98 (2.53-1.42)	2.17×10^−12^
rs11848737	14	62956957		C	0.91	Imputed	0.13	0.12	1.22 (1.11–1.33)	3.06×10^−4^	−0.63 (0.95-0.31)	1.15×10^−4^
rs7668968	4	66726174		G	0.59	Imputed	0.42	0.40	1.15 (1.07–1.22)	3.07×10^−4^	−0.44 (0.64-0.22)	5.64×10^−5^
rs2358662	14	54807541	Yes	C	-	Genotyped	0.25	0.24	1.16 (1.08–1.25)	3.09×10^−4^	−0.46 (0.70-0.22)	1.35×10^−4^
rs9552210	13	21067941		G	0.50	Imputed	0.49	0.46	1.16 (1.08–1.24)	3.54×10^−4^	−0.40 (0.62-0.17)	3.81×10^−4^
rs13199649	6	27868792		T	-	Genotyped	0.08	0.07	1.46 (1.25–1.67)	3.67×10^−4^	−2.14 (2.71-1.56)	3.87×10^−13^
rs431845	13	113421026		C	0.41	Imputed	0.06	0.06	1.31 (1.16–1.46)	3.68×10^−4^	−0.83 (1.29-0.36)	4.33×10^−4^
rs4675566	2	206787764		C	0.98	Imputed	0.26	0.24	1.17 (1.08–1.26)	3.69×10^−4^	−0.52 (0.78-0.26)	5.69×10^−5^
rs28510083	4	55456806		T	0.96	Imputed	0.22	0.20	1.17 (1.09–1.26)	3.99×10^−4^	−0.53 (0.79-0.27)	4.37×10^−5^
rs73149853	7	130883780		A	0.89	Imputed	0.17	0.13	1.22 (1.11–1.33)	4.16×10^−4^	−0.61 (0.94-0.28)	2.85×10^−4^
rs16974504	15	54612426		G	-	Genotyped	0.15	0.11	1.22 (1.11–1.34)	4.22×10^−4^	−0.66 (1.00-0.32)	1.32×10^−4^
rs10958911	9	10135016		A	0.47	Imputed	0.13	0.11	1.21 (1.11–1.32)	4.26×10^−4^	−0.59 (0.91-0.26)	3.88×10^−4^
rs187956	1	77508652		G	0.82	Imputed	0.35	0.32	1.15 (1.07–1.23)	4.58×10^−4^	−0.45 (0.67-0.22)	1.05×10^−4^
rs35702572	14	63087698		C	0.52	Imputed	0.28	0.24	1.16 (1.08–1.25)	4.90×10^−4^	−0.48 (0.72-0.23)	1.16×10^−4^

Rsq values that reflect imputation quality are obtained from the output of Michigan Imputation Server

*Chr* chromosome, *Pos* position, *EAF* effect allele frequency, *OR* odds ratio, *CI* confidence interval, *P* *P* value

^‡^*P* values were calculated by unconditional logistic regression model after adjusting for sex, recruitment center and 10 principal components

^#^*P* values were calculated by linear regression model after adjusting for sex, recruitment center and 10 principal components

We also replicated three reported CRC GWAS loci (Additional file 2: Table S5), namely, 1q41 (rs12137323, *P* = 3.68×10^−6^, OR = 1.25, 95% CI =1.15–1.34), 8q24.21 (rs10505506, *P* = 2.01×10^−4^, OR = 1.16, 95% CI =1.08–1.23) and 14q23.1 (rs2358662, *P* = 3.09×10−^4^, OR = 1.16, 95% CI =1.08–1.25). As the strongest signal in the 1q41 region, rs12137323 (sorted by *P*-value in the first analysis) is in strong LD (*r*^2^ = 0.81) with previous GWAS identified tagSNP rs6687758 in the East Asian [22, 23]. The tagSNP rs6687758 locates 125kb upstream of *DUSP10*, a dual-specificity phosphatase inactivating p38 and SAPK/JNK pathways [24]. Additionally, inhibition of *DUSP10* was reported to be associated with gut inflammation, which might lead to the early development of intestinal cancer [25].

For EOCRC under 40 years old, we further identified the 1,519 susceptibility variants with *P* < 5.0×10^−4^ (Additional file 1: Fig. S5). After combining the age association analysis results, we found 84 variants (16 independent signals with *r*^2^ < 0.6) were significantly associated with EOCRC risk under 40 years old (Additional file 2: Tables S8 and S9). There were several risk variants associated with EOCRC risks in both two age cut-off groups (Additional file 2: Table S10).

### The identified EOCRC risk variants point to susceptibility genes

We then mapped those 211 risk variants to their related genes by VarioWatch [26], generating 88 genes potentially relative with EOCRC risk, including 57 protein-coding genes and 31 non-coding genes (Fig. 2C and Additional file 2: Table S11). For instance, the hypermethylation of transcription elongation regulator 1-like (TCERG1L) is observed in precancerous colon polyps [27] and has been regarded as a risk marker of CRC in patients with ulcerative colitis [28]. Then we performed pathway and process enrichment analysis, noticing pathways involved in chromatin assembly and DNA replication (chromatin silencing and nucleosome assembly), including cell cycle-dependent histone H4 family genes (Fig. 2D). Furthermore, the gene-disease association analysis was conducted to explore the associated traits by DisGeNET databases [29]. We observed that those genes significantly contributed to precancerous polyps which were known as the precursors of CRC (Fig. 2E) [30]. Collectively, it suggested that EOCRC-specific risk loci may alter gene expression and dysregulate signaling pathways involved in EOCRC progression.

### EOCRC-specific susceptibility loci can promote predictive power of EOCRC risk

**Fig. 3:**
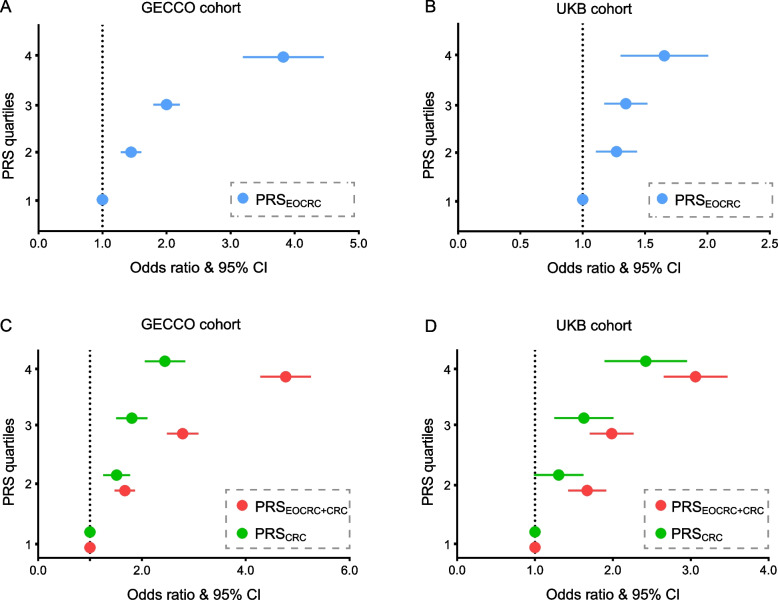
Risk estimates for EOCRC associated with the PRS deriving from distinct SNPs. **A** The PRS was generated by calculating the effect sizes of EOCRC-specific risk loci in 1490 EOCRC cases (< 50 years) and 19,951 healthy controls in the GECCO cohort and **B** in 723 EOCRC cases (< 50 years) and 24,427 healthy controls in the UKB cohort. **C** Two PRS models were derived from 86 previously GWAS-identified CRC risk SNPs and those 86 SNPs combined with 49 EOCRC-specific SNPs in 1490 EOCRC cases (< 50 years) and 19,951 healthy controls in the GECCO cohort, respectively. **D** in 723 EOCRC cases (< 50 years) and 24,427 healthy controls in the UKB cohort. Models were adjusted for sex and PRS quartiles. The PRS scores were modeled as a continuous variable per 1 standard deviation (SD), transformed to the standard normal distribution. ORs and 95% confidence intervals were estimated by comparing quartiles of PRS. The dashed line indicated the odds ratio = 1 as reference

To quantify the prediction value of identified EOCRC-specific susceptibility loci, we next generated PRS_EOCRC_ by calculating the effect sizes of 49 identified tagSNPs with unconditional logistic regression. We found that early-onset CRC cases showed marked skewing higher PRS quartiles compared with controls in the GECCO cohort (Additional file 2: Table S12). The highest PRS_EOCRC_ quartile had a 3.8-fold risk than the lowest (OR = 3.79; 95% CI: 3.21–4.47) (Fig. 3A). Interestingly, the associations were successfully replicated in the UKB cohort, presenting a 1.6-fold risk for individuals with highest PRS_EOCRC_ quartile (OR: 1.63; 95% CI: 1.32–2.02) (Fig. 3B and Additional file 2: Table S13). We then tested whether these 49 EOCRC-specific risk variants could improve EOCRC risk prediction performance by comparing PRS_CRC_ derived from 86 previously identified CRC risk SNPs and PRS_EOCRC+CRC_ derived from combining those SNPs. The latter significantly increased the ability to predict EOCRC risk (Fig. 3C, D and Additional file 2: Tables S12 and S13). To further quantify the prediction value of identified the EOCRC (under 40 years old) susceptibility loci, we examined PRS scores derived from 16 identified specific EOCRC (under 40 years old) variants in 241 EOCRC cases (under 40 years old) and 3374 healthy controls in the GECCO cohort, and found similar results (Additional file 1: Fig. S6). Collectively, the predictive power of PRS increased after including the genetic effects of EOCRC-specific susceptibility loci.

**Fig. 4:**
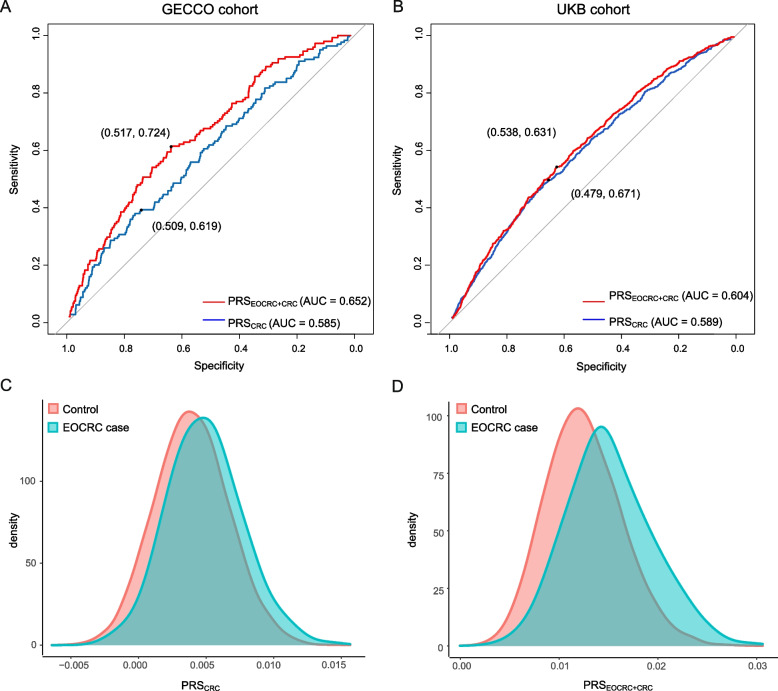
The ROC curve for the PRS predicted models. Two ROC curves for the distinct PRS logistic models weighted the effects of 86 previous GWAS-identified susceptibility loci and GWAS loci combined with EOCRC-specific risk variants, respectively, **A** in the GECCO cohort and **B** in the UK Biobank cohort. Blue line: the predicted models constructed with PRS previous GWAS-identified susceptibility loci; red line: the predicted models with PRS previous GWAS-identified susceptibility loci combined with EOCRC-specific risk variants. AUC and the point in the ROC curve identifying the best probability cutoff value (according to the Youden index) were presented. **C** Density plots of the PRS_CRC_ and **D** PRS_EOCRC+CRC_ for 1490 EOCRC cases (< 50 years) and 19,951 healthy controls in the GECCO cohort

We next evaluated the predictive accuracy of the model by estimating the area under the ROC curve (AUC). Compared with PRS_CRC_, the addition of identified EOCRC risk loci significantly increased discriminatory accuracy from 0.585 to 0.652 for the GECCO cohort, and AUC increased from 0.589 to 0.604 for the UK Biobank cohort (Fig. 4A and B). Discrimination of the PRS_EOCRC+CRC_ for EOCRC is reflected by less overlapping distributions between incident cases and controls compared with PRS_CRC_ (Fig. 4C and D). Considering the potential influences of other risk factors, we further adjusted several important sociodemographic factors in the prediction model in the UK Biobank cohort. The discrimination of the model-adjusted sociodemographic factors was 0.566 (95% CI: 0.544–0.587). Comparing with the model-adjusted sex and PRS_EOCRC+CRC_, the addition of the sociodemographic factors significantly increased the discrimination from 0.604 to 0.626 (Additional file 1: Fig. S7 and Additional file 2: Table S14). PRSs derived and validated here highlighted the potential for genomic screening and personalized risk assessment for EOCRC.

### The top EOCRC-specific risk variant rs12794623 acts as an active promoter of *POLA2* in an allele-specific manner

**Fig. 5:**
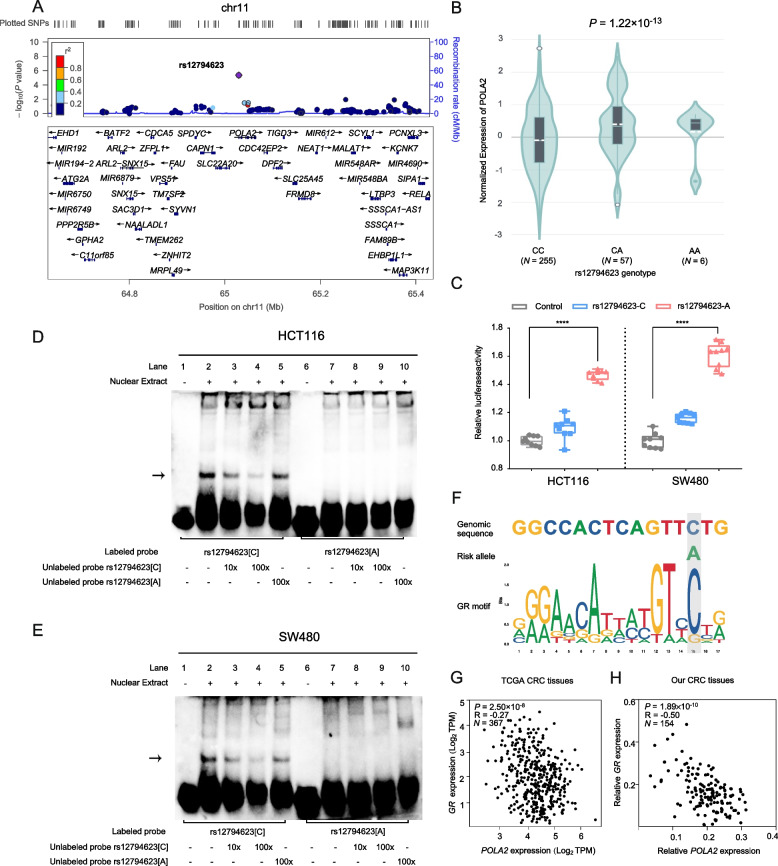
Biological interpretation of EOCRC risk variant rs12794623. **A** Regional plot of LD r^2^ and recombination rates in a 1-Mb region centered by rs12794623 based on the 1000 Genomes Nov 2014 EUR population. **B** The association between rs12794623 genotypes and *POLA2* expression using colon-sigmoid tissues in GTEx V8 database (dbGaP Accession phs000424.v8.p2). *P* values were calculated by a two-sided Student’s *t*-test. **C** Relative luciferase activity of fragments containing either the rs12794623[A] or rs12794623[C] allele in HCT116 and SW480 cells. ****P < 0.0001 by a two-sided Student’s *t*-test. **D** and **E** EMSAs with labeled probes containing rs12794623[A] or rs12794623[C] allele and nuclear extracts from HCT116 and SW480 cells. The arrow illustrated allele-specific differences in protein binding. **F** The predicted binding sites sequence of rs12794623 and GR motif using the JASPAR database. The rs12794623[C] allele showed a stronger motif binding possibility. **G** The negative correlation of *POLA2* with *GR* in TCGA COAD and READ tissues and **H** in our own CRC tissues. The expression levels of *POLA2* and *GR* were detected by qRT-PCR and normalized to that of *GAPDH*. All *P* values and R values were calculated by Spearman’s correlation analysis

To further acquire more insights into the genetic basis of EOCRC, we further functionally annotated those genetic variants by using multiple bioinformatic tools and databases, including the HaploReg, Roadmap Epigenomics Consortium, ENCODE, RegulomeDB, CADD, and 3DSNP databases, which integrated multiple histone modification ChIP-seq peaks and TF ChIP-seq peaks (Additional file 2: Table S15). Interestingly, the variant rs12794623 was revealed to be the most potential to be functional, and which was also the strongest EOCRC-specific signal identified (*P* = 4.41×10^−6^, OR = 1.33, Fig. 5A), apart from the previous GWAS locus in 1q41 region. The functional variant, rs12794623, located in the 5′UTR of *POLA2*, is a significant eQTL for this gene. Individuals carrying the rs12794623-A allele had higher *POLA2* expression than rs12794623-C allele carriers in colon sigmoid tissues from GTEx data (*P* = 1.22×10^−13^, Fig. 5B). We then conducted several experiments to evaluate its function. By transfecting plasmids containing different alleles of *POLA2* promoter in HCT116 and SW480 cells (Fig. 5C), we found the construct containing the rs12794623-A allele exhibits significantly higher luciferase activity than that containing rs12794623-C allele. Moreover, we performed electrophoretic mobility shift assays (EMSA) and found that the rs12794623 might alter transcription factors binding in an allele-specific manner (Fig. 5D and E). Based on the TF motif prediction in HaploReg [31], we supposed that the rs12794623-C allele might regulate *POLA2* expression via affecting GR binding (Fig. 5F), and *POLA2* expression was observed to be moderately correlated with *GR* expression in both TCGA CRC tissues (*P* = 2.50×10^−8^, *r* = −0.27) and our own CRC tissues (*P* = 1.89×10^−10^, *r* = −0.50) (Fig. 5G and H). Collectively, these results displayed that rs12794623 might allele-specifically influenced the expression of *POLA2* by the transcriptional regulation of GR.

### Overexpression of *POLA2* enhances CRC cell proliferation

**Fig. 6:**
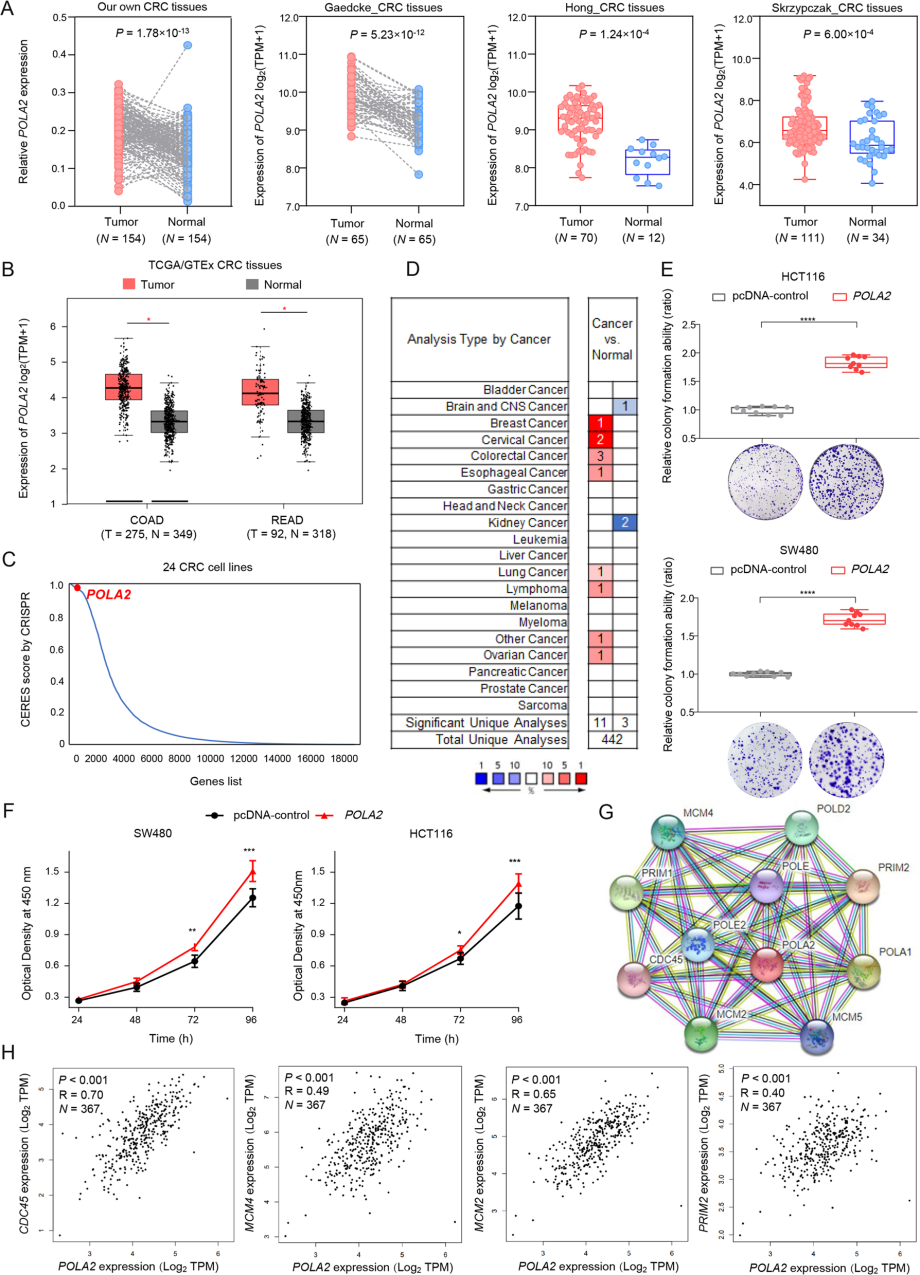
*POLA2* facilitates CRC progress by potential biological mechanisms. **A** and **B ***POLA2* was differential expressed between CRC tissues and peritumor tissues from multiple independent databases, including our own CRC tissues, Oncomine database (Gaedcke CRC tissues: GSE20842, Hong CRC tissues: GSE9348, Skrzypczak CRC tissues: GSE20916) and TCGA/GTEx CRC tissues. The expression data of *POLA2* was calculated by applying log_2_(TPM+1) log-scale in a public dataset, and the expression levels of *POLA2* were detected by qRT-PCR and normalized to that of *GAPDH* in our own CRC tissues. And *P* value was calculated by a two-sided Student’s *t*-test in TCGA/GTEx data, Hong CRC tissues, and Skrzypczak CRC tissues, whereas were calculated by a paired two-sided Student’s *t*-test in Gaedcke CRC tissues and our own CRC tissues. **C** Based on a genome-wide CRISPR-Cas9-based loss-of-function screen, *POLA2* was essential for cell growth; high CERES scores meant *POLA2* was essential for cell growth in 24 CRC cell lines. **D ***POLA2* expression levels were evaluated in multiple tumor tissue types from the Oncomine database. **E** The effect of overexpressing *POLA2* on colony formation ability in SW480 and HCT116 cells with three replicates. The results presented colony formation ability relative to the control group (set to 100%). Data were shown from three experiments and each with three replicates. ****P < 0.0001 was calculated by a two-sided Student’s *t*-test. **F** The effect of *POLA2* overexpression on cell proliferation in SW480 and HCT116 cells. Results were presented as the means ± SD from three experiments with six replicates each. All **P < 0.01, ***P < 0.001 compared with controls were calculated by a two-sided Student’s *t*-test. **G** The protein interaction network of *POLA2* by STRING database. **H ***POLA2* and the top 4 target mRNAs were co-expressed in CRC tissues based on TCGA data. All *P* values and R values were calculated by Pearson’s correlation analysis

Previous studies showed that *POLA2* played an important role in DNA replication [32]. To investigate the role of *POLA2* in EOCRC tumorigenesis, we first evaluated *POLA2* expression in tumor and adjacent normal tissues from multiple databases, including TCGA/GTEx samples, GEO datasets, and our own CRC patients. Results showed that *POLA2* significantly overexpressed in CRC tissues than in peritumoral tissues from our CRC samples (Fig. 6A), consistent with other databases’ results (Fig. 6A and B). Furthermore, we investigated the clinical significance of *POLA2* and found that higher TMB was associated with the higher expression of *POLA2* in TCGA CRC tissues (Additional file 1: Fig. S8). Furthermore, the CRISPR-Cas9-mediated loss-of-function screen data showed *POLA2* is likely to be one of the key genes affecting CRC cell survival functions (Fig. 6C) [33]. Data from the Oncomine database also suggested that *POLA2* amplification frequently occurred across cancer types (Fig. 6D). CCK-8 and colony formation assay indicated that overexpression of *POLA2* substantially increased the cell proliferation rate of CRC cells (Fig. 6E and F). The protein interaction network and co-expression analysis showed the interacted genes of *POLA2* played essential roles in DNA replication (Fig. 6G) [34]. Additional, *POLA2* co-expressed with DNA replication genes, *CDC45*, *MCM2*, *MCM4*, and *PRIM2*, in CRC tissues (Fig. 6H). Thus, these findings implied that *POLA2* might influence CRC carcinogenesis by affecting DNA replication.

## Discussion

In this study, we performed a large-scale GWAS for sporadic EOCRC, finding 49 EOCRC-specific risk loci. Candidate gene mapping and pathway analysis highlighted 88 potential functional genes and involved pathways in EOCRC. Based on these variants, PRS significantly improved risk prediction performance in both the GECCO and UKB cohorts. Besides, we demonstrated that the top EOCRC-specific variant rs12794623 regulated *POLA2* expression to mediate EOCRC tumorigenesis. Our work provided novel clues for downstream investigation into EOCRC etiology and potentially be applied in prevention and screening strategies.

Given that EOCRC incidence has been increasing worldwide [35], the genetic basis of EOCRC remains under-investigated [36, 37]. We performed two complementary GWASs focused on variants that not only contributed to EOCRC risk but also the onset age of CRC. As the result, 211 variants in 49 independent loci were identified as EOCRC-specific risk loci and most of them were first identified in CRC. We also found 16 variants were in LD with previously identified tagSNPs, indicating EOCRC and late-onset CRC shared partial genetic susceptibility [9]. We also validated 142 variants from the above 211 variants associated with EOCRC risk in both those under 50 years old and under 40 years old in the GECCO cohort. Furthermore, we found 84 variants (16 independent signals with *r*^2^ < 0.6) were significantly associated with younger EOCRC risk in 241 EOCRC cases (under 40 years) and 3,374 healthy controls. However, the sample size of EOCRC cases (under 40 years) was limited, future studies might especially focus on the EOCRC cases under 40 years old. Besides, we further mapped potential susceptibility genes for those 211 causal variants and found them enriched in chromatin assembly and DNA replication pathways (*H3C11*, *H2AC13*, and *H1-5* gene). It suggested that DNA replication disorders might contribute to the initial stage of EOCRC, which was confirmed by previous studies in early-onset cancers [38, 39]. Also, as the most associated disease of EOCRC susceptibility genes, precancerous polyps might be the precursor lesion of EOCRC [40, 41]. Additionally, patients with colorectal polyps were particularly at higher risk of EOCRC [42].

Construction of a PRS to evaluate the overall predictive power of common risk loci [43] for CRC has been carried out previously [44]. Although a recent study explored the prediction value of GWAS-identified genetic variants associated with EOCRC [9], limited information existed regarding the performance by adding specific susceptibility loci for sporadic EOCRC. By generating PRS and constructing risk prediction models of EOCRC, we found that the addition of EOCRC-specific risk variants combined with previously GWAS-identified genetic variants significantly improved the prediction accuracy of EOCRC in two large cohorts. These findings highlighted the potential utility of PRS in identifying high EOCRC-risk individuals. Meanwhile, the specific performance across both two cohorts might owe to the population heterogeneity, since racial disparities in the incidence of EOCRC and survival for colon cancer have been proven [45, 46], indicating that ethnically targeted early detection strategy should be adopted to screen high-risk people of EOCRC.

We further investigated the function of an identified variant, rs12794623, located in the 5’UTR region of *POLA2*. We found it regulated *POLA2* expression in an allele-specific manner via affecting the binding affinity of GR, which was recognized as an important tumor suppressor [47]. GR expression contributes to the recovery from intestinal inflammation by induction of tissue repair mechanisms after intestinal tissue damage [48]. As a DNA polymerase subunit, *POLA2* plays a crucial role at the G1 phase [49] by influencing DNA double-strand break repair, interacting with other DNA replication effectors [32]. Co-expression analysis in CRC tissues also showed that *POLA2* may interact with several DNA damage repair genes, including *MCM2*, *MCM10*, *CDC45*, and *PRIM2* [50]. These findings indicated an important role of DNA repair in EOCRC development, similarly with a previous retrospective review [51]. Furthermore, molecular pathologic analyses have been illustrated to be an important post-GWAS approach that can optimize individual prevention and therapy by focusing on the pathogenic process [52]. In the future, integrating the susceptibility gene expression with epigenetic alterations in EOCRC, such as MSI status [35, 36], CpG island methylator phenotype (CIMP) [53], and chromosomal instability [54], will elucidate the functional mechanisms of causal susceptibility and the plausible etiologic factors in the carcinogenic process.

As the most used strategy for CRC screening, the long-term effects of the fecal immunochemical test (FIT) and colonoscopy have been demonstrated [55]. Over the decades, widespread CRC screening among people over the age 55 years has contributed substantially to the decreasing CRC incidence and mortality [56, 57]. Due to the rising incidence of early-onset CRC, the US Preventive Services Task Force (USPSTF) [58], the US Multi-Society Task Force [59], and the American Cancer Society (ACS) [60] successively recommend starting CRC screening at age 45–50 years. Those recommendation has aroused intense discussion focusing on the potential benefits, liabilities, and economic outcomes [61]. It can be predicted that the cost-effectiveness of early-onset CRC screening programs and the efficacy of the healthcare systems will face major challenges [62, 63]. Here, we identified several genetic causal variants and constructed the prediction models of early-onset CRC, which might help to tentatively develop personalized screening strategies. The aim of this study was to precisely filter the people with high risks of early-onset CRC and promote the most cost-effective strategy for targeted screening in high-risk individuals. In the future, large-scale longitudinal studies can demonstrate long-term exposures from early life to adulthood and contribute to the advancements in precision prevention, combining with prospective biospecimen collections, multi-omics integration, and molecular pathological epidemiology, immunity, and tumor microenvironment analyses [64].

This study had several strengths. It is the first GWAS for sporadic EOCRC with a multi-stage design in a large population. The construction and evaluation of the PRS risk model in the UKB cohort can help with early screening and individualized treatment of EOCRC. We also revealed the potential regulation of a risk variant in EOCRC progression. However, there were some limitations. A major limitation of this study was the lack of stratification in the family history (Lynch syndromes and other rare hereditary CRC syndromes) and molecular pathologic classification (MSI status and CIMP) [65]. Second, although we analyzed the potential effects of several important sociodemographic factors in the risks of EOCRC, other potential environmental risk factors of EOCRC need to be considered in the GWAS stages. Third, the functional roles of other identified EOCRC risk variants are warranted to be investigated by high-throughput experimental methods in the future. At last, rare alleles were excluded, which might have a high impact on the risk of EOCRC [66]. Specific analysis for the genetic burden of rare mutations needs to be performed in the future.

## Conclusions

We systematically investigated the specific susceptibility of EOCRC and assessed the prediction value of identified loci in diverse populations. Moreover, leveraging on functional assays, we elucidated the biological plausibility of a top EOCRC-specific signal. These findings highlighted the underlying mechanism of EOCRC tumorigenesis, which might help to empower early prevention and detection strategies.

## Supplementary Information

**Additional file 1: **Additional figures from the results of early-onset colorectal cancer genetic susceptibility analysis. **Fig. S1.** 3D Plots for genetic matching of three principal components. **Fig. S2.** Determination of the *POLA2* transfection efficiency. **Fig. S3.** Quantile-quantile plot and genomic inflation factor lambda for associations with early-onset CRC risk. **Fig. S4.** Regional plots of association results and recombination rates within the four significant susceptibility loci. **Fig. S5.** Manhattan plots for associations between genetic variants and EOCRC risk under 40 years old. **Fig. S6.** Risk estimates for EOCRC (under 40 years old) associated with the PRS deriving from distinct SNP. **Fig. S7.** Risk estimates for EOCRC associated with the PRSs under the adjustment of sociodemographic factors in the UKB cohort. **Fig. S8.** Higher tumor mutational burden was associated with the higher expression of *POLA2* in TCGA CRC tissues.

**Additional file 2: **Additional tables from the results of early-onset colorectal cancer genetic susceptibility analysis. **Table S1.** Demographic characteristics of cases with early-onset CRC and controls in the GECCO cohort of this study. **Table S2.** Demographic characteristics of cases with early-onset CRC (under 40 years old) and matching controls in the GECCO cohort of this study. **Table S3.** Demographic characteristics of cases with early-onset CRC and controls in the UK Biobank cohort of this study. **Table S4.** Demographic characteristics of cases with early-onset CRC (under 40 years old) and controls in the UK Biobank cohort of this study. **Table S5.** The previous CRC GWAS loci were calculated in the PRS model. **Table S6.** Probes or primers sequence used in the study. **Table S7.** 211 Newly identified variants associated with early-onset CRC risk in the GECCO cohort. **Table S8.** 84 newly identified variants associated with EOCRC risk (under 40 years old) in the GECCO cohort. **Table S9.** Results for 16 identified variants associated with EOCRC risk (under 40 years old) in the GECCO cohort (LD *r*^2^ < 0.6). **Table S10.** The variants associated with early-onset CRC risk in both two age cut-off groups in the GECCO cohort. **Table S11.** 88 mapped genes of 211 EOCRC specific risk loci. **Table S12.** Risk estimates for early-onset CRC associated with different PRS scores in the GECCO cohort. **Table S13.** Risk estimates for early-onset CRC associated with different PRS scores in the UKB cohort. **Table S14.** Risk estimates for early-onset CRC associated with different PRS scores with adjustment of sociodemographic factors in the UKB cohort. **Table S15.** The potential functional annotations of the 49 candidate variants.

**Additional file 3.** The codes used for statistical analysis and generation of tables and figures.

## Data Availability

Publicly available datasets were used in this study. GECCO genotype data were obtained from the database of Genotypes and Phenotypes (dbGaP) under accession numbers phs001078.v1.p1, phs001315.v1.p1, and phs001415.v1.p1 (https://www.ncbi.nlm.nih.gov/projects/gap/cgi-bin/study.cgi?study_id=phs001078.v1.p1; https://www.ncbi.nlm.nih.gov/projects/gap/cgi-bin/study.cgi?study_id=phs001315.v1.p1; https://www.ncbi.nlm.nih.gov/projects/gap/cgi-bin/study.cgi?study_id=phs001415.v1.p1) [16]. Our accession to the UK Biobank data was through application No.51539 (https://biobank.ndph.ox.ac.uk/showcase/). All data generated or analyzed during this study are included in this published article and its supplementary information files. Example code to run principal component analysis (PCA), association analysis, polygenic risk score, and prediction model validation is given in Supplementary Information: Text S1. Other web resources used in this article are listed: Michigan Imputation Server, https://imputationserver.sph.umich.edu/index.html#!; TCGA database, https://portal.gdc.cancer.gov; LocusZoom, http://locuszoom.sph.umich.edu/; RegulomeDB Score: https://www.regulomedb.org/regulome-search; CADD Score: https://cadd.gs.washington.edu/score; 3DSNP Score: https://omic.tech/3dsnpv2/; HaploReg v4.1, https://pubs.broadinstitute.org/mammals/haploreg/haploreg.php; JASPAR database, https://jaspar.genereg.net/; GTEx V8 database, (dbGaP Accession phs000424.v8.p2), https://www.gtexportal.org/home/.

## Abbreviations

CRC: Colorectal cancer
EOCRC: Early-onset colorectal cancer
GWAS: Genome-wide association study
CI: Confidence interval
OR: Odds ratio
PRS: Polygenic risk score
PCA: Principal component analysis
SD: Standard deviation
AUC: Area under the ROC curve
TF: Transcription factor
ChIP: Chromatin immunoprecipitation
