# Supplementary material for: Identification of specific susceptibility loci for the early-onset colorectal cancer
Source: Genome Med. 2023 Mar 3;15:13. doi: 10.1186/s13073-023-01163-w (PMC9983269; doi:10.1186/s13073-023-01163-w)
Supplement: Supplementary file 1 — Additional file 1: Additional figures from the results of early-onset colorectal cancer genetic susceptibility analysis. Fig. S1. 3D Plots for genetic matching of three principal components. Fig. S2. Determination of the POLA2 transfection efficiency. Fig. S3. Quantile-quantile plot and genomic inflation factor lambda for associations with early-onset CRC risk. Fig. S4. Regional plots of association results and recombination rates within the four significant susceptibility loci. Fig. S5. Manhattan plots for associations between genetic variants and EOCRC risk under 40 years old. Fig. S6. Risk estimates for EOCRC (under 40 years old) associated with the PRS deriving from distinct SNP. Fig. S7. Risk estimates for EOCRC associated with the PRSs under the adjustment of sociodemographic factors in the UKB cohort. Fig. S8. Higher tumor mutational burden was associated with the higher expression of POLA2 in TCGA CRC tissues. [file 13073_2023_1163_MOESM1_ESM.docx]

**Identification of specific susceptibility loci for the early-onset colorectal cancer**

**Additional file 1**

[Fig. S1. 3D Plots for genetic matching of three principal components. 2](#_Toc126934405)

[Fig. S2. Determination of the POLA2 transfection efficiency. 3](#_Toc126934406)

[Fig. S3. Quantile-quantile plot and genomic inflation factor lambda for associations with early-onset CRC risk. 4](#_Toc126934407)

[Fig. S4. Regional plots of association results and recombination rates within the four significant susceptibility loci. 5](#_Toc126934408)

[Fig. S5. Manhattan plots for associations between genetic variants and EOCRC risk under 40 years old. 6](#_Toc126934409)

[Fig. S6. Risk estimates for EOCRC (under 40 years old) associated with the PRS deriving from distinct SNPs. 7](#_Toc126934410)

[Fig. S7. Risk estimates for EOCRC associated with the PRSs under the adjustment of sociodemographic factors in the UKB cohort 8](#_Toc126934411)

[Fig. S8. Higher tumor mutational burden was associated with the higher expression of *POLA2* in TCGA CRC tissues 10](#_Toc126934412)

**
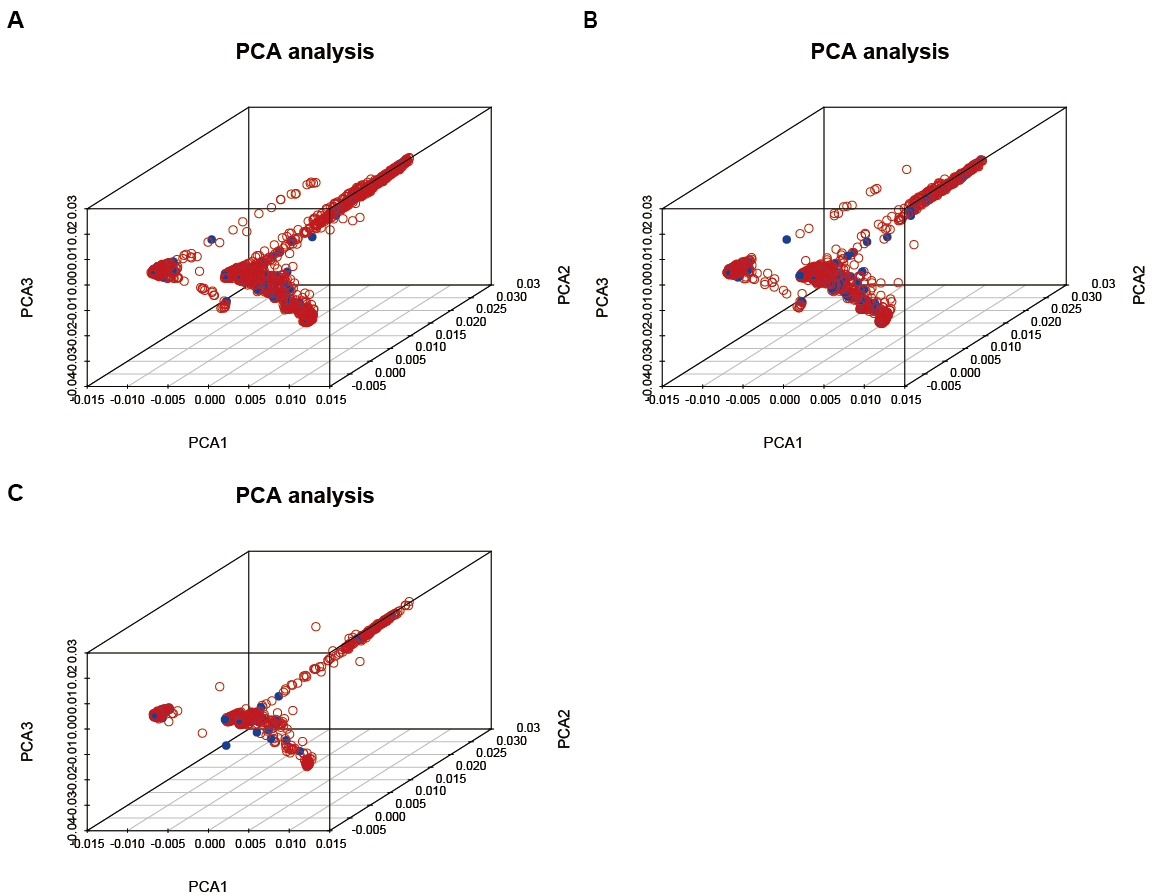
**

# Fig. S1. 3D Plots for genetic matching of three principal components.

**A.** derived from the PCA of 1490 cases with early-onset CRC under 50 years old and 19951 controls, **B.** 17,789 CRC cases, and **C.** 241 early-onset CRC cases under 40 years old and 3,374 controls

**
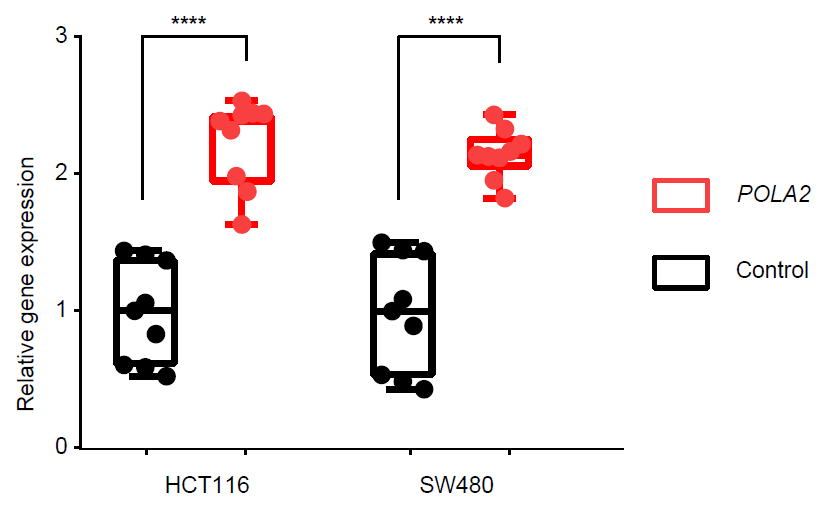
**

# Fig. S2. Determination of the POLA2 transfection efficiency.

Relative expression levels of *POLA2* in SW480 and HCT116 cell lines were determined by qRT-PCR assays. Data were presented from three experiments, each with three replicates and all *****P* < 0.0001 were calculated by a two-sided Student's *t*-test.

**
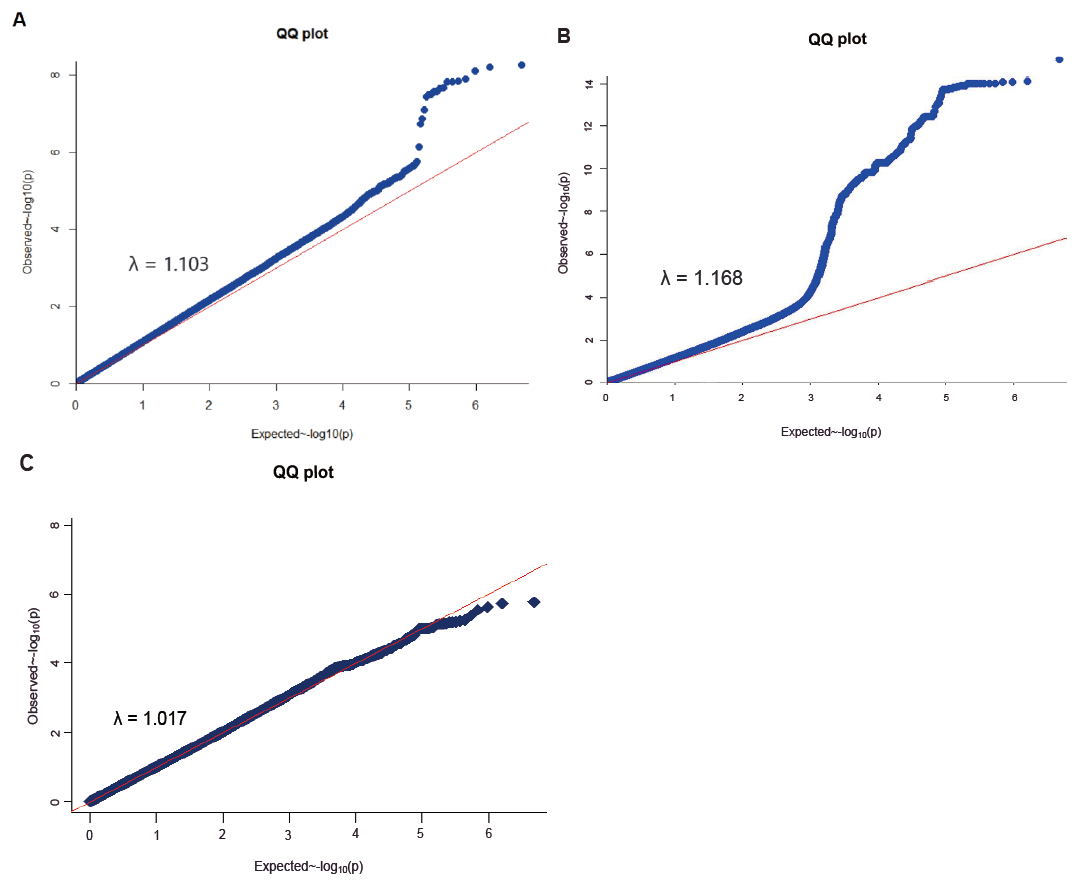
**

# Fig. S3. Quantile-quantile plot and genomic inflation factor lambda for associations with early-onset CRC risk.

A. The results were based on in 1,490 early-onset CRC cases under 50 years old and 19,951 controls in the first analysis of this study. B. The results were based on in 17,789 CRC cases in the second analysis of this study. C. The results were based on in 241 early-onset CRC cases under 40 years old and 3,374 controls in the first analysis of this study. The blue circles represent the distribution of *P* values for the association. The observed versus expected χ^2^ test statistics shows no evidence for inflation of χ^2^ tests (inflation factor λ = 1.103, 1.168 and 1.017).


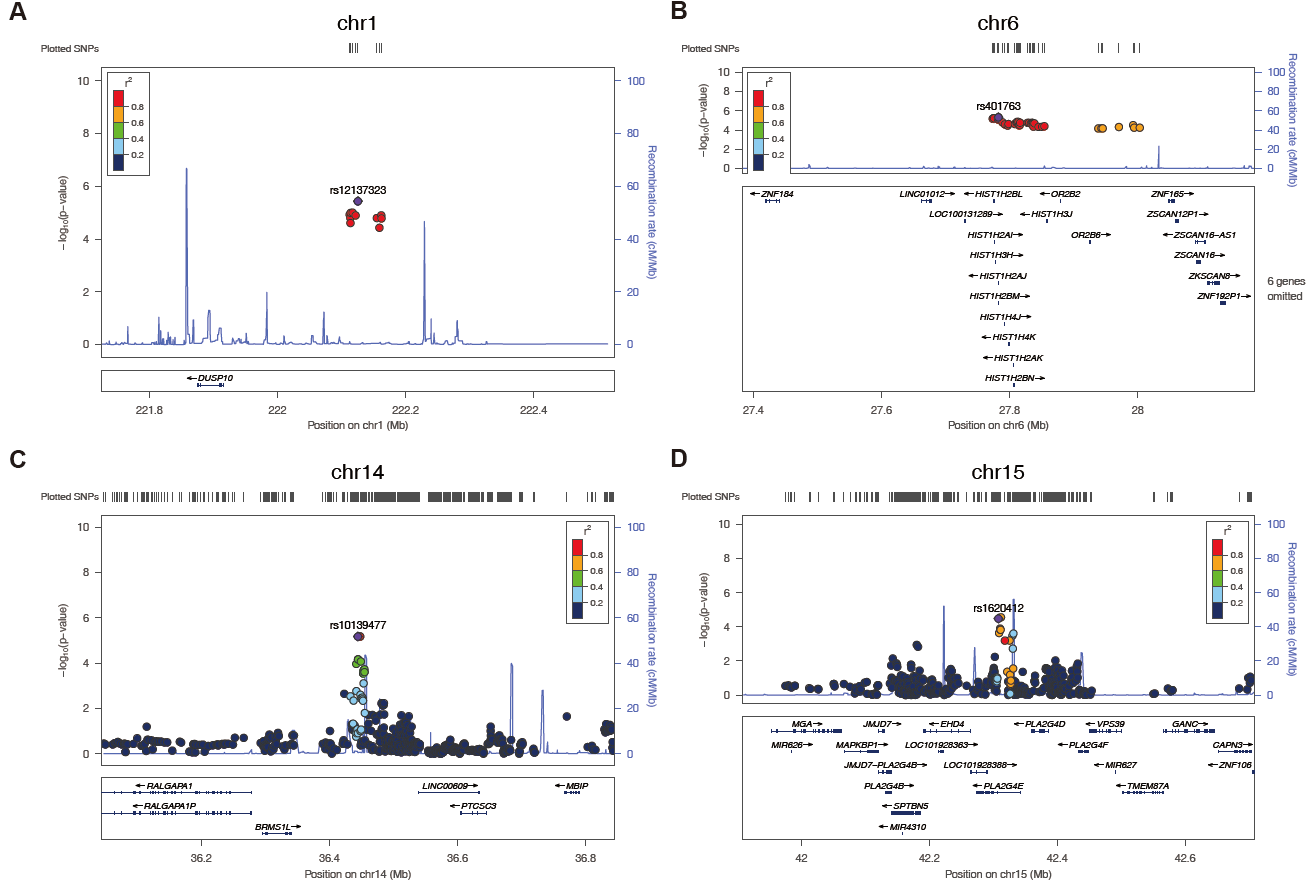


# Fig. S4. Regional plots of association results and recombination rates within the four significant susceptibility loci.

A. rs12137323 B. rs401763 C. rs10139477 D. rs1620412. The association results were based on imputation results of 1,490 early-onset CRC cases and 19,951 controls in the first analysis of this study. *P* values are two sided and were calculated by an additive model in logistic regression analysis adjusted for sex, recruitment center and 10 principal components. For each plot, the −log_10_ *P* values (y-axis) of the SNPs are presented according to their chromosomal positions (x-axis). The genetic recombination rates (cM/Mb) estimated based on the 1000 Genomes Nov 2014 EUR population are shown with a blue line. We annotated the genes within the region of interest, and these genes are shown as arrows. The LD r^2^ values were calculated using pairwise linkage disequilibrium analyses. The top genotyped SNP is labeled by rs ID, and the r^2^ values of the rest of the SNPs with the top genotyped SNP are indicated by different colors.


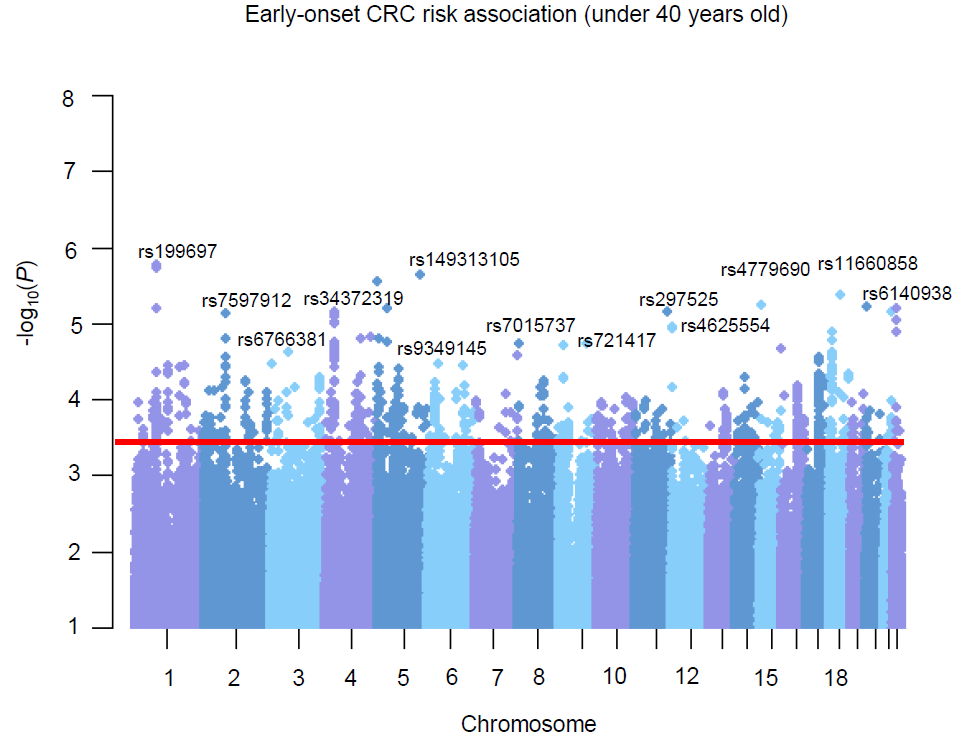


# Fig. S5. Manhattan plots for associations between genetic variants and EOCRC risk under 40 years old.

The logistic regression analysis of 241 EOCRC cases and 3,374 controls. *P* values are two-sided, calculated by an additive model and adjusted for sex, recruitment center and the 10 principal components. The red line indicates the genome-wide significance threshold. The associations (–log_10_(*P*) values, y-axis) are plotted against genomic position (x-axis by chromosome and chromosomal position of NCBI build 37).


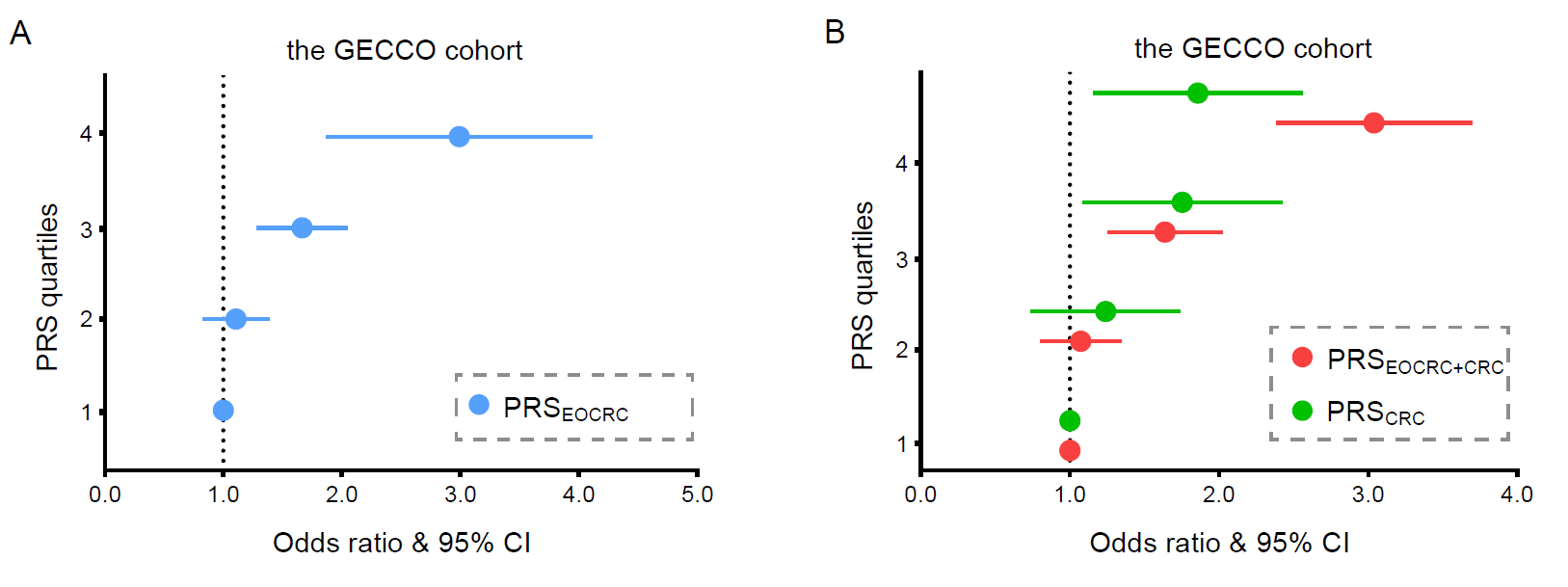


# Fig. S6. Risk estimates for EOCRC (under 40 years old) associated with the PRS deriving from distinct SNPs.

**A.** The PRS was generated by calculating the effect sizes of EOCRC specific risk loci in 241 EOCRC cases (< 40 years) and 3,374 healthy controls in the GECCO cohort and **B.** Two PRS models were deriving from 86 previously GWAS-identified CRC risk SNPs and those 86 SNPs combined with 16 EOCRC (< 40 years) specific SNPs in 241 EOCRC cases (< 40 years) and 3,374 healthy controls in the GECCO cohort, respectively. Models were adjusted for sex and PRS quartiles. The PRS scores were modeled as a continuous variable per 1 standard deviation (SD), transformed to the standard normal distribution. ORs and 95% confidence intervals were estimated comparing quartiles of PRS. The dashed line indicated the odds ratio = 1 as reference.

**
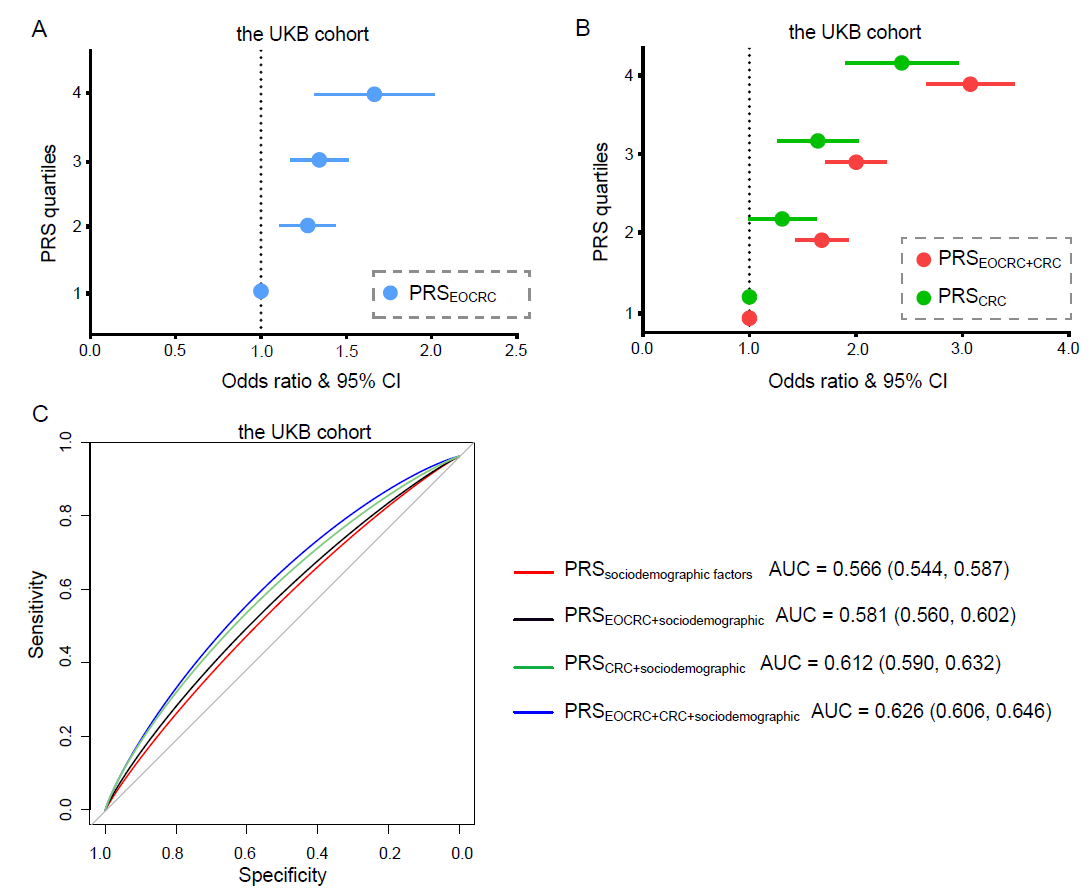
**

# Fig. S7. Risk estimates for EOCRC associated with the PRSs under the adjustment of sociodemographic factors in the UKB cohort

**A.** The PRS was generated by calculating the effect sizes of EOCRC specific risk loci in the UKB cohort. **B.** Two PRS models were deriving from 86 previously GWAS-identified CRC risk SNPs and the 86 SNPs combined with 49 SNPs in the UKB cohort, respectively. Models were adjusted for sex, race, drinking frequency, smoking status, and family history of bowel cancer and PRS quartiles. The PRS scores were modeled as a continuous variable per 1 standard deviation (SD), transformed to the standard normal distribution. ORs and 95% confidence intervals were estimated comparing quartiles of PRS. **C.** In the UK Biobank cohort, ROC curves for the distinct predicted models with PRS and several risk factors, respectively. PRS_sociodemographic factors_: Models were adjusted for sociodemographic factors (sex, race, drinking frequency, smoking status, and family history of bowel cancer); PRS_EOCRC+sociodemographic_: Models were adjusted for sociodemographic factors and PRS_EOCRC_ quartiles; PRS_CRC+sociodemographic_: Models were adjusted for sociodemographic factors and PRS_CRC_ quartiles; PRS_EOCRC+CRC+sociodemographic_: Models were adjusted for sociodemographic factors and PRS_EOCRC+CRC_ quartiles. AUC and the point in the ROC curve identifying the best probability cutoff value (according to the Youden index) were presented


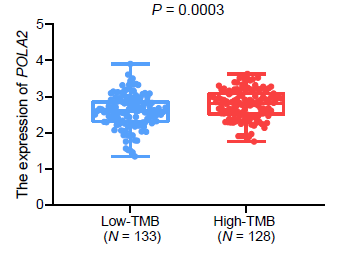


# Fig. S8. Higher tumor mutational burden was associated with the higher expression of *POLA2* in TCGA CRC tissues

The data of tumor mutational burden (TMB) and gene expression data were obtained from COAD and READ tissues in the TCGA database (https://portal.gdc.cancer.gov). The samples included 261 CRC tissue samples. TMB per megabase is calculated by dividing the total number of mutations by the size of the coding region of the target. Based on the median TMB value (2.02), the TMB group was divided into a high-TMB group and a low-TMB group. *P* = 0.0003 were calculated by a two-sided Student's *t*-test.
