# Supplementary material for: Identification of specific susceptibility loci for the early-onset colorectal cancer
Source: Genome Med. 2023 Mar 3;15:13. doi: 10.1186/s13073-023-01163-w (PMC9983269; doi:10.1186/s13073-023-01163-w)
Supplement: Supplementary file 2 — Additional file 2: Additional tables from the results of early-onset colorectal cancer genetic susceptibility analysis. Table S1. Demographic characteristics of cases with early-onset CRC and controls in the GECCO cohort of this study. Table S2. Demographic characteristics of cases with early-onset CRC (under 40 years old) and matching controls in the GECCO cohort of this study. Table S3. Demographic characteristics of cases with early-onset CRC and controls in the UK Biobank cohort of this study. Table S4. Demographic characteristics of cases with early-onset CRC (under 40 years old) and controls in the UK Biobank cohort of this study. Table S5. The previous CRC GWAS loci were calculated in the PRS model. Table S6. Probes or primers sequence used in the study. Table S7. 211 Newly identified variants associated with early-onset CRC risk in the GECCO cohort. Table S8. 84 newly identified variants associated with EOCRC risk (under 40 years old) in the GECCO cohort. Table S9. Results for 16 identified variants associated with EOCRC risk (under 40 years old) in the GECCO cohort (LD r2 < 0.6). Table S10. The variants associated with early-onset CRC risk in both two age cut-off groups in the GECCO cohort. Table S11. 88 mapped genes of 211 EOCRC specific risk loci. Table S12. Risk estimates for early-onset CRC associated with different PRS scores in the GECCO cohort. Table S13. Risk estimates for early-onset CRC associated with different PRS scores in the UKB cohort. Table S14. Risk estimates for early-onset CRC associated with different PRS scores with adjustment of sociodemographic factors in the UKB cohort. Table S15. The potential functional annotations of the 49 candidate variants. [file 13073_2023_1163_MOESM2_ESM.docx]

**Identification of specific susceptibility loci for the early-onset colorectal cancer**

**Additional file 2**

[Table. S1. Demographic characteristics of cases with early-onset CRC and controls in the GECCO cohort of this study. 2](#_Toc126934232)

[Table. S2. Demographic characteristics of cases with early-onset CRC (under 40 years old) and matching controls in the GECCO cohort of this study. 3](#_Toc126934233)

[Table. S3. Demographic characteristics of cases with early-onset CRC and controls in the UK Biobank cohort of this study. 4](#_Toc126934234)

[Table. S4. Demographic characteristics of cases with early-onset CRC (under 40 years old) and controls in the UK Biobank cohort of this study. 5](#_Toc126934235)

[Table. S5. The previous CRC GWAS loci were calculated in the PRS model. 7](#_Toc126934236)

[Table. S6. Probes or primers sequence used in the study. 10](#_Toc126934237)

[Table. S7. 211 Newly identified variants associated with early-onset CRC risk in the GECCO cohort. 11](#_Toc126934238)

[Table. S8. 84 newly identified variants associated with EOCRC risk (under 40 years old) in the GECCO cohort. 19](#_Toc126934239)

[Table. S9. Results for 16 identified variants associated with EOCRC risk (under 40 years old) in the GECCO cohort (LD r^2^ < 0.6). 23](#_Toc126934240)

[Table. S10. The variants associated with early-onset CRC risk in both two age cut-off groups in the GECCO cohort. 24](#_Toc126934241)

[Table. S11. 88 mapped genes of 211 EOCRC specific risk loci. 29](#_Toc126934242)

[Table. S12. Risk estimates for early-onset CRC associated with different PRS scores in the GECCO cohort. 33](#_Toc126934243)

[Table. S13. Risk estimates for early-onset CRC associated with different PRS scores in the UKB cohort. 34](#_Toc126934244)

[Table. S14. Risk estimates for early-onset CRC associated with different PRS scores with adjustment of sociodemographic factors in the UKB cohort. 35](#_Toc126934245)

[Table. S15. The potential functional annotations of the 49 candidate variants. 36](#_Toc126934246)

# Table. S1. Demographic characteristics of cases with early-onset CRC and controls in the GECCO cohort of this study.

|  | Early-onset CRC risk association | | | | |  | Age related CRC risk association | | | | |
| --- | --- | --- | --- | --- | --- | --- | --- | --- | --- | --- | --- |
|  | Early-onset CRC | (%) | Control | (%) | *P* |  | Early-onset CRC | (%) | Late-onset CRC | (%) | *P* |
| Total number | 1490 |  | 19951 |  |  |  | 1490 |  | 16299 |  |  |
| Age,mean±SD | 44.09 ± 4.99 |  | 59.97 ± 12.32 |  | <0.001^†^ |  | 44.09 ± 4.99 |  | 65.64 ± 8.66 |  | <0.001^†^ |
| Gender |  |  |  |  | 0.485^*^ |  |  |  |  |  | <0.001^*^ |
| Male | 755 | (50.7) | 10298 | (51.6) |  |  | 755 | (50.7) | 9373 | (57.5) |  |
| Female | 735 | (49.3) | 9653 | (48.4) |  |  | 735 | (49.3) | 6926 | (42.5) |  |

^*^*P* value were assessed by Pearson χ^2^ test; ^†^*P* value were assessed by Student’s *t*-test.

# Table. S2. Demographic characteristics of cases with early-onset CRC (under 40 years old) and matching controls in the GECCO cohort of this study.

|  | Early-onset CRC risk association under 40 | | | | |  |
| --- | --- | --- | --- | --- | --- | --- |
|  | Early-onset CRC | (%) | Control | (%) | *P* |  |
| Total number | 241 |  | 3374 |  |  |  |
| Age,mean±SD | 35.3 ± 4.27 |  | 41.04 ± 8.76 |  | <0.001^†^ |  |
| Gender |  |  |  |  | 0.424^*^ |  |
| Male | 115 | (47.7) | 1700 | (50.4) |  |  |
| Female | 126 | (52.3) | 1674 | (49.6) |  |  |

^*^*P* value were assessed by Pearson χ^2^ test; ^†^*P* value were assessed by Student’s *t*-test.

# Table. S3. Demographic characteristics of cases with early-onset CRC and controls in the UK Biobank cohort of this study.

|  | Early-onset CRC | (%) | Control | (%) | *P* |
| --- | --- | --- | --- | --- | --- |
| Total number | 723 |  | 24427 |  |  |
| Age,mean±SD | 42.98 ± 6.43 |  | 57.25 ± 7.97 |  | <0.001^†^ |
| Gender |  |  |  |  | <0.001^*^ |
| Male | 270 | (37.3) | 11430 | (46.8) |  |
| Female | 453 | (62.7) | 12997 | (53.2) |  |
| Ethnicity |  |  |  |  | 0.955^*^ |
| White^a^ | 690 | (95.4) | 23244 | (95.2) |  |
| Mixed^b^ | 4 | (0.6) | 125 | (0.5) |  |
| Asian or Asian British^c^ | 12 | (1.7) | 486 | (2.0) |  |
| Black or Black British^d^ | 12 | (1.7) | 370 | (1.5) |  |
| Other ethnic group | 5 | (0.7) | 202 | (0.8) |  |
| Alcohol intake frequency^e^ |  |  |  |  | 0.765^*^ |
| Heavy consumption | 313 | (43.3) | 10789 | (44.2) |  |
| Moderate consumption | 349 | (48.3) | 11674 | (47.8) |  |
| Never | 61 | (8.4) | 1938 | (7.9) |  |
| Smoking status^f^ |  |  |  |  | 0.294^*^ |
| Current | 86 | (11.9) | 2517 | (10.3) |  |
| Former | 241 | (33.4) | 8593 | (35.3) |  |
| Never | 395 | (54.7) | 13217 | (54.3) |  |
| CRC family history |  |  |  |  | <0.001^*^ |
| Yes | 110 | (15.2) | 2698 | (11.0) |  |
| No | 613 | (84.8) | 21729 | (89.0) |  |

^*^*P* value were assessed by Pearson χ^2^ test; ^†^*P* value were assessed by Student’s *t*-test.

^a^ included white British, lrish, and other white background.

^b^ included White and Black Caribbean, White and Black African, White and Asian and any other mixed background

^c^ included Indian, Pakistani, Bangladeshi, Chinese, and any other Asian background

^d^ included Caribbean, African, any other Black background

^e,f^ Participants prefer not to answer the item: alcohol intake frequency (*N* = 26) and smoking status (*N* = 101).

# Table. S4. Demographic characteristics of cases with early-onset CRC (under 40 years old) and controls in the UK Biobank cohort of this study.

|  | Early-onset CRC | (%) | Control | (%) | *P* |
| --- | --- | --- | --- | --- | --- |
| Total number | 185 |  | 24427 |  |  |
| Age,mean±SD | 33.92 ± 5.35 |  | 57.25 ± 7.97 |  | <0.001^†^ |
| Gender |  |  |  |  | <0.001^*^ |
| Male | 72 | (38.9) | 11430 | (46.8) |  |
| Female | 113 | (61.1) | 12997 | (53.2) |  |
| Ethnicity |  |  |  |  | 0.885^*^ |
| White^a^ | 178 | (96.2) | 23244 | (95.2) |  |
| Mixed^b^ | 1 | (0.5) | 350 | (1.4) |  |
| Asian or Asian British^c^ | 2 | (1.1) | 486 | (2.0) |  |
| Black or Black British^d^ | 2 | (1.1) | 145 | (0.6) |  |
| Other ethnic group | 2 | (1.1) | 202 | (0.8) |  |
| Alcohol intake frequency^e^ |  |  |  |  | 0.777^*^ |
| Heavy consumption | 76 | (41.1) | 10789 | (44.2) |  |
| Moderate consumption | 92 | (49.7) | 11674 | (47.8) |  |
| Never | 17 | (9.2) | 1938 | (7.9) |  |
| Smoking status^f^ |  |  |  |  | 0.318^*^ |
| Current | 24 | (13.0) | 2517 | (10.3) |  |
| Former | 56 | (30.3) | 8593 | (35.3) |  |
| Never | 105 | (56.8) | 13217 | (54.3) |  |
| CRC family history |  |  |  |  | 0.405^*^ |
| Yes | 24 | (13.0) | 2698 | (11.0) |  |
| No | 161 | (87.0) | 21729 | (89.0) |  |

^*^*P* value were assessed by Pearson χ^2^ test; ^†^*P* value were assessed by Student’s *t*-test.

^a^ included white British, lrish, and other white background.

^b^ included White and Black Caribbean, White and Black African, White and Asian and any other mixed background

^c^ included Indian, Pakistani, Bangladeshi, Chinese, and any other Asian background

^d^ included Caribbean, African, any other Black background

^e,f^ Participants prefer not to answer the item: alcohol intake frequency (*N* = 26) and smoking status (*N* = 100).

# Table. S5. The previous CRC GWAS loci were calculated in the PRS model.

| SNP | Locus | Chr. | Position | Nearby gene(s) | OR | 95% CI | *P* |
| --- | --- | --- | --- | --- | --- | --- | --- |
| rs72647484 | 1p36.12 | 1 | 22587728 |  | 1.24 | 1.15-1.33 | 1.0×10^-8^ |
| rs4360494 | 1p34.3 | 1 | 38455891 | *SF3A3* | 1.05 | 1.04-1.07 | 3.8×10^-9^ |
| rs12144319 | 1p32.3 | 1 | 55246035 | *TTC22* | 1.07 | 1.05-1.09 | 3.3×10^-11^ |
| rs12143541 | 1p32.3 | 1 | 55247852 | *TTC22* | 1.15 | 1.10-1.20 | 4.0×10^-9^ |
| rs7542665 | 1p31.3 | 1 | 62673037 | *L1TD1* | 1.09 | 1.06-1.12 | 3.5×10^-8^ |
| rs6678517 | 1q25.3 | 1 | 183002639 | *LAMC1* | 1.08 | 1.06-1.09 | 2.4×10^-16^ |
| rs6691170 | 1q41 | 1 | 222045446 |  | 1.06 | 1.03-1.09 | 1.0×10^-8^ |
| rs17011141 | 1q41 | 1 | 222112634 |  | 1.09 | 1.07-1.12 | 6.1×10^-16^ |
| rs448513 | 2q24.2 | 2 | 159964552 | *TANC1* | 1.05 | 1.03-1.07 | 4.4×10^-8^ |
| rs11893063 | 2q33.1 | 2 | 199601925 |  | 1.15 | 1.10-1.20 | 9.3×10^-9^ |
| rs35470271 | 3p22.1 | 3 | 40915239 |  | 1.1 | 1.08-1.13 | 1.2×10^-16^ |
| rs9831861 | 3p21.1 | 3 | 53088285 |  |  |  | 4.2×10^-10^ |
| rs72942485 | 3q13.2 | 3 | 112999560 | *BOC* | 1.19 | 1.12-1.26 | 2.1×10^-8^ |
| rs10049390 | 3q22.2 | 3 | 133701119 | *SLCO2A1* | 1.06 | 1.04-1.08 | 3.8×10^-9^ |
| rs9876206 | 3q26.2 | 3 | 169517436 | *LRRC34* | 1.05 | 1.03-1.07 | 7.8×10^-6^ |
| rs17035289 | 4q24 | 4 | 106048291 |  | 1.15 | 1.10-1.20 | 2.7×10^-10^ |
| rs35509282 | 4q32.2 | 4 | 163333405 |  | 1.53 | 1.39-1.67 | 8.2×10^-9^ |
| rs12514517 | 5p13.1 | 5 | 40280076 |  | 1.1 | 1.08-1.12 | 3.7×10^-21^ |
| rs1862626 | 5q11.2 | 5 | 56032940 |  |  |  | 4.0×10^-12^ |
| rs254563 | 5q31.1 | 5 | 134440426 | *C5orf66* | 1.12 | 1.08-1.16 | 1.2×10^-9^ |
| rs4976270 | 5q31.1 | 5 | 134467220 | *C5orf66* | 1.07 | 1.05-1.09 | 4.8×10^-15^ |
| rs639933 | 5q31.1 | 5 | 134467751 | *C5orf66* |  |  | 4.2×10^-10^ |
| rs3131043 | 6p21.33 | 6 | 30758466 |  | 1.14 | 1.09-1.18 | 2.7×10^-8^ |
| rs3830041 | 6p21.32 | 6 | 32191339 | *NOTCH4* |  |  | 1.6×10^-8^ |
| rs9470361 | 6p21.2 | 6 | 36623379 |  | 1.06 | 1.03-1.08 | 8.6×10^-8^ |
| rs4711689 | 6p21.1 | 6 | 41692812 | *TFEB* | 1.11 | 1.07-1.15 | 3.9×10^-8^ |
| rs62404966 | 6p12.1 | 6 | 55712124 | *BMP5* | 1.06 | 1.04-1.09 | 2.6×10^-9^ |
| rs12672022 | 7p13 | 7 | 45136423 | *TBRG4* | 1.07 | 1.04-1.09 | 2.8×10^-8^ |
| rs3801081 | 7p12.3 | 7 | 47511161 | *TNS3* |  |  | 2.0×10^-11^ |
| rs10505477 | 8q24.21 | 8 | 128407443 | *CASC8* | 1.15 | 1.11-1.20 | 3.0×10^-13^ |
| rs7014346 | 8q24.21 | 8 | 128424792 | *CASC8* | 1.13 | 1.08-1.17 | 2.0×10^-8^ |
| rs1412834 | 9p21.3 | 9 | 22110131 | *CDKN2B-AS1* | 1.15 | 1.10-1.20 | 4.0×10^-9^ |
| rs9409565 | 9q22.32 | 9 | 97251034 |  | 1.35 | 1.21-1.49 | 2.0×10^-8^ |
| rs34405347 | 9q22.33 | 9 | 101679752 |  | 1.09 | 1.05-1.12 | 3.1×10^-8^ |
| rs10980628 | 9q31.3 | 9 | 113671403 | *LPAR1* | 1.07 | 1.04-1.09 | 2.8×10^-9^ |
| rs4143094 | 10p14 | 10 | 8089136 | *GATA3-AS1* | 1.17 | 1.11-1.23 | 9.0×10^-9^ |
| rs704017 | 10q22.3 | 10 | 80819132 | *ZMIZ1-AS1* | 1.07 | 1.06-1.10 | 5.2×10^-18^ |
| rs6584283 | 10q24.2 | 10 | 101290301 | *LINC01475* |  |  | 4.2×10^-10^ |
| rs11190164 | 10q24.2 | 10 | 101351704 |  | 1.09 | 1.06-1.10 | 6.8×10^-15^ |
| rs4919687 | 10q24.32 | 10 | 104595248 | *WBP1L* | 1.14 | 1.10-1.19 | 7.8×10^-12^ |
| rs12246635 | 10q25.2 | 10 | 114288619 | *VTI1A* | 1.15 | 1.07-1.14 | 4.9×10^-12^ |
| rs1665650 | 10q26.12 | 10 | 118487100 | *HSPA12A* |  |  | 8.6×10^-7^ |
| rs11200014 | 10q26.13 | 10 | 123334930 | *FGFR2* |  |  | 8.0×10^-35^ |
| rs174533 | 11q12.2 | 11 | 61549025 | *MYRF* | 1.15 | 1.05-1.09 | 1.2×10^-11^ |
| rs7121958 | 11q13.4 | 11 | 74280012 |  |  | 1.07-1.10 | 1.4×10^-20^ |
| rs10849432 | 12p13.31 | 12 | 6385727 |  | 1.14 | 1.09-1.18 | 5.8×10^-10^ |
| rs2250430 | 12p13.31 | 12 | 6421174 | *PLEKHG6* | 1.14 | 1.04-1.09 | 3.3×10^-10^ |
| rs2238126 | 12p13.2 | 12 | 12009741 | *ETV6* | 1.17 | 1.12-1.23 | 2.7×10^-10^ |
| rs2730985 | 12q12 | 12 | 43130624 |  | 1.14 | 1.09-1.18 | 1.2×10^-8^ |
| rs11169552 | 12q13.12 | 12 | 51155663 | *ATF1* | 1.09 | 1.05-1.11 | 1.9×10^-10^ |
| rs4759277 | 12q13.3 | 12 | 57533690 | *LRP1* | 1.05 | 1.04-1.07 | 9.4×10^-9^ |
| rs3184504 | 12q24.12 | 12 | 111884608 | *SH2B3* | 1.07 | 1.05-1.1 | 1.7×10^-8^ |
| rs597808 | 12q24.12 | 12 | 111973358 | *ATXN2* | 1.08 | 1.06-1.10 | 2.6×10^-16^ |
| rs377429877 | 13q13.2 | 13 | 34092164 | *STARD13* | 1.05 | 1.03-1.07 | 3.4×10^-7^ |
| rs17094983 | 14q23.1 | 14 | 59189361 |  | 1.11 | 1.08-1.15 | 4.6×10^-11^ |
| rs11844632 | 14q24.1 | 14 | 69026379 | *RAD51B* |  |  | 3.0×10^-10^ |
| rs16969681 | 15q13.3 | 15 | 32993111 | *SCG5* | 1.11 | 1.08-1.14 | 1.4×10^-13^ |
| rs73376930 | 15q13.3 | 15 | 33012502 | *RP11-758N13.1* | 1.14 | 1.1-1.18 | 4.0×10^-18^ |
| rs10152518 | 15q23 | 15 | 68177162 |  | 1.08 | 1.05-1.11 | 3.2×10^-8^ |
| rs16973225 | 15q25.2 | 15 | 82229999 |  |  |  | 8.0×10^-9^ |
| rs7495132 | 15q26.1 | 15 | 91172901 | *CRTC3* | 1.09 | 1.06-1.12 | 7.9×10^-10^ |
| rs9929218 | 16q22.1 | 16 | 68820946 | *CDH1* | 1.1 | 1.06-1.12 | 1.2×10^-8^ |
| rs61336918 | 16q23.2 | 16 | 80007266 |  |  |  | 4.2×10^-10^ |
| rs12149163 | 16q24.1 | 16 | 86339315 |  | 1.05 | 1.03-1.07 | 4.0×10^-9^ |
| rs62042090 | 16q24.1 | 16 | 86703949 |  | 1.05 | 1.03-1.07 | 4.6×10^-6^ |
| rs4968127 | 17p13.3 | 17 | 809643 | *NXN* | 1.07 | 1.05-1.09 | 1.3×10^-12^ |
| rs73975588 | 17p13.3 | 17 | 816741 | *NXN* |  |  | 8.7×10^-9^ |
| rs1078643 | 17p12 | 17 | 10707241 | *TMEM220-AS1* | 1.13 | 1.09-1.17 | 8.4×10^-13^ |
| rs34797592 | 19p13.11 | 19 | 16417198 |  | 1.09 | 1.06-1.12 | 4.2×10^-10^ |
| rs10411210 | 19q13.11 | 19 | 33532300 | *RHPN2* | 1.15 | 1.10-1.20 | 5.0×10^-9^ |
| rs1963413 | 19q13.2 | 19 | 41871573 | *TMEM91* | 1.05 | 1.03-1.06 | 9.5×10^-7^ |
| rs73068325 | 19q13.43 | 19 | 59079096 | *MZF1-AS1* | 1.07 | 1.04-1.09 | 4.2×10^-8^ |
| rs189583 | 20p12.3 | 20 | 6376457 |  | 1.1 | 1.08-1.12 | 1.2×10^-22^ |
| rs961253 | 20p12.3 | 20 | 6404281 |  | 1.08 | 1.06-1.11 | 2.0×10^-10^ |
| rs6085661 | 20p12.3 | 20 | 6693128 |  |  |  | 4.2×10^-10^ |
| rs28488 | 20p12.3 | 20 | 6762221 | *BMP2* | 1.14 | 1.09-1.18 | 2.6×10^-14^ |
| rs11087784 | 20p12.3 | 20 | 7740976 |  | 1.09 | 1.07-1.12 | 2.7×10^-13^ |
| rs2423279 | 20p12.3 | 20 | 7812350 |  | 1.13 | 1.09-1.17 | 3.0×10^-12^ |
| rs11907546 | 20q11.22 | 20 | 32719797 |  |  |  | 3.0×10^-9^ |
| rs6065668 | 20q13.12 | 20 | 42532821 |  | 1.11 | 1.08-1.15 | 4.5×10^-11^ |
| rs6031311 | 20q13.12 | 20 | 42666475 | *TOX2* | 1.06 | 1.04-1.08 | 6.8×10^-9^ |
| rs6066825 | 20q13.13 | 20 | 47340117 | *PREX1* | 1.07 | 1.06-1.09 | 5.9×10^-15^ |
| rs6091213 | 20q13.13 | 20 | 49384745 |  |  |  | 4.4×10^-10^ |
| rs13831 | 20q13.32 | 20 | 57475191 | *GNAS* | 1.15 | 1.10-1.20 | 2.1×10^-8^ |
| rs1741640 | 20q13.33 | 20 | 60932414 | *LAMA5* | 1.12 | 1.10-1.15 | 1.1×10^-26^ |
| rs2427308 | 20q13.33 | 20 | 60969451 | *CABLES2* | 1.12 | 1.09-1.16 | 2.0×10^-13^ |

Note: Chr, chromosome; EAF, effect allele frequency; OR, odds ratio; CI, confidence interval.

# Table. S6. Probes or primers sequence used in the study.

| Electrophoretic mobility shift assay (5'-3') | | |
| --- | --- | --- |
| rs12794623-C | Forward | GGCCACTCAGTTCTGCCACCGTCAC |
|  | Reverse | GTGACGGTGGCAGAACTGAGTGGCC |
| rs12794623-A | Forward | GGCCACTCAGTTATGCCACCGTCAC |
|  | Reverse | GTGACGGTGGCATAACTGAGTGGCC |
| qRT-PCR (5'-3') | | |
| *GAPDH* | Forward | CCTCCCGCTTCGCTCTCT |
|  | Reverse | TGGCGACGCAAAAGAAGAT |
| *POLA2* | Forward | GGCTCCCACCTTGTCTTTGT |
|  | Reverse | ATCGGAGTAGCTGAAAGGCG |
| *GR* | Forward | CGCACAAGTTGATATTCACTG |
|  | Reverse | AGAAGTCCATCACATCTCCC |

qRT-PCR, Quantitative reverse transcription-polymerase chain reaction

# Table. S7. 211 Newly identified variants associated with early-onset CRC risk in the GECCO cohort.

| SNP | Chr | Position | GWAS loci $\pm$1Mb | Effect allele | Phase 1 | | | | | | Phase 2 | | |
| --- | --- | --- | --- | --- | --- | --- | --- | --- | --- | --- | --- | --- | --- |
|  |  |  |  |  | Rsq | Type | EAF | | OR (95% CI) | *P* |  | BETA (-95% CI) | *P* |
|  |  |  |  |  |  |  | Cases | Controls |  |  |  |  |  |
| rs12137323 | 1 | 222125528 | Yes | A | - | Genotyped | 0.19 | 0.16 | 1.25 (1.15-1.34) | 3.68×10^-6^ |  | -0.60 (0.86-0.32) | 1.42×10^-5^ |
| **rs12794623** | **11** | **65029437** |  | **A** | **0.46** | **Imputed** | **0.13** | **0.11** | **1.33 (1.21-1.45)** | **4.41×10^-6^** |  | -0.72 (1.07-0.37) | **4.76×10^-5^** |
| rs401763 | 6 | 27782528 |  | C | - | Genotyped | 0.11 | 0.10 | 1.44 (1.28-1.60) | 4.71×10^-6^ |  | -1.58 (2.03-1.13) | 7.85×10^-12^ |
| rs401754 | 6 | 27782538 |  | A | 0.88 | Imputed | 0.11 | 0.10 | 1.44 (1.28-1.60) | 4.71×10^-6^ |  | -1.58 (2.03-1.13) | 7.85×10^-12^ |
| rs200483 | 6 | 27774824 |  | A | 0.83 | Imputed | 0.11 | 0.10 | 1.44 (1.28-1.59) | 5.91×10^-6^ |  | -1.58 (2.02-1.12) | 9.48×10^-12^ |
| rs200485 | 6 | 27775697 |  | C | - | Imputed | 0.11 | 0.10 | 1.43 (1.28-1.59) | 5.92×10^-6^ |  | -1.56 (2.01-1.11) | 1.10×10^-11^ |
| rs200482 | 6 | 27773904 |  | A | - | Genotyped | 0.11 | 0.11 | 1.43 (1.27-1.58) | 6.45×10^-6^ |  | -1.54 (1.98-1.08) | 2.08×10^-11^ |
| rs200481 | 6 | 27773832 |  | A | 0.75 | Imputed | 0.11 | 0.10 | 1.43 (1.27-1.59) | 6.65×10^-6^ |  | -1.56 (2.01-1.11) | 1.09×10^-11^ |
| rs10139477 | 14 | 36444620 |  | G | - | Imputed | 0.28 | 0.24 | 1.22 (1.13-1.31) | 6.68×10^-6^ |  | -0.45 (0.7-0.19) | 4.89×10^-4^ |
| rs200484 | 6 | 27775674 |  | G | 0.93 | Genotyped | 0.11 | 0.10 | 1.43 (1.28-1.59) | 6.95×10^-6^ |  | -1.58 (2.03-1.13) | 7.82×10^-12^ |
| rs9368531 | 6 | 27781872 |  | C | 0.60 | Imputed | 0.11 | 0.10 | 1.43 (1.27-1.59) | 7.32×10^-6^ |  | -1.57 (2.02-1.11) | 1.11×10^-11^ |
| rs370155 | 6 | 27782031 |  | G | 0.43 | Genotyped | 0.11 | 0.11 | 1.42 (1.27-1.58) | 7.56×10^-6^ |  | -1.53 (1.98-1.08) | 2.60×10^-11^ |
| rs2747054 | 6 | 27783359 |  | G | 0.98 | Genotyped | 0.11 | 0.10 | 1.43 (1.27-1.59) | 7.81×10^-6^ |  | -1.57 (2.02-1.11) | 1.11×10^-11^ |
| rs7520544 | 1 | 222117198 | Yes | C | - | Genotyped | 0.22 | 0.18 | 1.23 (1.14-1.32) | 9.95×10^-6^ |  | -0.55 (0.81-0.29) | 3.26×10^-5^ |
| rs72740077 | 1 | 222114169 | Yes | C | 0.45 | Imputed | 0.22 | 0.18 | 1.23 (1.14-1.32) | 1.02×10^-5^ |  | -0.56 (0.82-0.3) | 2.26×10^-5^ |
| rs486476 | 11 | 116312926 |  | G | 0.83 | Imputed | 0.28 | 0.24 | 1.21 (1.13-1.30) | 1.17×10^-5^ |  | -0.52 (0.77-0.27) | 4.38×10^-5^ |
| rs17011141 | 1 | 222112634 | Yes | G | 0.52 | Genotyped | 0.23 | 0.19 | 1.22 (1.13-1.31) | 1.19×10^-5^ |  | -0.55 (0.8-0.29) | 2.86×10^-5^ |
| rs12124556 | 1 | 222127560 | Yes | T | - | Imputed | 0.19 | 0.16 | 1.23 (1.14-1.33) | 1.22×10^-5^ |  | -0.56 (0.83-0.29) | 4.40×10^-5^ |
| rs6695584 | 1 | 222161989 | Yes | G | 0.84 | Genotyped | 0.22 | 0.20 | 1.22 (1.13-1.31) | 1.26×10^-5^ |  | -0.54 (0.79-0.28) | 3.55×10^-5^ |
| rs12123928 | 1 | 222121875 | Yes | T | 0.61 | Genotyped | 0.22 | 0.18 | 1.22 (1.13-1.31) | 1.27×10^-5^ |  | -0.58 (0.83-0.31) | 1.34×10^-5^ |
| rs200996 | 6 | 27811828 |  | A | 0.91 | Genotyped | 0.10 | 0.10 | 1.42 (1.26-1.57) | 1.40×10^-5^ |  | -1.54 (1.99-1.08) | 2.89×10^-11^ |
| rs200995 | 6 | 27813694 |  | C | - | Genotyped | 0.10 | 0.10 | 1.42 (1.26-1.58) | 1.43×10^-5^ |  | -1.55 (2-1.09) | 2.34×10^-11^ |
| rs200501 | 6 | 27788942 |  | T | 0.74 | Genotyped | 0.11 | 0.10 | 1.41 (1.25-1.57) | 1.55×10^-5^ |  | -1.47 (1.92-1.02) | 1.48×10^-10^ |
| rs17011146 | 1 | 222113648 | Yes | G | 0.97 | Genotyped | 0.22 | 0.19 | 1.22 (1.13-1.31) | 1.58×10^-5^ |  | -0.56 (0.82-0.3) | 1.84×10^-5^ |
| rs12033415 | 1 | 222155068 | Yes | C | 0.83 | Genotyped | 0.22 | 0.20 | 1.22 (1.13-1.30) | 1.60×10^-5^ |  | -0.56 (0.81-0.3) | 2.00×10^-5^ |
| rs6691195 | 1 | 222162373 | Yes | A | - | Genotyped | 0.22 | 0.20 | 1.22 (1.13-1.31) | 1.65×10^-5^ |  | -0.55 (0.8-0.28) | 3.03×10^-5^ |
| rs175597 | 6 | 27810626 |  | C | 0.53 | Imputed | 0.10 | 0.10 | 1.42 (1.26-1.57) | 1.66×10^-5^ |  | -1.57 (2.02-1.11) | 1.40×10^-11^ |
| rs200948 | 6 | 27835272 |  | C | 0.85 | Genotyped | 0.10 | 0.10 | 1.41 (1.25-1.57) | 1.73×10^-5^ |  | -1.53 (1.98-1.07) | 4.11×10^-11^ |
| rs200989 | 6 | 27816442 |  | G | 0.44 | Genotyped | 0.10 | 0.10 | 1.41 (1.26-1.57) | 1.74×10^-5^ |  | -1.56 (2.01-1) | 2.29×10^-11^ |
| rs34295134 | 6 | 27828151 |  | A | 0.48 | Imputed | 0.10 | 0.10 | 1.41 (1.25-1.57) | 1.75×10^-5^ |  | -1.53 (1.98-1.07) | 3.86×10^-11^ |
| rs200983 | 6 | 27830326 |  | T | 0.86 | Imputed | 0.10 | 0.10 | 1.41 (1.25-1.57) | 1.75×10^-5^ |  | -1.53 (1.98-1.07) | 3.86×10^-11^ |
| rs200954 | 6 | 27838764 |  | G | 0.58 | Imputed | 0.10 | 0.10 | 1.41 (1.25-1.57) | 2.04×10^-5^ |  | -1.51 (1.96-1.05) | 7.03×10^-11^ |
| rs200950 | 6 | 27835772 |  | G | - | Imputed | 0.10 | 0.10 | 1.41 (1.25-1.57) | 2.08×10^-5^ |  | -1.51 (1.96-1.05) | 7.03×10^-11^ |
| rs200952 | 6 | 27836976 |  | T | 0.54 | Imputed | 0.10 | 0.10 | 1.41 (1.25-1.57) | 2.08×10^-5^ |  | -1.51 (1.96-1.05) | 7.03×10^-11^ |
| rs200981 | 6 | 27833174 |  | G | - | Imputed | 0.10 | 0.11 | 1.40 (1.25-1.56) | 2.10×10^-5^ |  | -1.48 (1.92-1.02) | 1.45×10^-10^ |
| rs17078858 | 13 | 85537208 |  | T | 0.85 | Imputed | 0.25 | 0.21 | 1.21 (1.12-1.30) | 2.14×10^-5^ |  | -0.48 (0.73-0.22) | 2.09×10^-4^ |
| rs200489 | 6 | 27798257 |  | C | 0.91 | Imputed | 0.10 | 0.10 | 1.41 (1.25-1.57) | 2.35×10^-5^ |  | -1.57 (2.02-1.11) | 1.67×10^-11^ |
| rs201002 | 6 | 27808192 |  | G | 0.59 | Genotyped | 0.10 | 0.10 | 1.40 (1.24-1.56) | 2.36×10^-5^ |  | -1.52 (1.97-1.07) | 4.51×10^-11^ |
| rs7582080 | 2 | 76839833 |  | A | - | Genotyped | 0.28 | 0.24 | 1.20 (1.11-1.28) | 2.42×10^-5^ |  | -0.45 (0.69-0.2) | 3.17×10^-4^ |
| rs949618 | 1 | 222113940 | Yes | C | 0.50 | Genotyped | 0.23 | 0.20 | 1.21 (1.12-1.30) | 2.47×10^-5^ |  | -0.53 (0.78-0.27) | 4.36×10^-5^ |
| rs200497 | 6 | 27792640 |  | T | - | Imputed | 0.10 | 0.10 | 1.40 (1.25-1.56) | 2.53×10^-5^ |  | -1.53 (1.98-1.07) | 4.11×10^-11^ |
| rs138234416 | 6 | 27992898 |  | A | 0.41 | Imputed | 0.09 | 0.08 | 1.50 (1.31-1.7) | 3.07×10^-5^ |  | -2.01 (2.55-1.46) | 4.06×10^-13^ |
| rs200992 | 6 | 27814677 |  | G | 0.98 | Imputed | 0.10 | 0.10 | 1.40 (1.24-1.56) | 3.12×10^-5^ |  | -1.56 (2.01-1.1) | 1.81×10^-11^ |
| rs200990 | 6 | 27815823 |  | G | 0.96 | Genotyped | 0.10 | 0.10 | 1.39 (1.24-1.55) | 3.13×10^-5^ |  | -1.5 (1.95-1.04) | 8.39×10^-11^ |
| rs1620412 | 15 | 42308065 |  | G | 0.89 | Imputed | 0.27 | 0.23 | 1.20 (1.11-1.28) | 3.30×10^-5^ |  | -0.51 (0.75-0.26) | 5.31×10^-5^ |
| rs200490 | 6 | 27796935 |  | T | - | Genotyped | 0.10 | 0.10 | 1.39 (1.24-1.55) | 3.46×10^-5^ |  | -1.52 (1.97-1.06) | 5.11×10^-11^ |
| rs818176 | 2 | 10625156 |  | C | 0.47 | Imputed | 0.17 | 0.17 | 1.21 (1.12-1.29) | 3.61×10^-5^ |  | -0.47 (0.72-0.21) | 3.57×10^-4^ |
| rs12140604 | 1 | 222159150 | Yes | G | 0.82 | Imputed | 0.22 | 0.20 | 1.21 (1.12-1.3) | 3.73×10^-5^ |  | -0.52 (0.77-0.26) | 6.09×10^-5^ |
| rs200977 | 6 | 27854301 |  | C | 0.52 | Imputed | 0.10 | 0.10 | 1.40 (1.24-1.56) | 3.98×10^-5^ |  | -1.54 (2-1.07) | 9.14×10^-11^ |
| rs17600200 | 8 | 41455004 |  | G | - | Genotyped | 0.05 | 0.04 | 1.38 (1.22-1.53) | 4.43×10^-5^ |  | -1.05 (1.54-0.54) | 4.52×10^-5^ |
| rs200953 | 6 | 27837267 |  | C | 0.46 | Genotyped | 0.10 | 0.11 | 1.38 (1.23-1.54) | 4.44×10^-5^ |  | -1.43 (1.88-0.98) | 4.55×10^-10^ |
| rs493161 | 6 | 27850714 |  | T | - | Imputed | 0.10 | 0.10 | 1.40 (1.24-1.56) | 4.58×10^-5^ |  | -1.5 (1.96-1.03) | 2.39×10^-10^ |
| rs557042 | 6 | 27845129 |  | T | 0.88 | Imputed | 0.10 | 0.10 | 1.40 (1.23-1.56) | 4.71×10^-5^ |  | -1.49 (1.95-1.02) | 3.35×10^-10^ |
| rs7071 | 3 | 122293773 |  | T | 0.83 | Imputed | 0.16 | 0.12 | 1.21 (1.12-1.30) | 4.72×10^-5^ |  | -0.57 (0.84-0.28) | 9.89×10^-5^ |
| rs71537572 | 6 | 27970715 |  | C | - | Imputed | 0.08 | 0.08 | 1.50 (1.30-1.69) | 4.82×10^-5^ |  | -1.96 (2.51-1.41) | 2.37×10^-12^ |
| rs1874629 | 16 | 84960383 |  | A | - | Imputed | 0.40 | 0.34 | 1.17 (1.10-1.25) | 4.90×10^-5^ |  | -0.49 (0.7-0.26) | 1.41×10^-5^ |
| rs1996878 | 4 | 189600503 |  | A | 0.75 | Imputed | 0.03 | 0.03 | 1.41 (1.24-1.58) | 5.36×10^-5^ |  | -1.02 (1.55-0.47) | 2.44×10^-4^ |
| rs71559067 | 6 | 27994416 |  | T | - | Imputed | 0.08 | 0.08 | 1.49 (1.30-1.69) | 5.67×10^-5^ |  | -1.98 (2.52-1.42) | 1.93×10^-12^ |
| rs13193295 | 6 | 28003228 |  | G | 0.93 | Imputed | 0.08 | 0.08 | 1.49 (1.30-1.69) | 5.67×10^-5^ |  | -1.98 (2.52-1.42) | 1.93×10^-12^ |
| rs17092631 | 11 | 116476765 |  | C | 0.60 | Genotyped | 0.10 | 0.07 | 1.32 (1.19-1.46) | 6.06×10^-5^ |  | -0.98 (1.4-0.55) | 6.43×10^-6^ |
| rs34203164 | 20 | 38956548 |  | T | 0.43 | Imputed | 0.09 | 0.08 | 1.29 (1.17-1.42) | 6.48×10^-5^ |  | -0.69 (1.06-0.3) | 3.64×10^-4^ |
| rs36101351 | 6 | 27943369 |  | T | 0.98 | Imputed | 0.08 | 0.08 | 1.49 (1.29-1.68) | 6.65×10^-5^ |  | -1.95 (2.5-1.4) | 3.12×10^-12^ |
| rs28360499 | 6 | 27945396 |  | A | - | Imputed | 0.08 | 0.08 | 1.49 (1.29-1.68) | 6.65×10^-5^ |  | -1.95 (2.5-1.4) | 3.12×10^-12^ |
| rs13216117 | 6 | 27938484 |  | G | 0.45 | Imputed | 0.08 | 0.08 | 1.49 (1.29-1.68) | 6.77×10^-5^ |  | -1.95 (2.5-1.4) | 3.12×10^-12^ |
| rs62263868 | 3 | 122295798 |  | G | 0.83 | Imputed | 0.15 | 0.12 | 1.21 (1.12-1.31) | 6.89×10^-5^ |  | -0.55 (0.84-0.26) | 1.97×10^-4^ |
| rs9593961 | 13 | 85534186 |  | T | 0.52 | Imputed | 0.23 | 0.20 | 1.20 (1.11-1.29) | 8.16×10^-5^ |  | -0.48 (0.73-0.21) | 3.00×10^-4^ |
| rs4516110 | 13 | 85534237 |  | C | - | Imputed | 0.23 | 0.20 | 1.20 (1.11-1.28) | 8.78×10^-5^ |  | -0.48 (0.73-0.22) | 2.90×10^-4^ |
| rs9602613 | 13 | 85534477 |  | C | 0.84 | Imputed | 0.23 | 0.20 | 1.20 (1.11-1.28) | 8.81×10^-5^ |  | -0.47 (0.73-0.21) | 3.64×10^-4^ |
| rs9602612 | 13 | 85534254 |  | T | 0.61 | Imputed | 0.23 | 0.20 | 1.20 (1.11-1.28) | 8.83×10^-5^ |  | -0.48 (0.73-0.21) | 3.01×10^-4^ |
| rs9593962 | 13 | 85534434 |  | A | 0.91 | Imputed | 0.23 | 0.20 | 1.20 (1.11-1.28) | 9.22×10^-5^ |  | -0.48 (0.73-0.21) | 3.26×10^-4^ |
| rs9602615 | 13 | 85535626 |  | A | - | Imputed | 0.24 | 0.20 | 1.19 (1.11-1.28) | 9.41×10^-5^ |  | -0.48 (0.73-0.21) | 3.06×10^-4^ |
| rs78893389 | 11 | 116475411 |  | T | 0.74 | Imputed | 0.12 | 0.09 | 1.29 (1.16-1.41) | 9.72×10^-5^ |  | -0.94 (1.33-0.54) | 3.37×10^-6^ |
| rs9593963 | 13 | 85534519 |  | A | 0.97 | Imputed | 0.23 | 0.20 | 1.19 (1.10-1.28) | 1.04×10^-4^ |  | -0.47 (0.72-0.21) | 3.89×10^-4^ |
| rs34583951 | 20 | 38961391 |  | T | 0.83 | Imputed | 0.09 | 0.08 | 1.28 (1.16-1.41) | 1.09×10^-4^ |  | -0.71 (1.09-0.33) | 2.41×10^-4^ |
| rs10871212 | 13 | 85535118 |  | A | - | Imputed | 0.23 | 0.20 | 1.19 (1.10-1.28) | 1.13×10^-4^ |  | -0.47 (0.73-0.21) | 3.51×10^-4^ |
| rs10848143 | 12 | 131025190 |  | G | 0.53 | Imputed | 0.21 | 0.19 | 1.19 (1.10-1.27) | 1.21×10^-4^ |  | -0.55 (0.79-0.29) | 2.13×10^-5^ |
| rs9602614 | 13 | 85535403 |  | G | 0.85 | Imputed | 0.23 | 0.20 | 1.19 (1.10-1.28) | 1.26×10^-4^ |  | -0.48 (0.73-0.21) | 3.03×10^-4^ |
| rs9672119 | 14 | 62961239 |  | T | 0.44 | Imputed | 0.09 | 0.09 | 1.27 (1.15-1.39) | 1.32×10^-4^ |  | -0.84 (1.21-0.47) | 9.26×10^-6^ |
| rs71422968 | 14 | 62964046 |  | T | 0.48 | Imputed | 0.10 | 0.09 | 1.27 (1.15-1.39) | 1.32×10^-4^ |  | -0.82 (1.19-0.45) | 1.55×10^-5^ |
| rs17043770 | 2 | 53132960 |  | G | 0.86 | Genotyped | 0.09 | 0.07 | 1.32 (1.18-1.46) | 1.34×10^-4^ |  | -0.87 (1.29-0.44) | 6.44×10^-5^ |
| rs138088344 | 2 | 53135287 |  | A | 0.58 | Imputed | 0.09 | 0.07 | 1.31 (1.17-1.45) | 1.35×10^-4^ |  | -0.89 (1.31-0.46) | 3.90×10^-5^ |
| rs149498972 | 2 | 53135357 |  | A | - | Imputed | 0.09 | 0.07 | 1.31 (1.17-1.45) | 1.37×10^-4^ |  | -0.88 (1.3-0.45) | 4.57×10^-5^ |
| rs9602610 | 13 | 85533819 |  | G | 0.54 | Imputed | 0.23 | 0.20 | 1.19 (1.10-1.28) | 1.40×10^-4^ |  | -0.47 (0.72-0.2) | 4.08×10^-4^ |
| rs483143 | 6 | 27846744 |  | C | - | Imputed | 0.10 | 0.10 | 1.36 (1.20-1.52) | 1.46×10^-4^ |  | -1.43 (1.88-0.96) | 1.16×10^-9^ |
| rs200979 | 6 | 27852357 |  | A | 0.85 | Genotyped | 0.15 | 0.16 | 1.26 (1.14-1.38) | 1.46×10^-4^ |  | -1.1 (1.44-0.74) | 5.80×10^-10^ |
| rs200975 | 6 | 27855625 |  | T | 0.91 | Imputed | 0.15 | 0.16 | 1.26 (1.14-1.38) | 1.48×10^-4^ |  | -1.1 (1.44-0.75) | 5.32×10^-10^ |
| rs200974 | 6 | 27855845 |  | G | 0.59 | Imputed | 0.15 | 0.16 | 1.26 (1.14-1.38) | 1.48×10^-4^ |  | -1.1 (1.44-0.75) | 5.32×10^-10^ |
| rs200968 | 6 | 27859568 |  | C | - | Genotyped | 0.15 | 0.16 | 1.26 (1.14-1.38) | 1.49×10^-4^ |  | -1.1 (1.44-0.75) | 5.32×10^-10^ |
| rs67662114 | 6 | 27932301 |  | A | 0.50 | Imputed | 0.08 | 0.08 | 1.46 (1.27-1.66) | 1.51×10^-4^ |  | -1.94 (2.49-1.39) | 4.85×10^-12^ |
| rs67380965 | 14 | 62967488 |  | A | - | Imputed | 0.10 | 0.09 | 1.27 (1.15-1.39) | 1.57×10^-4^ |  | -0.77 (1.14-0.39) | 5.69×10^-5^ |
| rs6687758 | 1 | 222164948 | Yes | G | 0.41 | Genotyped | 0.23 | 0.20 | 1.19 (1.10-1.28) | 1.58×10^-4^ |  | -0.53 (0.79-0.27) | 5.17×10^-5^ |
| rs200965 | 6 | 27866384 |  | A | 0.98 | Imputed | 0.15 | 0.16 | 1.26 (1.14-1.38) | 1.59×10^-4^ |  | -1.12 (1.47-0.77) | 2.38×10^-10^ |
| rs1450884 | 7 | 131844887 |  | C | 0.96 | Imputed | 0.31 | 0.28 | 1.18 (1.09-1.26) | 1.61×10^-4^ |  | -0.49 (0.73-0.24) | 7.33×10^-5^ |
| rs67566584 | 20 | 38909950 |  | A | 0.89 | Imputed | 0.09 | 0.08 | 1.28 (1.15-1.40) | 1.67×10^-4^ |  | -0.71 (1.08-0.32) | 2.47×10^-4^ |
| rs494887 | 6 | 27850873 |  | A | - | Imputed | 0.15 | 0.16 | 1.26 (1.14-1.38) | 1.72×10^-4^ |  | -1.09 (1.43-0.74) | 8.12×10^-10^ |
| rs13195728 | 6 | 27771106 |  | C | 0.47 | Imputed | 0.08 | 0.08 | 1.47 (1.27-1.66) | 1.76×10^-4^ |  | -2 (2.55-1.44) | 1.42×10^-12^ |
| rs34115829 | 20 | 38909572 |  | G | 0.82 | Imputed | 0.09 | 0.08 | 1.28 (1.15-1.40) | 1.77×10^-4^ |  | -0.7 (1.08-0.32) | 2.81×10^-4^ |
| rs17011182 | 1 | 222164327 | Yes | G | 0.52 | Genotyped | 0.23 | 0.20 | 1.19 (1.10-1.28) | 1.81×10^-4^ |  | -0.53 (0.78-0.27) | 5.13×10^-5^ |
| rs200949 | 6 | 27835435 |  | G | - | Imputed | 0.11 | 0.11 | 1.33 (1.18-1.48) | 1.82×10^-4^ |  | -1.41 (1.83-0.97) | 1.47×10^-10^ |
| rs9672109 | 14 | 62961049 |  | T | 0.46 | Imputed | 0.10 | 0.09 | 1.26 (1.14-1.38) | 1.86×10^-4^ |  | -0.81 (1.18-0.44) | 1.82×10^-5^ |
| rs200966 | 6 | 27862152 |  | C | - | Imputed | 0.15 | 0.16 | 1.25 (1.14-1.37) | 1.90×10^-4^ |  | -1.08 (1.42-0.72) | 1.12×10^-9^ |
| rs169287 | 6 | 27854760 |  | A | 0.88 | Imputed | 0.15 | 0.16 | 1.25 (1.14-1.37) | 1.92×10^-4^ |  | -1.08 (1.42-0.73) | 1.08×10^-9^ |
| rs200973 | 6 | 27858421 |  | G | 0.83 | Imputed | 0.15 | 0.16 | 1.25 (1.14-1.37) | 1.93×10^-4^ |  | -1.08 (1.42-0.73) | 1.08×10^-9^ |
| rs200976 | 6 | 27854963 |  | G | - | Imputed | 0.15 | 0.16 | 1.25 (1.13-1.37) | 1.95×10^-4^ |  | -1.08 (1.42-0.73) | 1.08×10^-9^ |
| rs34745074 | 14 | 62962210 |  | G | - | Imputed | 0.13 | 0.12 | 1.23 (1.12-1.33) | 1.98×10^-4^ |  | -0.65 (0.97-0.32) | 7.96×10^-5^ |
| rs10505506 | 8 | 129045291 | Yes | G | 0.75 | Genotyped | 0.47 | 0.44 | 1.16 (1.08-1.23) | 2.01×10^-4^ |  | -0.38 (0.59-0.16) | 4.52×10^-4^ |
| rs11594481 | 10 | 133019289 |  | G | - | Imputed | 0.08 | 0.07 | 1.28 (1.15-1.41) | 2.20×10^-4^ |  | -0.73 (1.11-0.33) | 2.76×10^-4^ |
| rs13197633 | 6 | 28174757 |  | A | 0.93 | Imputed | 0.08 | 0.07 | 1.48 (1.27-1.69) | 2.23×10^-4^ |  | -2.23 (2.8-1.64) | 5.86×10^-14^ |
| rs72710971 | 8 | 127091691 |  | T | 0.60 | Imputed | 0.03 | 0.02 | 1.48 (1.27-1.69) | 2.23×10^-4^ |  | -1.21 (1.87-0.54) | 3.51×10^-4^ |
| rs967699 | 8 | 144646890 |  | G | 0.43 | Imputed | 0.30 | 0.27 | 1.18 (1.09-1.27) | 2.39×10^-4^ |  | -0.54 (0.77-0.29) | 1.44×10^-5^ |
| rs13195291 | 6 | 28169241 |  | A | 0.98 | Imputed | 0.08 | 0.07 | 1.48 (1.27-1.69) | 2.42×10^-4^ |  | -2.21 (2.79-1.63) | 7.60×10^-14^ |
| rs17751184 | 6 | 27775028 |  | T | - | Genotyped | 0.08 | 0.08 | 1.46 (1.26-1.66) | 2.43×10^-4^ |  | -1.99 (2.54-1.43) | 2.50×10^-12^ |
| rs9649572 | 7 | 131842842 |  | T | 0.45 | Imputed | 0.31 | 0.28 | 1.17 (1.09-1.25) | 2.44×10^-4^ |  | -0.48 (0.71-0.23) | 1.14×10^-4^ |
| rs818154 | 2 | 10643637 |  | A | 0.83 | Imputed | 0.41 | 0.40 | 1.15 (1.08-1.23) | 2.45×10^-4^ |  | -0.44 (0.65-0.22) | 4.84×10^-5^ |
| rs68190156 | 10 | 133018822 |  | G | 0.52 | Imputed | 0.08 | 0.07 | 1.28 (1.15-1.41) | 2.45×10^-4^ |  | -0.72 (1.11-0.32) | 3.05×10^-4^ |
| rs200964 | 6 | 27866943 |  | C | - | Imputed | 0.15 | 0.16 | 1.25 (1.13-1.37) | 2.48×10^-4^ |  | -1.09 (1.44-0.74) | 6.00×10^-10^ |
| rs34409918 | 6 | 27685348 |  | G | 0.84 | Imputed | 0.08 | 0.07 | 1.46 (1.25-1.66) | 2.48×10^-4^ |  | -1.98 (2.53-1.42) | 3.59×10^-12^ |
| rs9649039 | 7 | 131845112 |  | T | 0.61 | Genotyped | 0.31 | 0.28 | 1.17 (1.09-1.26) | 2.52×10^-4^ |  | -0.48 (0.72-0.24) | 8.80×10^-5^ |
| rs11761595 | 7 | 131846030 |  | A | 0.91 | Imputed | 0.31 | 0.28 | 1.17 (1.09-1.25) | 2.52×10^-4^ |  | -0.46 (0.69-0.21) | 2.17×10^-4^ |
| rs34432857 | 6 | 27685825 |  | A | - | Imputed | 0.08 | 0.07 | 1.45 (1.25-1.65) | 2.52×10^-4^ |  | -1.98 (2.53-1.42) | 3.46×10^-12^ |
| rs12896306 | 14 | 62959638 |  | G | 0.74 | Imputed | 0.13 | 0.12 | 1.22 (1.11-1.33) | 2.57×10^-4^ |  | -0.62 (0.93-0.29) | 1.68×10^-4^ |
| rs34832585 | 6 | 28150878 |  | T | 0.97 | Imputed | 0.08 | 0.07 | 1.48 (1.27-1.68) | 2.59×10^-4^ |  | -2.23 (2.81-1.65) | 4.71×10^-14^ |
| rs13217984 | 6 | 28139710 |  | T | 0.83 | Imputed | 0.08 | 0.07 | 1.48 (1.27-1.68) | 2.61×10^-4^ |  | -2.23 (2.8-1.64) | 5.73×10^-14^ |
| rs67297533 | 6 | 28141253 |  | T | - | Imputed | 0.08 | 0.07 | 1.48 (1.27-1.68) | 2.61×10^-4^ |  | -2.23 (2.8-1.64) | 5.73×10^-14^ |
| rs35781323 | 6 | 28144832 |  | C | 0.53 | Imputed | 0.08 | 0.07 | 1.48 (1.27-1.68) | 2.61×10^-4^ |  | -2.23 (2.8-1.64) | 5.73×10^-14^ |
| rs1874628 | 16 | 84960304 |  | T | 0.85 | Genotyped | 0.38 | 0.33 | 1.16 (1.08-1.23) | 2.62×10^-4^ |  | -0.44 (0.66-0.21) | 1.15×10^-4^ |
| rs72841331 | 10 | 133020295 |  | A | 0.44 | Imputed | 0.08 | 0.08 | 1.28 (1.15-1.41) | 2.65×10^-4^ |  | -0.73 (1.12-0.34) | 1.99×10^-4^ |
| rs12896230 | 14 | 62959334 |  | A | 0.48 | Imputed | 0.13 | 0.12 | 1.22 (1.11-1.33) | 2.69×10^-4^ |  | -0.62 (0.93-0.29) | 1.63×10^-4^ |
| rs13194781 | 6 | 27815639 |  | G | 0.86 | Genotyped | 0.08 | 0.08 | 1.45 (1.25-1.65) | 2.69×10^-4^ |  | -1.98 (2.53-1.42) | 2.64×10^-12^ |
| rs36116761 | 6 | 27818482 |  | G | 0.58 | Imputed | 0.08 | 0.08 | 1.45 (1.25-1.65) | 2.69×10^-4^ |  | -1.98 (2.53-1.42) | 2.64×10^-12^ |
| rs34194357 | 6 | 27818535 |  | G | - | Imputed | 0.08 | 0.08 | 1.45 (1.25-1.65) | 2.69×10^-4^ |  | -1.98 (2.53-1.42) | 2.64×10^-12^ |
| rs13199772 | 6 | 27834085 |  | G | 0.54 | Genotyped | 0.08 | 0.08 | 1.45 (1.25-1.65) | 2.72×10^-4^ |  | -1.99 (2.54-1.42) | 2.63×10^-12^ |
| rs13199906 | 6 | 27834139 |  | G | - | Imputed | 0.08 | 0.08 | 1.45 (1.25-1.65) | 2.72×10^-4^ |  | -1.99 (2.54-1.42) | 2.63×10^-12^ |
| rs13201308 | 6 | 28130089 |  | T | 0.85 | Imputed | 0.08 | 0.07 | 1.47 (1.26-1.68) | 2.75×10^-4^ |  | -2.23 (2.8-1.64) | 5.73×10^-14^ |
| rs34765154 | 6 | 28130450 |  | A | 0.91 | Imputed | 0.08 | 0.07 | 1.47 (1.26-1.68) | 2.75×10^-4^ |  | -2.23 (2.8-1.64) | 5.73×10^-14^ |
| rs34505829 | 6 | 28133239 |  | T | 0.59 | Imputed | 0.08 | 0.07 | 1.47 (1.26-1.68) | 2.75×10^-4^ |  | -2.23 (2.8-1.64) | 5.73×10^-14^ |
| rs35098436 | 6 | 28134221 |  | C | - | Imputed | 0.08 | 0.07 | 1.47 (1.26-1.68) | 2.75×10^-4^ |  | -2.23 (2.8-1.64) | 5.73×10^-14^ |
| rs71559050 | 6 | 27797416 |  | A | 0.50 | Imputed | 0.08 | 0.08 | 1.45 (1.25-1.65) | 2.78×10^-4^ |  | -1.98 (2.54-1.42) | 2.60×10^-12^ |
| rs17763089 | 6 | 27835218 |  | A | - | Imputed | 0.08 | 0.08 | 1.45 (1.25-1.65) | 2.79×10^-4^ |  | -1.99 (2.54-1.42) | 2.63×10^-12^ |
| rs13212318 | 6 | 27688841 |  | C | 0.41 | Imputed | 0.08 | 0.08 | 1.45 (1.25-1.65) | 2.80×10^-4^ |  | -1.95 (2.5-1.39) | 6.10×10^-12^ |
| rs13197175 | 6 | 28129231 |  | T | 0.98 | Genotyped | 0.08 | 0.07 | 1.47 (1.26-1.68) | 2.82×10^-4^ |  | -2.23 (2.8-1.64) | 5.35×10^-14^ |
| rs13197176 | 6 | 28129232 |  | T | 0.96 | Imputed | 0.08 | 0.07 | 1.47 (1.26-1.68) | 2.82×10^-4^ |  | -2.23 (2.8-1.64) | 5.35×10^-14^ |
| rs35819751 | 6 | 27810569 |  | G | 0.89 | Imputed | 0.08 | 0.08 | 1.45 (1.25-1.65) | 2.82×10^-4^ |  | -1.98 (2.54-1.42) | 2.60×10^-12^ |
| rs818174 | 2 | 10626376 |  | T | - | Imputed | 0.38 | 0.35 | 1.15 (1.08-1.23) | 2.83×10^-4^ |  | -0.41 (0.62-0.19) | 2.36×10^-4^ |
| rs17695758 | 6 | 27837183 |  | C | 0.47 | Imputed | 0.08 | 0.08 | 1.45 (1.25-1.65) | 2.86×10^-4^ |  | -1.99 (2.54-1.42) | 2.63×10^-12^ |
| rs34706883 | 6 | 27805255 |  | C | 0.82 | Genotyped | 0.08 | 0.08 | 1.45 (1.25-1.65) | 2.88×10^-4^ |  | -1.98 (2.54-1.42) | 2.60×10^-12^ |
| rs72724802 | 14 | 62958819 |  | C | 0.52 | Imputed | 0.13 | 0.12 | 1.22 (1.11-1.33) | 2.90×10^-4^ |  | -0.63 (0.94-0.3) | 1.30×10^-4^ |
| rs13202291 | 6 | 27698857 |  | G | - | Imputed | 0.08 | 0.08 | 1.45 (1.25-1.65) | 2.93×10^-4^ |  | -1.95 (2.5-1.39) | 6.47×10^-12^ |
| rs10218752 | 1 | 163171868 |  | C | 0.46 | Genotyped | 0.20 | 0.19 | 1.18 (1.09-1.27) | 2.93×10^-4^ |  | -0.48 (0.73-0.21) | 3.59×10^-4^ |
| rs72726503 | 14 | 62959014 |  | C | - | Imputed | 0.13 | 0.12 | 1.22 (1.11-1.33) | 2.94×10^-4^ |  | -0.63 (0.94-0.3) | 1.30×10^-4^ |
| rs72726504 | 14 | 62959068 |  | C | 0.88 | Imputed | 0.13 | 0.12 | 1.22 (1.11-1.33) | 2.94×10^-4^ |  | -0.63 (0.94-0.3) | 1.30×10^-4^ |
| rs35501037 | 6 | 27739566 |  | A | 0.83 | Imputed | 0.08 | 0.08 | 1.44 (1.24-1.64) | 3.02×10^-4^ |  | -1.98 (2.53-1.42) | 2.17×10^-12^ |
| rs34064842 | 6 | 27688625 |  | T | - | Imputed | 0.08 | 0.08 | 1.44 (1.24-1.64) | 3.03×10^-4^ |  | -1.95 (2.49-1.39) | 5.51×10^-12^ |
| rs11848737 | 14 | 62956957 |  | C | - | Imputed | 0.13 | 0.12 | 1.22 (1.11-1.33) | 3.06×10^-4^ |  | -0.63 (0.95-0.31) | 1.15×10^-4^ |
| rs13193480 | 6 | 27702561 |  | G | 0.75 | Imputed | 0.08 | 0.08 | 1.45 (1.25-1.65) | 3.06×10^-4^ |  | -1.95 (2.5-1.39) | 6.06×10^-12^ |
| rs17750424 | 6 | 27701122 |  | C | - | Imputed | 0.08 | 0.08 | 1.45 (1.25-1.65) | 3.06×10^-4^ |  | -1.95 (2.5-1.39) | 6.47×10^-12^ |
| rs13193542 | 6 | 27702425 |  | T | 0.93 | Genotyped | 0.08 | 0.08 | 1.45 (1.25-1.65) | 3.06×10^-4^ |  | -1.95 (2.5-1.39) | 6.47×10^-12^ |
| rs7668968 | 4 | 66726174 |  | G | 0.60 | Imputed | 0.42 | 0.40 | 1.15 (1.07-1.22) | 3.07×10^-4^ |  | -0.44 (0.64-0.22) | 5.64×10^-5^ |
| rs35566684 | 14 | 62958029 |  | A | 0.43 | Imputed | 0.13 | 0.12 | 1.22 (1.11-1.33) | 3.08×10^-4^ |  | -0.63 (0.94-0.3) | 1.30×10^-4^ |
| rs2358662 | 14 | 54807541 | Yes | C | 0.98 | Genotyped | 0.25 | 0.24 | 1.16 (1.08-1.25) | 3.09×10^-4^ |  | -0.46 (0.7-0.22) | 1.35×10^-4^ |
| rs13205911 | 6 | 28124114 |  | T | - | Genotyped | 0.08 | 0.07 | 1.47 (1.26-1.68) | 3.14×10^-4^ |  | -2.2 (2.77-1.61) | 1.17×10^-13^ |
| rs4902139 | 14 | 62957651 |  | C | 0.45 | Genotyped | 0.13 | 0.12 | 1.22 (1.11-1.33) | 3.15×10^-4^ |  | -0.63 (0.94-0.3) | 1.30×10^-4^ |
| rs34662244 | 6 | 28073881 |  | A | 0.83 | Genotyped | 0.08 | 0.07 | 1.47 (1.26-1.67) | 3.18×10^-4^ |  | -2.19 (2.77-1.61) | 1.19×10^-13^ |
| rs35749575 | 6 | 28114818 |  | T | 0.52 | Imputed | 0.08 | 0.07 | 1.47 (1.26-1.67) | 3.18×10^-4^ |  | -2.21 (2.78-1.62) | 9.13×10^-14^ |
| rs72846780 | 6 | 28119055 |  | C | - | Imputed | 0.08 | 0.07 | 1.47 (1.26-1.67) | 3.18×10^-4^ |  | -2.21 (2.78-1.62) | 9.13×10^-14^ |
| rs34718920 | 6 | 27783941 |  | T | 0.84 | Imputed | 0.08 | 0.08 | 1.45 (1.24-1.65) | 3.21×10^-4^ |  | -1.97 (2.52-1.41) | 3.29×10^-12^ |
| rs13197574 | 6 | 28060239 |  | C | 0.61 | Genotyped | 0.08 | 0.07 | 1.47 (1.26-1.67) | 3.29×10^-4^ |  | -2.21 (2.78-1.62) | 8.88×10^-14^ |
| rs35902873 | 6 | 28058949 |  | C | 0.91 | Imputed | 0.08 | 0.07 | 1.47 (1.26-1.67) | 3.29×10^-4^ |  | -2.2 (2.77-1.61) | 1.08×10^-13^ |
| rs34166054 | 6 | 28065801 |  | C | - | Imputed | 0.08 | 0.07 | 1.47 (1.26-1.67) | 3.29×10^-4^ |  | -2.2 (2.77-1.61) | 1.08×10^-13^ |
| rs7493742 | 14 | 62963329 |  | C | 0.74 | Imputed | 0.13 | 0.12 | 1.22 (1.11-1.32) | 3.34×10^-4^ |  | -0.62 (0.93-0.29) | 1.55×10^-4^ |
| rs4899081 | 14 | 62968343 |  | A | 0.97 | Genotyped | 0.13 | 0.12 | 1.22 (1.11-1.33) | 3.34×10^-4^ |  | -0.58 (0.9-0.25) | 4.29×10^-4^ |
| rs67101035 | 6 | 27798887 |  | G | 0.83 | Imputed | 0.08 | 0.08 | 1.44 (1.24-1.64) | 3.34×10^-4^ |  | -1.97 (2.52-1.41) | 3.43×10^-12^ |
| rs13212651 | 6 | 27806985 |  | G | - | Genotyped | 0.08 | 0.08 | 1.44 (1.24-1.64) | 3.34×10^-4^ |  | -1.97 (2.52-1.41) | 3.43×10^-12^ |
| rs818155 | 2 | 10643327 |  | G | 0.53 | Genotyped | 0.41 | 0.40 | 1.15 (1.07-1.22) | 3.36×10^-4^ |  | -0.44 (0.65-0.23) | 4.49×10^-5^ |
| rs34371502 | 6 | 28081758 |  | C | 0.85 | Imputed | 0.08 | 0.07 | 1.46 (1.25-1.67) | 3.37×10^-4^ |  | -2.2 (2.77-1.61) | 1.08×10^-13^ |
| rs13203816 | 6 | 28079598 |  | C | 0.44 | Imputed | 0.08 | 0.07 | 1.46 (1.25-1.67) | 3.37×10^-4^ |  | -2.19 (2.77-1.61) | 1.19×10^-13^ |
| rs34588114 | 6 | 28080628 |  | A | 0.48 | Imputed | 0.08 | 0.07 | 1.46 (1.25-1.67) | 3.37×10^-4^ |  | -2.19 (2.77-1.61) | 1.19×10^-13^ |
| rs68188794 | 6 | 28080777 |  | C | 0.86 | Imputed | 0.08 | 0.07 | 1.46 (1.25-1.67) | 3.37×10^-4^ |  | -2.19 (2.77-1.61) | 1.19×10^-13^ |
| rs10498500 | 14 | 62960508 |  | G | 0.58 | Imputed | 0.13 | 0.12 | 1.22 (1.11-1.32) | 3.38×10^-4^ |  | -0.61 (0.93-0.29) | 1.77×10^-4^ |
| rs34788973 | 6 | 27879200 |  | A | - | Genotyped | 0.08 | 0.07 | 1.45 (1.25-1.65) | 3.42×10^-4^ |  | -2.12 (2.68-1.55) | 2.58×10^-13^ |
| rs61742093 | 6 | 27879982 |  | G | 0.54 | Genotyped | 0.08 | 0.07 | 1.45 (1.25-1.65) | 3.42×10^-4^ |  | -2.11 (2.67-1.54) | 3.08×10^-13^ |
| rs71559072 | 6 | 28089481 |  | T | - | Imputed | 0.08 | 0.07 | 1.46 (1.25-1.67) | 3.42×10^-4^ |  | -2.21 (2.79-1.63) | 7.63×10^-14^ |
| rs66886492 | 6 | 28089731 |  | T | 0.85 | Imputed | 0.08 | 0.07 | 1.46 (1.25-1.67) | 3.42×10^-4^ |  | -2.21 (2.79-1.63) | 7.63×10^-14^ |
| rs35345226 | 6 | 28091580 |  | G | 0.91 | Imputed | 0.08 | 0.07 | 1.46 (1.25-1.67) | 3.42×10^-4^ |  | -2.21 (2.79-1.63) | 7.63×10^-14^ |
| rs72847313 | 6 | 27730082 |  | T | 0.59 | Imputed | 0.08 | 0.08 | 1.44 (1.24-1.64) | 3.47×10^-4^ |  | -1.98 (2.53-1.42) | 2.17×10^-12^ |
| rs17750747 | 6 | 27730334 |  | C | - | Imputed | 0.08 | 0.08 | 1.44 (1.24-1.64) | 3.47×10^-4^ |  | -1.98 (2.53-1.42) | 2.17×10^-12^ |
| rs11625082 | 14 | 62970049 |  | T | 0.50 | Imputed | 0.09 | 0.09 | 1.26 (1.13-1.38) | 3.47×10^-4^ |  | -0.75 (1.13-0.36) | 1.19×10^-4^ |
| rs71559070 | 6 | 28038929 |  | A | - | Imputed | 0.08 | 0.07 | 1.46 (1.25-1.67) | 3.52×10^-4^ |  | -2.19 (2.76-1.6) | 1.33×10^-13^ |
| rs9552210 | 13 | 21067941 |  | G | 0.41 | Imputed | 0.49 | 0.46 | 1.16 (1.08-1.24) | 3.54×10^-4^ |  | -0.4 (0.62-0.17) | 3.81×10^-4^ |
| rs12879281 | 14 | 62965149 |  | G | 0.98 | Imputed | 0.13 | 0.12 | 1.22 (1.11-1.32) | 3.63×10^-4^ |  | -0.59 (0.91-0.27) | 2.93×10^-4^ |
| rs13199649 | 6 | 27868792 |  | T | 0.96 | Genotyped | 0.08 | 0.07 | 1.46 (1.25-1.67) | 3.67×10^-4^ |  | -2.14 (2.71-1.56) | 3.87×10^-13^ |
| rs431845 | 13 | 113421026 |  | C | 0.89 | Imputed | 0.06 | 0.06 | 1.31 (1.16-1.46) | 3.68×10^-4^ |  | -0.83 (1.29-0.36) | 4.33×10^-4^ |
| rs4675566 | 2 | 206787764 |  | C | - | Imputed | 0.26 | 0.24 | 1.17 (1.08-1.26) | 3.69×10^-4^ |  | -0.52 (0.78-0.26) | 5.69×10^-5^ |
| rs2350073 | 4 | 66717214 |  | T | 0.47 | Imputed | 0.42 | 0.41 | 1.15 (1.07-1.22) | 3.81×10^-4^ |  | -0.44 (0.64-0.22) | 5.18×10^-5^ |
| rs35037868 | 6 | 27759115 |  | C | 0.82 | Imputed | 0.08 | 0.07 | 1.45 (1.24-1.65) | 3.86×10^-4^ |  | -1.93 (2.49-1.36) | 2.36×10^-11^ |
| rs4146476 | 4 | 66716573 |  | C | 0.52 | Genotyped | 0.42 | 0.41 | 1.15 (1.07-1.22) | 3.92×10^-4^ |  | -0.44 (0.64-0.22) | 5.41×10^-5^ |
| rs4552913 | 8 | 144647005 |  | G | - | Imputed | 0.30 | 0.27 | 1.17 (1.08-1.26) | 3.93×10^-4^ |  | -0.51 (0.74-0.26) | 4.32×10^-5^ |
| rs28510083 | 4 | 55456806 |  | T | 0.46 | Imputed | 0.22 | 0.20 | 1.17 (1.09-1.26) | 3.99×10^-4^ |  | -0.53 (0.79-0.27) | 4.37×10^-5^ |
| rs73149853 | 7 | 130883780 |  | A | - | Imputed | 0.17 | 0.13 | 1.22 (1.11-1.33) | 4.16×10^-4^ |  | -0.61 (0.94-0.28) | 2.85×10^-4^ |
| rs13218875 | 6 | 27884012 |  | T | 0.88 | Genotyped | 0.08 | 0.07 | 1.45 (1.25-1.66) | 4.21×10^-4^ |  | -2.13 (2.7-1.55) | 4.88×10^-13^ |
| rs16974504 | 15 | 54612426 |  | G | 0.83 | Genotyped | 0.15 | 0.11 | 1.22 (1.11-1.34) | 4.22×10^-4^ |  | -0.66 (1-0.32) | 1.32×10^-4^ |
| rs9509249 | 13 | 21068045 |  | C | - | Imputed | 0.49 | 0.46 | 1.16 (1.07-1.24) | 4.23×10^-4^ |  | -0.4 (0.62-0.18) | 3.71×10^-4^ |
| rs10958911 | 9 | 10135016 |  | A | - | Imputed | 0.13 | 0.11 | 1.21 (1.11-1.32) | 4.26×10^-4^ |  | -0.59 (0.91-0.26) | 3.88×10^-4^ |
| rs56147533 | 14 | 62955966 |  | T | 0.75 | Imputed | 0.13 | 0.12 | 1.21 (1.11-1.32) | 4.35×10^-4^ |  | -0.62 (0.93-0.29) | 1.74×10^-4^ |
| rs13200214 | 6 | 28017250 |  | T | - | Genotyped | 0.08 | 0.07 | 1.45 (1.24-1.66) | 4.36×10^-4^ |  | -2.12 (2.69-1.54) | 6.65×10^-13^ |
| rs28589726 | 4 | 66720239 |  | C | 0.93 | Imputed | 0.42 | 0.41 | 1.14 (1.07-1.22) | 4.38×10^-4^ |  | -0.43 (0.63-0.21) | 7.80×10^-5^ |
| rs71559054 | 6 | 27896799 |  | C | 0.60 | Imputed | 0.08 | 0.07 | 1.45 (1.24-1.66) | 4.42×10^-4^ |  | -2.14 (2.71-1.55) | 4.23×10^-13^ |
| rs2350077 | 4 | 66721398 |  | A | 0.43 | Imputed | 0.42 | 0.40 | 1.14 (1.07-1.22) | 4.44×10^-4^ |  | -0.42 (0.63-0.2) | 1.02×10^-4^ |
| rs4899079 | 14 | 62956327 |  | T | 0.98 | Imputed | 0.13 | 0.12 | 1.21 (1.11-1.32) | 4.53×10^-4^ |  | -0.62 (0.93-0.29) | 1.74×10^-4^ |
| rs187956 | 1 | 77508652 |  | G | - | Imputed | 0.35 | 0.32 | 1.15 (1.07-1.23) | 4.58×10^-4^ |  | -0.45 (0.67-0.22) | 1.05×10^-4^ |
| rs35702572 | 14 | 63087698 |  | C | 0.45 | Imputed | 0.28 | 0.24 | 1.16 (1.08-1.25) | 4.90×10^-4^ |  | -0.48 (0.72-0.23) | 1.16×10^-4^ |

Note: Chr, chromosome; EAF, effect allele frequency; OR, odds ratio; CI, Confidence interval.

Rsq values: reflect imputation quality are obtained from output of Michigan Imputation Server.

# Table. S8. 84 newly identified variants associated with EOCRC risk (under 40 years old) in the GECCO cohort.

| SNP | Chr | Position | Gwas loci $\pm$1Mb | Effect allele | Phase 1 | | | | | | Phase 2 | | |
| --- | --- | --- | --- | --- | --- | --- | --- | --- | --- | --- | --- | --- | --- |
|  |  |  |  |  | Rsq | Type | EAF | | OR (95% CI) | P | | BETA (-95% CI) | P |
|  |  |  |  |  |  |  | Cases | Controls |  |  |  |  |  |
| rs199697 | 1 | 77506130 |  | C | 0.93 | Imputed | 0.25 | 0.20 | 1.69 (1.47-1.90) | 1.69×10^-6^ |  | -0.53 (0.25-0.8) | 1.7×10^-4^ |
| rs199691 | 1 | 77502479 |  | C | 0.94 | Imputed | 0.25 | 0.20 | 1.68 (1.47-1.90) | 1.86×10^-6^ |  | -0.51 (0.23-0.78) | 2.76×10^-4^ |
| rs10218752 | 1 | 163171868 |  | C | - | Genotyped | 0.23 | 0.17 | 1.57 (1.35-1.78) | 3.72×10^-5^ |  | -0.48 (0.22-0.74) | 3.59×10^-4^ |
| rs199719 | 1 | 77525211 |  | T | 0.97 | Imputed | 0.44 | 0.32 | 1.50 (1.30-1.69) | 4.45×10^-5^ |  | -0.42 (0.19-0.65) | 3.89×10^-4^ |
| rs200992 | 6 | 27814677 |  | G | 0.99 | Imputed | 0.12 | 0.10 | 2.25 (1.85-2.64) | 5.83×10^-5^ |  | -1.56 (1.11-2.02) | 1.81×10^-11^ |
| rs200990 | 6 | 27815823 |  | G | 1.00 | Imputed | 0.12 | 0.10 | 2.21 (1.82-2.60) | 7.24×10^-5^ |  | -1.5 (1.05-1.95) | 8.39×10^-11^ |
| rs199666 | 1 | 77535433 |  | A | 0.99 | Imputed | 0.46 | 0.34 | 1.47 (1.28-1.66) | 7.99×10^-5^ |  | -0.4 (0.18-0.63) | 4.48×10^-4^ |
| rs200485 | 6 | 27775697 |  | C | 0.99 | Imputed | 0.12 | 0.10 | 2.20 (1.80-2.59) | 8.70×10^-5^ |  | -1.56 (1.11-2.01) | 1.1×10^-11^ |
| rs200481 | 6 | 27773832 |  | A | 0.99 | Imputed | 0.12 | 0.10 | 2.20 (1.80-2.59) | 8.78×10^-5^ |  | -1.56 (1.11-2.01) | 1.09×10^-11^ |
| rs10917696 | 1 | 163149325 |  | C | 0.88 | Imputed | 0.23 | 0.17 | 1.54 (1.32-1.75) | 8.88×10^-5^ |  | -0.49 (0.23-0.76) | 2.37×10^-4^ |
| rs10917697 | 1 | 163149497 |  | G | 0.88 | Imputed | 0.23 | 0.17 | 1.54 (1.32-1.75) | 8.88×10^-5^ |  | -0.49 (0.23-0.76) | 2.37×10^-4^ |
| rs201002 | 6 | 27808192 |  | G | 1.00 | Imputed | 0.12 | 0.10 | 2.18 (1.79-2.57) | 9.02×10^-5^ |  | -1.52 (1.07-1.97) | 4.51×10^-11^ |
| rs199720 | 1 | 77526482 |  | C | 0.99 | Imputed | 0.46 | 0.34 | 1.46 (1.27-1.65) | 9.26×10^-5^ |  | -0.4 (0.18-0.63) | 4.98×10^-4^ |
| rs200482 | 6 | 27773904 |  | A | 0.99 | Imputed | 0.12 | 0.10 | 2.18 (1.79-2.57) | 9.53×10^-5^ |  | -1.54 (1.09-1.99) | 2.08×10^-11^ |
| rs200981 | 6 | 27833174 |  | G | 1.00 | Imputed | 0.12 | 0.10 | 2.16 (1.77-2.54) | 1.02×10^-4^ |  | -1.48 (1.03-1.93) | 1.45×10^-10^ |
| rs17751184 | 6 | 27775028 |  | T | 0.99 | Imputed | 0.10 | 0.07 | 2.65 (2.16-3.14) | 1.02×10^-4^ |  | -1.99 (1.43-2.54) | 2.5×10^-12^ |
| rs200953 | 6 | 27837267 |  | C | 1.00 | Imputed | 0.12 | 0.10 | 2.15 (1.76-2.54) | 1.09×10^-4^ |  | -1.43 (0.98-1.88) | 4.55×10^-10^ |
| rs73062921 | 5 | 16859464 |  | A | 0.93 | Imputed | 0.15 | 0.09 | 1.68 (1.42-1.95) | 1.1×10^-4^ |  | -0.69 (0.35-1.04) | 8.8×10^-5^ |
| rs13199772 | 6 | 27834085 |  | G | 0.99 | Imputed | 0.10 | 0.07 | 2.62 (2.13-3.11) | 1.12×10^-4^ |  | -1.99 (1.43-2.54) | 2.63×10^-12^ |
| rs13199906 | 6 | 27834139 |  | G | 0.99 | Imputed | 0.10 | 0.07 | 2.62 (2.13-3.11) | 1.12×10^-4^ |  | -1.99 (1.43-2.54) | 2.63×10^-12^ |
| rs17763089 | 6 | 27835218 |  | A | 0.99 | Imputed | 0.10 | 0.07 | 2.62 (2.13-3.11) | 1.12×10^-4^ |  | -1.99 (1.43-2.54) | 2.63×10^-12^ |
| rs17695758 | 6 | 27837183 |  | C | 0.99 | Imputed | 0.10 | 0.07 | 2.62 (2.13-3.11) | 1.12×10^-4^ |  | -1.99 (1.43-2.54) | 2.63×10^-12^ |
| rs9789226 | 18 | 13804919 |  | G | 0.95 | Imputed | 0.24 | 0.14 | 1.57 (1.34-1.79) | 1.12×10^-4^ |  | -0.51 (0.23-0.8) | 4.51×10^-4^ |
| rs370155 | 6 | 27782031 |  | G | 0.99 | Imputed | 0.12 | 0.10 | 2.16 (1.76-2.55) | 1.14×10^-4^ |  | -1.53 (1.08-1.98) | 2.6×10^-11^ |
| rs13194781 | 6 | 27815639 |  | G | 0.99 | Imputed | 0.10 | 0.07 | 2.62 (2.13-3.11) | 1.16×10^-4^ |  | -1.98 (1.43-2.54) | 2.64×10^-12^ |
| rs36116761 | 6 | 27818482 |  | G | 0.99 | Imputed | 0.10 | 0.07 | 2.62 (2.13-3.11) | 1.16×10^-4^ |  | -1.98 (1.43-2.54) | 2.64×10^-12^ |
| rs34194357 | 6 | 27818535 |  | G | 0.99 | Imputed | 0.10 | 0.07 | 2.62 (2.13-3.11) | 1.16×10^-4^ |  | -1.98 (1.43-2.54) | 2.64×10^-12^ |
| rs13195728 | 6 | 27771106 |  | C | 0.99 | Imputed | 0.10 | 0.07 | 2.62 (2.13-3.10) | 1.19×10^-4^ |  | -2 (1.45-2.56) | 1.42×10^-12^ |
| rs200989 | 6 | 27816442 |  | G | 1.00 | Imputed | 0.12 | 0.10 | 2.18 (1.78-2.57) | 1.25×10^-4^ |  | -1.56 (1.1-2.01) | 2.29×10^-11^ |
| rs200954 | 6 | 27838764 |  | G | 1.00 | Imputed | 0.12 | 0.10 | 2.16 (1.77-2.56) | 1.28×10^-4^ |  | -1.51 (1.06-1.96) | 7.03×10^-11^ |
| rs199717 | 1 | 77524318 |  | C | 0.98 | Imputed | 0.46 | 0.34 | 1.45 (1.26-1.64) | 1.3×10^-4^ |  | -0.41 (0.18-0.63) | 4.43×10^-4^ |
| rs199702 | 1 | 77508007 |  | A | 0.94 | Imputed | 0.46 | 0.35 | 1.45 (1.26-1.64) | 1.32×10^-4^ |  | -0.42 (0.19-0.65) | 2.81×10^-4^ |
| rs200995 | 6 | 27813694 |  | C | 1.00 | Imputed | 0.12 | 0.10 | 2.17 (1.77-2.56) | 1.34×10^-4^ |  | -1.55 (1.1-2.01) | 2.34×10^-11^ |
| rs199696 | 1 | 77505413 |  | G | 0.94 | Imputed | 0.46 | 0.35 | 1.45 (1.26-1.64) | 1.35×10^-4^ |  | -0.41 (0.19-0.64) | 3.33×10^-4^ |
| rs71559050 | 6 | 27797416 |  | A | 0.99 | Imputed | 0.10 | 0.07 | 2.58 (2.09-3.06) | 1.37×10^-4^ |  | -1.98 (1.43-2.54) | 2.6×10^-12^ |
| rs35819751 | 6 | 27810569 |  | G | 0.99 | Imputed | 0.10 | 0.07 | 2.58 (2.09-3.06) | 1.37×10^-4^ |  | -1.98 (1.43-2.54) | 2.6×10^-12^ |
| rs34409918 | 6 | 27685348 |  | G | 0.97 | Imputed | 0.10 | 0.07 | 2.57 (2.08-3.06) | 1.37×10^-4^ |  | -1.98 (1.42-2.53) | 3.59×10^-12^ |
| rs34432857 | 6 | 27685825 |  | A | 0.97 | Imputed | 0.10 | 0.07 | 2.57 (2.08-3.06) | 1.37×10^-4^ |  | -1.98 (1.42-2.53) | 3.46×10^-12^ |
| rs13212318 | 6 | 27688841 |  | C | 0.98 | Imputed | 0.10 | 0.07 | 2.57 (2.08-3.06) | 1.37×10^-4^ |  | -1.95 (1.4-2.51) | 6.1×10^-12^ |
| rs13202291 | 6 | 27698857 |  | G | 0.99 | Imputed | 0.10 | 0.07 | 2.57 (2.08-3.06) | 1.37×10^-4^ |  | -1.95 (1.39-2.5) | 6.47×10^-12^ |
| rs17750424 | 6 | 27701122 |  | C | 0.99 | Imputed | 0.10 | 0.07 | 2.57 (2.08-3.06) | 1.37×10^-4^ |  | -1.95 (1.39-2.5) | 6.47×10^-12^ |
| rs13193542 | 6 | 27702425 |  | T | 0.99 | Imputed | 0.10 | 0.07 | 2.57 (2.08-3.06) | 1.37×10^-4^ |  | -1.95 (1.39-2.5) | 6.47×10^-12^ |
| rs13193480 | 6 | 27702561 |  | G | 0.99 | Imputed | 0.10 | 0.07 | 2.57 (2.08-3.06) | 1.37×10^-4^ |  | -1.95 (1.4-2.51) | 6.06×10^-12^ |
| rs34718920 | 6 | 27783941 |  | T | 0.99 | Imputed | 0.10 | 0.07 | 2.58 (2.09-3.06) | 1.38×10^-4^ |  | -1.97 (1.42-2.53) | 3.29×10^-12^ |
| rs2003706 | 1 | 77522015 |  | G | 0.98 | Imputed | 0.46 | 0.34 | 1.45 (1.26-1.64) | 1.38×10^-4^ |  | -0.43 (0.2-0.65) | 2.19×10^-4^ |
| rs187956 | 1 | 77508652 |  | G | 0.94 | Imputed | 0.46 | 0.34 | 1.45 (1.26-1.64) | 1.41×10^-4^ |  | -0.45 (0.22-0.68) | 1.05×10^-4^ |
| rs67101035 | 6 | 27798887 |  | G | 0.99 | Imputed | 0.10 | 0.07 | 2.57 (2.08-3.06) | 1.44×10^-4^ |  | -1.97 (1.42-2.53) | 3.43×10^-12^ |
| rs34706883 | 6 | 27805255 |  | C | 0.99 | Imputed | 0.10 | 0.07 | 2.57 (2.08-3.06) | 1.44×10^-4^ |  | -1.98 (1.43-2.54) | 2.6×10^-12^ |
| rs13212651 | 6 | 27806985 |  | G | 0.99 | Imputed | 0.10 | 0.07 | 2.57 (2.08-3.06) | 1.44×10^-4^ |  | -1.97 (1.42-2.53) | 3.43×10^-12^ |
| rs200996 | 6 | 27811828 |  | A | 1.00 | Imputed | 0.12 | 0.10 | 2.14 (1.75-2.54) | 1.51×10^-4^ |  | -1.54 (1.09-2) | 2.89×10^-11^ |
| rs175597 | 6 | 27810626 |  | C | 1.00 | Imputed | 0.12 | 0.10 | 2.15 (1.75-2.54) | 1.54×10^-4^ |  | -1.57 (1.12-2.03) | 1.4×10^-11^ |
| rs200950 | 6 | 27835772 |  | G | 1.00 | Imputed | 0.12 | 0.10 | 2.13 (1.74-2.52) | 1.6×10^-4^ |  | -1.51 (1.06-1.96) | 7.03×10^-11^ |
| rs200952 | 6 | 27836976 |  | T | 1.00 | Imputed | 0.12 | 0.10 | 2.13 (1.74-2.52) | 1.6×10^-4^ |  | -1.51 (1.06-1.96) | 7.03×10^-11^ |
| rs199699 | 1 | 77507030 |  | C | 0.94 | Imputed | 0.46 | 0.35 | 1.45 (1.26-1.64) | 1.62×10^-4^ |  | -0.41 (0.19-0.64) | 3.68×10^-4^ |
| rs200483 | 6 | 27774824 |  | A | 0.99 | Imputed | 0.12 | 0.10 | 2.14 (1.75-2.54) | 1.63×10^-4^ |  | -1.58 (1.12-2.03) | 9.48×10^-12^ |
| rs200484 | 6 | 27775674 |  | G | 0.99 | Imputed | 0.12 | 0.10 | 2.14 (1.75-2.54) | 1.63×10^-4^ |  | -1.58 (1.13-2.04) | 7.82×10^-12^ |
| rs200948 | 6 | 27835272 |  | C | - | Genotyped | 0.12 | 0.10 | 2.12 (1.73-2.51) | 1.72×10^-4^ |  | -1.53 (1.08-1.98) | 4.11×10^-11^ |
| rs34064842 | 6 | 27688625 |  | T | 0.98 | Imputed | 0.10 | 0.07 | 2.52 (2.03-3.00) | 1.8×10^-4^ |  | -1.95 (1.39-2.5) | 5.51×10^-12^ |
| rs72847313 | 6 | 27730082 |  | T | 0.99 | Imputed | 0.10 | 0.07 | 2.52 (2.03-3.00) | 1.8×10^-4^ |  | -1.98 (1.43-2.53) | 2.17×10^-12^ |
| rs17750747 | 6 | 27730334 |  | C | 0.99 | Imputed | 0.10 | 0.07 | 2.52 (2.03-3.00) | 1.8×10^-4^ |  | -1.98 (1.43-2.53) | 2.17×10^-12^ |
| rs35501037 | 6 | 27739566 |  | A | 0.99 | Imputed | 0.10 | 0.07 | 2.52 (2.03-3.00) | 1.8×10^-4^ |  | -1.98 (1.43-2.53) | 2.17×10^-12^ |
| rs401763 | 6 | 27782528 |  | C | 0.99 | Imputed | 0.12 | 0.10 | 2.12 (1.73-2.52) | 1.85×10^-4^ |  | -1.58 (1.13-2.04) | 7.85×10^-12^ |
| rs401754 | 6 | 27782538 |  | A | 0.99 | Imputed | 0.12 | 0.10 | 2.12 (1.73-2.52) | 1.85×10^-4^ |  | -1.58 (1.13-2.04) | 7.85×10^-12^ |
| rs34295134 | 6 | 27828151 |  | A | 1.00 | Imputed | 0.12 | 0.10 | 2.11 (1.71-2.50) | 1.88×10^-4^ |  | -1.53 (1.08-1.99) | 3.86×10^-11^ |
| rs200983 | 6 | 27830326 |  | T | 1.00 | Imputed | 0.12 | 0.10 | 2.11 (1.71-2.50) | 1.88×10^-4^ |  | -1.53 (1.08-1.99) | 3.86×10^-11^ |
| rs9368531 | 6 | 27781872 |  | C | 0.99 | Imputed | 0.12 | 0.10 | 2.12 (1.72-2.51) | 1.94×10^-4^ |  | -1.57 (1.12-2.03) | 1.11×10^-11^ |
| rs2747054 | 6 | 27783359 |  | G | 1.00 | Imputed | 0.12 | 0.10 | 2.12 (1.72-2.51) | 1.94×10^-4^ |  | -1.57 (1.12-2.03) | 1.11×10^-11^ |
| rs200490 | 6 | 27796935 |  | T | 1.00 | Imputed | 0.12 | 0.10 | 2.11 (1.71-2.50) | 2.05×10^-4^ |  | -1.52 (1.07-1.97) | 5.11×10^-11^ |
| rs13343310 | 19 | 52433467 |  | T | - | Genotyped | 0.36 | 0.30 | 1.46 (1.26-1.67) | 2.16×10^-4^ |  | -0.44 (0.2-0.67) | 3.00×10^-4^ |
| rs10152984 | 15 | 84016917 |  | T | 0.84 | Imputed | 0.46 | 0.39 | 1.42 (1.23-1.61) | 2.35×10^-4^ |  | -0.39 (0.17-0.61) | 4.25×10^-4^ |
| rs11162254 | 1 | 77508917 |  | C | 0.94 | Imputed | 0.45 | 0.34 | 1.43 (1.24-1.63) | 2.43×10^-4^ |  | -0.45 (0.22-0.67) | 1.22×10^-4^ |
| rs199707 | 1 | 77514455 |  | T | 0.95 | Imputed | 0.45 | 0.34 | 1.43 (1.24-1.62) | 2.52×10^-4^ |  | -0.43 (0.2-0.66) | 2.08×10^-4^ |
| rs199673 | 1 | 77546779 |  | T | 0.97 | Imputed | 0.45 | 0.33 | 1.43 (1.24-1.62) | 2.57×10^-4^ |  | -0.41 (0.19-0.64) | 3.58×10^-4^ |
| rs12023405 | 1 | 77508751 |  | G | 0.94 | Imputed | 0.45 | 0.34 | 1.43 (1.24-1.62) | 2.84×10^-4^ |  | -0.44 (0.21-0.67) | 1.64×10^-4^ |
| rs11666384 | 19 | 52437261 |  | A | 0.99 | Imputed | 0.38 | 0.31 | 1.45 (1.25-1.65) | 2.85×10^-4^ |  | -0.45 (0.22-0.69) | 1.6×10^-4^ |
| rs61782867 | 1 | 77511827 |  | A | 0.98 | Imputed | 0.18 | 0.11 | 1.59 (1.34-1.84) | 2.86×10^-4^ |  | -0.65 (0.32-0.99) | 1.27×10^-4^ |
| rs919779 | 19 | 52438347 |  | C | 0.99 | Imputed | 0.38 | 0.31 | 1.44 (1.24-1.64) | 3.34×10^-4^ |  | -0.46 (0.22-0.69) | 1.39×10^-4^ |
| rs200489 | 6 | 27798257 |  | C | 1.00 | Imputed | 0.12 | 0.10 | 2.07 (1.67-2.47) | 3.44×10^-4^ |  | -1.57 (1.11-2.02) | 1.67×10^-11^ |
| rs199704 | 1 | 77517175 |  | C | 0.97 | Imputed | 0.45 | 0.34 | 1.42 (1.22-1.61) | 3.75×10^-4^ |  | -0.42 (0.2-0.65) | 2.66×10^-4^ |
| rs200497 | 6 | 27792640 |  | T | 1.00 | Imputed | 0.12 | 0.10 | 2.05 (1.65-2.45) | 3.83×10^-4^ |  | -1.53 (1.08-1.99) | 4.11×10^-11^ |
| rs200501 | 6 | 27788942 |  | T | 1.00 | Imputed | 0.12 | 0.10 | 2.03 (1.64-2.42) | 3.91×10^-4^ |  | -1.47 (1.02-1.92) | 1.48×10^-10^ |
| rs1233487 | 6 | 29468809 |  | G | 0.92 | Imputed | 0.31 | 0.23 | 1.58 (1.32-1.83) | 4.48×10^-4^ |  | -0.64 (0.34-0.95) | 3.59×10^-5^ |
| rs2914906 | 5 | 117983460 |  | G | 0.96 | Imputed | 0.36 | 0.31 | 1.40 (1.21-1.59) | 4.84×10^-4^ |  | -0.43 (0.2-0.66) | 2.45×10^-4^ |
| rs7250921 | 19 | 52459256 |  | A | 0.99 | Imputed | 0.38 | 0.31 | 1.43 (1.23-1.63) | 4.96×10^-4^ |  | -0.43 (0.2-0.67) | 3.13×10^-4^ |

Note: Chr, chromosome; EAF, effect allele frequency; OR, odds ratio; CI, confidence interval.

Rsq values: reflect imputation quality are obtained from output of Michigan Imputation Server.

# Table. S9. Results for 16 identified variants associated with EOCRC risk (under 40 years old) in the GECCO cohort (LD r^2^ < 0.6).

| SNP | Chr | Position | Gwas loci $\pm$1Mb | Effect allele | Phase 1 | | | | | | Phase 2 | | |
| --- | --- | --- | --- | --- | --- | --- | --- | --- | --- | --- | --- | --- | --- |
|  |  |  |  |  | Rsq | Type | EAF | | OR (95% CI) | P | | BETA (-95% CI) | P |
|  |  |  |  |  |  |  | Cases | Controls |  |  |  |  |  |
| rs199697 | 1 | 77506130 |  | C | 0.93 | Imputed | 0.25 | 0.20 | 1.69 (1.47-1.90) | 1.69×10^-6^ |  | -0.53 (0.25-0.8) | 1.7×10^-4^ |
| rs10218752 | 1 | 163171868 |  | C | - | Genotyped | 0.23 | 0.17 | 1.57 (1.35-1.78) | 3.72×10^-5^ |  | -0.48 (0.22-0.74) | 3.59×10^-4^ |
| rs199719 | 1 | 77525211 |  | T | 0.97 | Imputed | 0.44 | 0.32 | 1.50 (1.30-1.69) | 4.45×10^-5^ |  | -0.42 (0.19-0.65) | 3.89×10^-4^ |
| rs200992 | 6 | 27814677 |  | G | 0.99 | Imputed | 0.12 | 0.10 | 2.25 (1.85-2.64) | 5.83×10^-5^ |  | -1.56 (1.11-2.02) | 1.81×10^-11^ |
| rs200485 | 6 | 27775697 |  | C | 0.99 | Imputed | 0.12 | 0.10 | 2.20 (1.80-2.59) | 8.70×10^-5^ |  | -1.56 (1.11-2.01) | 1.1×10^-11^ |
| rs200953 | 6 | 27837267 |  | C | 1.00 | Imputed | 0.12 | 0.10 | 2.15 (1.76-2.54) | 1.09×10^-4^ |  | -1.43 (0.98-1.88) | 4.55×10^-10^ |
| rs73062921 | 5 | 16859464 |  | A | 0.93 | Imputed | 0.15 | 0.09 | 1.68 (1.42-1.95) | 1.1×10^-4^ |  | -0.69 (0.35-1.04) | 8.8×10^-5^ |
| rs9789226 | 18 | 13804919 |  | G | 0.95 | Imputed | 0.24 | 0.14 | 1.57 (1.34-1.79) | 1.12×10^-4^ |  | -0.51 (0.23-0.8) | 4.51×10^-4^ |
| rs199696 | 1 | 77505413 |  | G | 0.94 | Imputed | 0.46 | 0.35 | 1.45 (1.26-1.64) | 1.35×10^-4^ |  | -0.41 (0.19-0.64) | 3.33×10^-4^ |
| rs71559050 | 6 | 27797416 |  | A | 0.99 | Imputed | 0.10 | 0.07 | 2.58 (2.09-3.06) | 1.37×10^-4^ |  | -1.98 (1.43-2.54) | 2.6×10^-12^ |
| rs34409918 | 6 | 27685348 |  | G | 0.97 | Imputed | 0.10 | 0.07 | 2.57 (2.08-3.06) | 1.37×10^-4^ |  | -1.98 (1.42-2.53) | 3.59×10^-12^ |
| rs13343310 | 19 | 52433467 |  | T | - | Genotyped | 0.36 | 0.30 | 1.46 (1.26-1.67) | 2.16×10^-4^ |  | -0.44 (0.2-0.67) | 3.00×10^-4^ |
| rs10152984 | 15 | 84016917 |  | T | 0.84 | Imputed | 0.46 | 0.39 | 1.42 (1.23-1.61) | 2.35×10^-4^ |  | -0.39 (0.17-0.61) | 4.25×10^-4^ |
| rs61782867 | 1 | 77511827 |  | A | 0.98 | Imputed | 0.18 | 0.11 | 1.59 (1.34-1.84) | 2.86×10^-4^ |  | -0.65 (0.32-0.99) | 1.27×10^-4^ |
| rs1233487 | 6 | 29468809 |  | G | 0.92 | Imputed | 0.31 | 0.23 | 1.58 (1.32-1.83) | 4.48×10^-4^ |  | -0.64 (0.34-0.95) | 3.59×10^-5^ |
| rs2914906 | 5 | 117983460 |  | G | 0.96 | Imputed | 0.36 | 0.31 | 1.40 (1.21-1.59) | 4.84×10^-4^ |  | -0.43 (0.2-0.66) | 2.45×10^-4^ |

Note: Chr, chromosome; EAF, effect allele frequency; OR, odds ratio; CI, confidence interval.

Rsq values: reflect imputation quality are obtained from output of Michigan Imputation Server.

# Table. S10. The variants associated with early-onset CRC risk in both two age cut-off groups in the GECCO cohort.

| SNP | Chr | Position | Effect allele | early-onset CRC (under 50 years old) | | early-onset CRC (under 40 years old) | | |
| --- | --- | --- | --- | --- | --- | --- | --- | --- |
|  |  |  |  | OR (95% CI) | *P* |  | OR (95% CI) | *P* |
|  |  |  |  |  |  |  |  |  |
| rs12794623 | 11 | 65029437 | A | 1.33 (1.21-1.45) | 4.41×10^-6^ |  | 1.63 (1.34-1.93) | 1.25×10^-3^ |
| rs401763 | 6 | 27782528 | C | 1.44 (1.28-1.60) | 4.71×10^-6^ |  | 2.12 (1.73-2.52) | 1.85×10^-4^ |
| rs401754 | 6 | 27782538 | A | 1.44 (1.28-1.60) | 4.71×10^-6^ |  | 2.12 (1.73-2.52) | 1.85×10^-4^ |
| rs200483 | 6 | 27774824 | A | 1.44 (1.28-1.59) | 5.91×10^-6^ |  | 2.14 (1.75-2.54) | 1.63×10^-4^ |
| rs200485 | 6 | 27775697 | C | 1.43 (1.28-1.59) | 5.92×10^-6^ |  | 2.20 (1.80-2.59) | 8.70×10^-5^ |
| rs200482 | 6 | 27773904 | A | 1.43 (1.27-1.58) | 6.45×10^-6^ |  | 2.18 (1.79-2.57) | 9.53×10^-5^ |
| rs200481 | 6 | 27773832 | A | 1.43 (1.27-1.59) | 6.65×10^-6^ |  | 2.20 (1.80-2.59) | 8.78×10^-5^ |
| rs200484 | 6 | 27775674 | G | 1.43 (1.28-1.59) | 6.95×10^-6^ |  | 2.14 (1.75-2.54) | 1.63×10^-4^ |
| rs9368531 | 6 | 27781872 | C | 1.43 (1.27-1.59) | 7.32×10^-6^ |  | 2.12 (1.72-2.51) | 1.94×10^-4^ |
| rs370155 | 6 | 27782031 | G | 1.42 (1.27-1.58) | 7.56×10^-6^ |  | 2.16 (1.76-2.55) | 1.14×10^-4^ |
| rs2747054 | 6 | 27783359 | G | 1.43 (1.27-1.59) | 7.81×10^-6^ |  | 2.12 (1.72-2.51) | 1.94×10^-4^ |
| rs486476 | 11 | 116312926 | G | 1.21 (1.13-1.30) | 1.17×10^-5^ |  | 1.29 (1.07-1.50) | 2.09×10^-2^ |
| rs200996 | 6 | 27811828 | A | 1.42 (1.26-1.57) | 1.40×10^-5^ |  | 2.14 (1.75-2.54) | 1.51×10^-4^ |
| rs200995 | 6 | 27813694 | C | 1.42 (1.26-1.58) | 1.43×10^-5^ |  | 2.17 (1.77-2.56) | 1.34×10^-4^ |
| rs200501 | 6 | 27788942 | T | 1.41 (1.25-1.57) | 1.55×10^-5^ |  | 2.03 (1.64-2.42) | 3.91×10^-4^ |
| rs175597 | 6 | 27810626 | C | 1.42 (1.26-1.57) | 1.66×10^-5^ |  | 2.15 (1.75-2.54) | 1.54×10^-4^ |
| rs200948 | 6 | 27835272 | C | 1.41 (1.25-1.57) | 1.73×10^-5^ |  | 2.12 (1.73-2.51) | 1.72×10^-4^ |
| rs200989 | 6 | 27816442 | G | 1.41 (1.26-1.57) | 1.74×10^-5^ |  | 2.18 (1.78-2.57) | 1.25×10^-4^ |
| rs34295134 | 6 | 27828151 | A | 1.41 (1.25-1.57) | 1.75×10^-5^ |  | 2.11 (1.71-2.50) | 1.88×10^-4^ |
| rs200983 | 6 | 27830326 | T | 1.41 (1.25-1.57) | 1.75×10^-5^ |  | 2.11 (1.71-2.50) | 1.88×10^-4^ |
| rs200954 | 6 | 27838764 | G | 1.41 (1.25-1.57) | 2.04×10^-5^ |  | 2.16 (1.77-2.56) | 1.28×10^-4^ |
| rs200950 | 6 | 27835772 | G | 1.41 (1.25-1.57) | 2.08×10^-5^ |  | 2.13 (1.74-2.52) | 1.60×10^-4^ |
| rs200952 | 6 | 27836976 | T | 1.41 (1.25-1.57) | 2.08×10^-5^ |  | 2.13 (1.74-2.52) | 1.60×10^-4^ |
| rs200981 | 6 | 27833174 | G | 1.40 (1.25-1.56) | 2.10×10^-5^ |  | 2.16 (1.77-2.54) | 1.02×10^-4^ |
| rs200489 | 6 | 27798257 | C | 1.41 (1.25-1.57) | 2.35×10^-5^ |  | 2.07 (1.67-2.47) | 3.44×10^-4^ |
| rs201002 | 6 | 27808192 | G | 1.40 (1.24-1.56) | 2.36×10^-5^ |  | 2.18 (1.79-2.57) | 9.02×10^-5^ |
| rs7582080 | 2 | 76839833 | A | 1.20 (1.11-1.28) | 2.42×10^-5^ |  | 1.28 (1.08-1.49) | 1.79×10^-2^ |
| rs200497 | 6 | 27792640 | T | 1.40 (1.25-1.56) | 2.53×10^-5^ |  | 2.05 (1.65-2.45) | 3.83×10^-4^ |
| rs138234416 | 6 | 27992898 | A | 1.50 (1.31-1.7) | 3.07×10^-5^ |  | 2.01 (1.52-2.49) | 4.86×10^-3^ |
| rs200992 | 6 | 27814677 | G | 1.40 (1.24-1.56) | 3.12×10^-5^ |  | 2.25 (1.85-2.64) | 5.83×10^-5^ |
| rs200990 | 6 | 27815823 | G | 1.39 (1.24-1.55) | 3.13×10^-5^ |  | 2.21 (1.82-2.60) | 7.24×10^-5^ |
| rs200490 | 6 | 27796935 | T | 1.39 (1.24-1.55) | 3.46×10^-5^ |  | 2.11 (1.71-2.50) | 2.05×10^-4^ |
| rs818176 | 2 | 10625156 | C | 1.21 (1.12-1.29) | 3.61×10^-5^ |  | 1.35 (1.12-1.57) | 9.00×10^-3^ |
| rs200977 | 6 | 27854301 | C | 1.40 (1.24-1.56) | 3.98×10^-5^ |  | 1.82 (1.40-2.24) | 5.22×10^-3^ |
| rs200953 | 6 | 27837267 | C | 1.38 (1.23-1.54) | 4.44×10^-5^ |  | 2.15 (1.76-2.54) | 1.09×10^-4^ |
| rs493161 | 6 | 27850714 | T | 1.40 (1.24-1.56) | 4.58×10^-5^ |  | 1.82 (1.40-2.24) | 5.10×10^-3^ |
| rs557042 | 6 | 27845129 | T | 1.40 (1.23-1.56) | 4.71×10^-5^ |  | 1.82 (1.40-2.24) | 5.10×10^-3^ |
| rs71537572 | 6 | 27970715 | C | 1.50 (1.30-1.69) | 4.82×10^-5^ |  | 1.87 (1.37-2.37) | 1.39×10^-2^ |
| rs71559067 | 6 | 27994416 | T | 1.49 (1.30-1.69) | 5.67×10^-5^ |  | 1.89 (1.39-2.39) | 1.28×10^-2^ |
| rs13193295 | 6 | 28003228 | G | 1.49 (1.30-1.69) | 5.67×10^-5^ |  | 1.89 (1.39-2.39) | 1.28×10^-2^ |
| rs17092631 | 11 | 116476765 | C | 1.32 (1.19-1.46) | 6.06×10^-5^ |  | 1.57 (1.24-1.90) | 8.23×10^-3^ |
| rs34203164 | 20 | 38956548 | T | 1.29 (1.17-1.42) | 6.48×10^-5^ |  | 1.38 (1.06-1.69) | 4.93×10^-2^ |
| rs36101351 | 6 | 27943369 | T | 1.49 (1.29-1.68) | 6.65×10^-5^ |  | 1.87 (1.37-2.37) | 1.39×10^-2^ |
| rs28360499 | 6 | 27945396 | A | 1.49 (1.29-1.68) | 6.65×10^-5^ |  | 1.87 (1.37-2.37) | 1.39×10^-2^ |
| rs13216117 | 6 | 27938484 | G | 1.49 (1.29-1.68) | 6.77×10^-5^ |  | 1.87 (1.37-2.37) | 1.39×10^-2^ |
| rs78893389 | 11 | 116475411 | T | 1.29 (1.16-1.41) | 9.72×10^-5^ |  | 1.44 (1.13-1.74) | 2.08×10^-2^ |
| rs34583951 | 20 | 38961391 | T | 1.28 (1.16-1.41) | 1.09×10^-4^ |  | 1.38 (1.06-1.70) | 4.86×10^-2^ |
| rs10848143 | 12 | 131025190 | G | 1.19 (1.10-1.27) | 1.21×10^-4^ |  | 1.35 (1.14-1.56) | 5.90×10^-3^ |
| rs138088344 | 2 | 53135287 | A | 1.31 (1.17-1.45) | 1.35×10^-4^ |  | 1.51 (1.19-1.83) | 1.24×10^-2^ |
| rs149498972 | 2 | 53135357 | A | 1.31 (1.17-1.45) | 1.37×10^-4^ |  | 1.51 (1.19-1.83) | 1.24×10^-2^ |
| rs483143 | 6 | 27846744 | C | 1.36 (1.20-1.52) | 1.46×10^-4^ |  | 1.80 (1.39-2.21) | 4.72×10^-3^ |
| rs200979 | 6 | 27852357 | A | 1.26 (1.14-1.38) | 1.46×10^-4^ |  | 1.39 (1.08-1.69) | 3.71×10^-2^ |
| rs200975 | 6 | 27855625 | T | 1.26 (1.14-1.38) | 1.48×10^-4^ |  | 1.39 (1.09-1.70) | 3.47×10^-2^ |
| rs200974 | 6 | 27855845 | G | 1.26 (1.14-1.38) | 1.48×10^-4^ |  | 1.39 (1.09-1.70) | 3.47×10^-2^ |
| rs200968 | 6 | 27859568 | C | 1.26 (1.14-1.38) | 1.49×10^-4^ |  | 1.40 (1.09-1.70) | 3.30×10^-2^ |
| rs67662114 | 6 | 27932301 | A | 1.46 (1.27-1.66) | 1.51×10^-4^ |  | 1.78 (1.28-2.29) | 2.52×10^-2^ |
| rs200965 | 6 | 27866384 | A | 1.26 (1.14-1.38) | 1.59×10^-4^ |  | 1.39 (1.08-1.69) | 3.66×10^-2^ |
| rs1450884 | 7 | 131844887 | C | 1.18 (1.09-1.26) | 1.61×10^-4^ |  | 1.24 (1.04-1.45) | 3.79×10^-2^ |
| rs67566584 | 20 | 38909950 | A | 1.28 (1.15-1.40) | 1.67×10^-4^ |  | 1.39 (1.07-1.71) | 4.28×10^-2^ |
| rs494887 | 6 | 27850873 | A | 1.26 (1.14-1.38) | 1.72×10^-4^ |  | 1.39 (1.08-1.69) | 3.71×10^-2^ |
| rs13195728 | 6 | 27771106 | C | 1.47 (1.27-1.66) | 1.76×10^-4^ |  | 2.62 (2.13-3.10) | 1.19×10^-4^ |
| rs200949 | 6 | 27835435 | G | 1.33 (1.18-1.48) | 1.82×10^-4^ |  | 1.70 (1.32-2.07) | 5.37×10^-3^ |
| rs200966 | 6 | 27862152 | C | 1.25 (1.14-1.37) | 1.90×10^-4^ |  | 1.41 (1.11-1.72) | 2.52×10^-2^ |
| rs169287 | 6 | 27854760 | A | 1.25 (1.14-1.37) | 1.92×10^-4^ |  | 1.41 (1.11-1.72) | 2.58×10^-2^ |
| rs200973 | 6 | 27858421 | G | 1.25 (1.14-1.37) | 1.93×10^-4^ |  | 1.42 (1.11-1.72) | 2.45×10^-2^ |
| rs200976 | 6 | 27854963 | G | 1.25 (1.13-1.37) | 1.95×10^-4^ |  | 1.41 (1.11-1.72) | 2.58×10^-2^ |
| rs11594481 | 10 | 133019289 | G | 1.28 (1.15-1.41) | 2.20×10^-4^ |  | 1.53 (1.20-1.85) | 1.08×10^-2^ |
| rs13197633 | 6 | 28174757 | A | 1.48 (1.27-1.69) | 2.23×10^-4^ |  | 2.13 (1.59-2.66) | 5.58×10^-3^ |
| rs72710971 | 8 | 127091691 | T | 1.48 (1.27-1.69) | 2.23×10^-4^ |  | 1.85 (1.36-2.35) | 1.46×10^-2^ |
| rs13195291 | 6 | 28169241 | A | 1.48 (1.27-1.69) | 2.42×10^-4^ |  | 2.13 (1.59-2.66) | 5.58×10^-3^ |
| rs17751184 | 6 | 27775028 | T | 1.46 (1.26-1.66) | 2.43×10^-4^ |  | 2.65 (2.16-3.14) | 1.02×10^-4^ |
| rs818154 | 2 | 10643637 | A | 1.15 (1.08-1.23) | 2.45×10^-4^ |  | 1.37 (1.18-1.55) | 9.88×10^-4^ |
| rs68190156 | 10 | 133018822 | G | 1.28 (1.15-1.41) | 2.45×10^-4^ |  | 1.53 (1.20-1.85) | 1.08×10^-2^ |
| rs200964 | 6 | 27866943 | C | 1.25 (1.13-1.37) | 2.48×10^-4^ |  | 1.41 (1.11-1.71) | 2.63×10^-2^ |
| rs34409918 | 6 | 27685348 | G | 1.46 (1.25-1.66) | 2.48×10^-4^ |  | 2.57 (2.08-3.06) | 1.37×10^-4^ |
| rs9649039 | 7 | 131845112 | T | 1.17 (1.09-1.26) | 2.52×10^-4^ |  | 1.26 (1.05-1.46) | 2.96×10^-2^ |
| rs11761595 | 7 | 131846030 | A | 1.17 (1.09-1.25) | 2.52×10^-4^ |  | 1.23 (1.03-1.44) | 4.60×10^-2^ |
| rs34432857 | 6 | 27685825 | A | 1.45 (1.25-1.65) | 2.52×10^-4^ |  | 2.57 (2.08-3.06) | 1.37×10^-4^ |
| rs34832585 | 6 | 28150878 | T | 1.48 (1.27-1.68) | 2.59×10^-4^ |  | 2.11 (1.58-2.65) | 6.01×10^-3^ |
| rs13217984 | 6 | 28139710 | T | 1.48 (1.27-1.68) | 2.61×10^-4^ |  | 2.11 (1.58-2.65) | 6.01×10^-3^ |
| rs67297533 | 6 | 28141253 | T | 1.48 (1.27-1.68) | 2.61×10^-4^ |  | 2.11 (1.58-2.65) | 6.01×10^-3^ |
| rs35781323 | 6 | 28144832 | C | 1.48 (1.27-1.68) | 2.61×10^-4^ |  | 2.11 (1.58-2.65) | 6.01×10^-3^ |
| rs72841331 | 10 | 133020295 | A | 1.28 (1.15-1.41) | 2.65×10^-4^ |  | 1.47 (1.15-1.80) | 1.89×10^-2^ |
| rs13194781 | 6 | 27815639 | G | 1.45 (1.25-1.65) | 2.69×10^-4^ |  | 2.62 (2.13-3.11) | 1.16×10^-4^ |
| rs36116761 | 6 | 27818482 | G | 1.45 (1.25-1.65) | 2.69×10^-4^ |  | 2.62 (2.13-3.11) | 1.16×10^-4^ |
| rs34194357 | 6 | 27818535 | G | 1.45 (1.25-1.65) | 2.69×10^-4^ |  | 2.62 (2.13-3.11) | 1.16×10^-4^ |
| rs13199772 | 6 | 27834085 | G | 1.45 (1.25-1.65) | 2.72×10^-4^ |  | 2.62 (2.13-3.11) | 1.12×10^-4^ |
| rs13199906 | 6 | 27834139 | G | 1.45 (1.25-1.65) | 2.72×10^-4^ |  | 2.62 (2.13-3.11) | 1.12×10^-4^ |
| rs13201308 | 6 | 28130089 | T | 1.47 (1.26-1.68) | 2.75×10^-4^ |  | 2.11 (1.58-2.65) | 6.01×10^-3^ |
| rs34765154 | 6 | 28130450 | A | 1.47 (1.26-1.68) | 2.75×10^-4^ |  | 2.11 (1.58-2.65) | 6.01×10^-3^ |
| rs34505829 | 6 | 28133239 | T | 1.47 (1.26-1.68) | 2.75×10^-4^ |  | 2.11 (1.58-2.65) | 6.01×10^-3^ |
| rs35098436 | 6 | 28134221 | C | 1.47 (1.26-1.68) | 2.75×10^-4^ |  | 2.11 (1.58-2.65) | 6.01×10^-3^ |
| rs71559050 | 6 | 27797416 | A | 1.45 (1.25-1.65) | 2.78×10^-4^ |  | 2.58 (2.09-3.06) | 1.37×10^-4^ |
| rs17763089 | 6 | 27835218 | A | 1.45 (1.25-1.65) | 2.79×10^-4^ |  | 2.62 (2.13-3.11) | 1.12×10^-4^ |
| rs13212318 | 6 | 27688841 | C | 1.45 (1.25-1.65) | 2.80×10^-4^ |  | 2.57 (2.08-3.06) | 1.37×10^-4^ |
| rs13197175 | 6 | 28129231 | T | 1.47 (1.26-1.68) | 2.82×10^-4^ |  | 2.11 (1.58-2.65) | 6.01×10^-3^ |
| rs13197176 | 6 | 28129232 | T | 1.47 (1.26-1.68) | 2.82×10^-4^ |  | 2.11 (1.58-2.65) | 6.01×10^-3^ |
| rs35819751 | 6 | 27810569 | G | 1.45 (1.25-1.65) | 2.82×10^-4^ |  | 2.58 (2.09-3.06) | 1.37×10^-4^ |
| rs818174 | 2 | 10626376 | T | 1.15 (1.08-1.23) | 2.83×10^-4^ |  | 1.38 (1.20-1.57) | 6.62×10^-4^ |
| rs17695758 | 6 | 27837183 | C | 1.45 (1.25-1.65) | 2.86×10^-4^ |  | 2.62 (2.13-3.11) | 1.12×10^-4^ |
| rs34706883 | 6 | 27805255 | C | 1.45 (1.25-1.65) | 2.88×10^-4^ |  | 2.57 (2.08-3.06) | 1.44×10^-4^ |
| rs13202291 | 6 | 27698857 | G | 1.45 (1.25-1.65) | 2.93×10^-4^ |  | 2.57 (2.08-3.06) | 1.37×10^-4^ |
| rs10218752 | 1 | 163171868 | C | 1.18 (1.09-1.27) | 2.93×10^-4^ |  | 1.57 (1.35-1.78) | 3.72×10^-5^ |
| rs35501037 | 6 | 27739566 | A | 1.44 (1.24-1.64) | 3.02×10^-4^ |  | 2.52 (2.03-3.00) | 1.80×10^-4^ |
| rs34064842 | 6 | 27688625 | T | 1.44 (1.24-1.64) | 3.03×10^-4^ |  | 2.52 (2.03-3.00) | 1.80×10^-4^ |
| rs13193480 | 6 | 27702561 | G | 1.45 (1.25-1.65) | 3.06×10^-4^ |  | 2.57 (2.08-3.06) | 1.37×10^-4^ |
| rs17750424 | 6 | 27701122 | C | 1.45 (1.25-1.65) | 3.06×10^-4^ |  | 2.57 (2.08-3.06) | 1.37×10^-4^ |
| rs13193542 | 6 | 27702425 | T | 1.45 (1.25-1.65) | 3.06×10^-4^ |  | 2.57 (2.08-3.06) | 1.37×10^-4^ |
| rs13205911 | 6 | 28124114 | T | 1.47 (1.26-1.68) | 3.14×10^-4^ |  | 2.11 (1.58-2.65) | 6.01×10^-3^ |
| rs34662244 | 6 | 28073881 | A | 1.47 (1.26-1.67) | 3.18×10^-4^ |  | 2.10 (1.56-2.63) | 6.48×10^-3^ |
| rs35749575 | 6 | 28114818 | T | 1.47 (1.26-1.67) | 3.18×10^-4^ |  | 2.11 (1.58-2.65) | 6.01×10^-3^ |
| rs72846780 | 6 | 28119055 | C | 1.47 (1.26-1.67) | 3.18×10^-4^ |  | 2.11 (1.58-2.65) | 6.01×10^-3^ |
| rs34718920 | 6 | 27783941 | T | 1.45 (1.24-1.65) | 3.21×10^-4^ |  | 2.58 (2.09-3.06) | 1.38×10^-4^ |
| rs13197574 | 6 | 28060239 | C | 1.47 (1.26-1.67) | 3.29×10^-4^ |  | 2.11 (1.58-2.65) | 6.01×10^-3^ |
| rs35902873 | 6 | 28058949 | C | 1.47 (1.26-1.67) | 3.29×10^-4^ |  | 2.11 (1.58-2.65) | 6.01×10^-3^ |
| rs34166054 | 6 | 28065801 | C | 1.47 (1.26-1.67) | 3.29×10^-4^ |  | 2.11 (1.58-2.65) | 6.01×10^-3^ |
| rs67101035 | 6 | 27798887 | G | 1.44 (1.24-1.64) | 3.34×10^-4^ |  | 2.57 (2.08-3.06) | 1.44×10^-4^ |
| rs13212651 | 6 | 27806985 | G | 1.44 (1.24-1.64) | 3.34×10^-4^ |  | 2.57 (2.08-3.06) | 1.44×10^-4^ |
| rs818155 | 2 | 10643327 | G | 1.15 (1.07-1.22) | 3.36×10^-4^ |  | 1.36 (1.17-1.55) | 1.16×10^-3^ |
| rs34371502 | 6 | 28081758 | C | 1.46 (1.25-1.67) | 3.37×10^-4^ |  | 2.10 (1.56-2.63) | 6.48×10^-3^ |
| rs13203816 | 6 | 28079598 | C | 1.46 (1.25-1.67) | 3.37×10^-4^ |  | 2.10 (1.56-2.63) | 6.48×10^-3^ |
| rs34588114 | 6 | 28080628 | A | 1.46 (1.25-1.67) | 3.37×10^-4^ |  | 2.10 (1.56-2.63) | 6.48×10^-3^ |
| rs68188794 | 6 | 28080777 | C | 1.46 (1.25-1.67) | 3.37×10^-4^ |  | 2.10 (1.56-2.63) | 6.48×10^-3^ |
| rs34788973 | 6 | 27879200 | A | 1.45 (1.25-1.65) | 3.42×10^-4^ |  | 2.21 (1.70-2.72) | 2.33×10^-3^ |
| rs61742093 | 6 | 27879982 | G | 1.45 (1.25-1.65) | 3.42×10^-4^ |  | 2.21 (1.70-2.72) | 2.33×10^-3^ |
| rs71559072 | 6 | 28089481 | T | 1.46 (1.25-1.67) | 3.42×10^-4^ |  | 2.11 (1.58-2.65) | 6.01×10^-3^ |
| rs66886492 | 6 | 28089731 | T | 1.46 (1.25-1.67) | 3.42×10^-4^ |  | 2.11 (1.58-2.65) | 6.01×10^-3^ |
| rs35345226 | 6 | 28091580 | G | 1.46 (1.25-1.67) | 3.42×10^-4^ |  | 2.11 (1.58-2.65) | 6.01×10^-3^ |
| rs72847313 | 6 | 27730082 | T | 1.44 (1.24-1.64) | 3.47×10^-4^ |  | 2.52 (2.03-3.00) | 1.80×10^-4^ |
| rs17750747 | 6 | 27730334 | C | 1.44 (1.24-1.64) | 3.47×10^-4^ |  | 2.52 (2.03-3.00) | 1.80×10^-4^ |
| rs71559070 | 6 | 28038929 | A | 1.46 (1.25-1.67) | 3.52×10^-4^ |  | 2.11 (1.58-2.65) | 6.01×10^-3^ |
| rs9552210 | 13 | 21067941 | G | 1.16 (1.08-1.24) | 3.54×10^-4^ |  | 1.35 (1.15-1.54) | 2.93×10^-3^ |
| rs13199649 | 6 | 27868792 | T | 1.46 (1.25-1.67) | 3.67×10^-4^ |  | 2.10 (1.56-2.63) | 6.40×10^-3^ |
| rs431845 | 13 | 1.13E+08 | C | 1.31 (1.16-1.46) | 3.68×10^-4^ |  | 1.67 (1.32-2.01) | 3.68×10^-3^ |
| rs4675566 | 2 | 2.07E+08 | C | 1.17 (1.08-1.26) | 3.69×10^-4^ |  | 1.30 (1.08-1.51) | 1.70×10^-2^ |
| rs35037868 | 6 | 27759115 | C | 1.45 (1.24-1.65) | 3.86×10^-4^ |  | 2.19 (1.68-2.71) | 2.89×10^-3^ |
| rs13218875 | 6 | 27884012 | T | 1.45 (1.25-1.66) | 4.21×10^-4^ |  | 2.10 (1.56-2.63) | 6.40×10^-3^ |
| rs9509249 | 13 | 21068045 | C | 1.16 (1.07-1.24) | 4.23×10^-4^ |  | 1.34 (1.15-1.54) | 3.16×10^-3^ |
| rs10958911 | 9 | 10135016 | A | 1.21 (1.11-1.32) | 4.26×10^-4^ |  | 1.44 (1.18-1.69) | 5.21×10^-3^ |
| rs13200214 | 6 | 28017250 | T | 1.45 (1.24-1.66) | 4.36×10^-4^ |  | 2.12 (1.59-2.65) | 5.80×10^-3^ |
| rs71559054 | 6 | 27896799 | C | 1.45 (1.24-1.66) | 4.42×10^-4^ |  | 2.10 (1.56-2.63) | 6.40×10^-3^ |
| rs187956 | 1 | 77508652 | G | 1.15 (1.07-1.23) | 4.58×10^-4^ |  | 1.45 (1.26-1.64) | 1.41×10^-4^ |

Note: Chr, chromosome; OR, odds ratio; CI, confidence interval.

# Table. S11. 88 mapped genes of 211 EOCRC specific risk loci.

| Gene ID | Gene Symbol | Description |
| --- | --- | --- |
| 823 | *CAPN1* | calpain 1, (mu/I) large subunit |
| 1936 | *EEF1D* | eukaryotic translation elongation factor 1 delta (guanine nucleotide exchange protein) |
| 3009 | *HIST1H1B* | histone cluster 1, H1b |
| 4289 | *MKLN1* | muskelin 1, intracellular mediator containing kelch motifs |
| 4953 | *ODC1* | ornithine decarboxylase 1 |
| 5789 | *PTPRD* | protein tyrosine phosphatase, receptor type, D |
| 5820 | *PVT1* | Pvt1 oncogene (non-protein coding) |
| 7212 | *TRNAM2* | transfer RNA methionine 2 (anticodon CAU) |
| 7264 | *TSTA3* | tissue specific transplantation antigen P35B |
| 7718 | *ZNF165* | zinc finger protein 165 |
| 7745 | *ZKSCAN8* | zinc finger with KRAB and SCAN domains 8 |
| 7746 | *ZSCAN9* | zinc finger and SCAN domain containing 9 |
| 8329 | *HIST1H2AI* | histone cluster 1, H2ai |
| 8330 | *HIST1H2AK* | histone cluster 1, H2ak |
| 8331 | *HIST1H2AJ* | histone cluster 1, H2aj |
| 8332 | *HIST1H2AL* | histone cluster 1, H2al |
| 8336 | *HIST1H2AM* | histone cluster 1, H2am |
| 8340 | *HIST1H2BL* | histone cluster 1, H2bl |
| 8341 | *HIST1H2BN* | histone cluster 1, H2bn |
| 8342 | *HIST1H2BM* | histone cluster 1, H2bm |
| 8348 | *HIST1H2BO* | histone cluster 1, H2bo |
| 8354 | *HIST1H3I* | histone cluster 1, H3i |
| 8356 | *HIST1H3J* | histone cluster 1, H3j |
| 8357 | *HIST1H3H* | histone cluster 1, H3h |
| 8362 | *HIST1H4K* | histone cluster 1, H4k |
| 8363 | *HIST1H4J* | histone cluster 1, H4j |
| 8368 | *HIST1H4L* | histone cluster 1, H4l |
| 8490 | *RGS5* | regulator of G-protein signaling 5 |
| 10026 | *PIGK* | phosphatidylinositol glycan anchor biosynthesis, class K |
| 23144 | *ZC3H3* | zinc finger CCCH-type containing 3 |
| 23250 | *ATP11A* | ATPase, class VI, type 11A |
| 23504 | *RIMBP2* | RIMS binding protein 2 |
| 23649 | *POLA2* | polymerase (DNA directed), alpha 2, accessory subunit |
| 26212 | *OR2B6* | olfactory receptor, family 2, subfamily B, member 6 |
| 30844 | *EHD4* | EH-domain containing 4 |
| 51084 | *CRYL1* | crystallin, lambda 1 |
| 55625 | *ZDHHC7* | zinc finger, DHHC-type containing 7 |
| 65263 | *PYCRL* | pyrroline-5-carboxylate reductase-like |
| 79792 | *GSDMD* | gasdermin D |
| 80345 | *ZSCAN16* | zinc finger and SCAN domain containing 16 |
| 81697 | *OR2B2* | olfactory receptor, family 2, subfamily B, member 2 |
| 81849 | *ST6GALNAC5* | ST6 (alpha-N-acetyl-neuraminyl-2,3-beta-galactosyl-1,3)-N-acetylgalactosaminide alpha-2,6-sialyltransferase 5 |
| 83666 | *PARP9* | poly (ADP-ribose) polymerase family, member 9 |
| 83716 | *CRISPLD2* | cysteine-rich secretory protein LCCL domain containing 2 |
| 84948 | *TIGD5* | tigger transposable element derived 5 |
| 91584 | *PLXNA4* | plexin A4 |
| 93100 | *NAPRT1* | nicotinate phosphoribosyltransferase domain containing 1 |
| 123745 | *PLA2G4E* | phospholipase A2, group IVE |
| 137964 | *AGPAT6* | 1-acylglycerol-3-phosphate O-acyltransferase 6 |
| 151636 | *DTX3L* | deltex 3-like (Drosophila) |
| 157848 | *NKX6-3* | NK6 homeobox 3 |
| 165631 | *PARP15* | poly (ADP-ribose) polymerase family, member 15 |
| 256536 | *TCERG1L* | transcription elongation regulator 1-like |
| 339978 | *LOC339978* | uncharacterized LOC339978 |
| 387032 | *ZKSCAN4* | zinc finger with KRAB and SCAN domains 4 |
| 401164 | *LINC01060* | long intergenic non-protein coding RNA 1060 |
| 440044 | *SLC22A20* | solute carrier family 22, member 20 |
| 440279 | *UNC13C* | unc-13 homolog C (C. elegans) |
| 442179 | *OR1F12* | olfactory receptor, family 1, subfamily F, member 12 |
| 642475 | *MROH6* | maestro heat-like repeat family member 6 |
| 791230 | *TRNAV7* | transfer RNA valine 7 (anticodon AAC) |
| 100129195 | *ZSCAN16-AS1* | ZSCAN16 antisense RNA 1 |
| 100131289 | *LOC100131289* | uncharacterized LOC100131289 |
| 100189071 | *TRNAI4* | transfer RNA isoleucine 4 (anticodon AAU) |
| 100189074 | *TRNAQ10* | transfer RNA glutamine 10 (anticodon UUG) |
| 100189095 | *TRNAI5* | transfer RNA isoleucine 5 (anticodon AAU) |
| 100189101 | *TRNAV15* | transfer RNA valine 15 (anticodon CAC) |
| 100189159 | *TRNAS13* | transfer RNA serine 13 (anticodon GCU) |
| 100189161 | *TRNAV17* | transfer RNA valine 17 (anticodon AAC) |
| 100189164 | *TRNAL16* | transfer RNA leucine 16 (anticodon UAA) |
| 100189205 | *TRNAS15* | transfer RNA serine 15 (anticodon CGA) |
| 100189214 | *TRNAQ17* | transfer RNA glutamine 17 (anticodon CUG) |
| 100189314 | *TRNAG29* | transfer RNA glycine 29 (anticodon GCC) |
| 100189315 | *TRNAM17* | transfer RNA methionine 17 (anticodon CAU) |
| 100189340 | *TRNAT15* | transfer RNA threonine 15 (anticodon AGU) |
| 100189374 | *TRNAT18* | transfer RNA threonine 18 (anticodon AGU) |
| 100189428 | *TRNAR29* | transfer RNA arginine 29 (anticodon ACG) |
| 100302170 | *MIR1206* | microRNA 1206 |
| 100302175 | *MIR1207* | microRNA 1207 |
| 100302743 | *SNORA80B* | small nucleolar RNA, H/ACA box 80B |
| 100507173 | *LINC01012* | long intergenic non-protein coding RNA 1012 |
| 100874205 | *ATP11A-AS1* | ATP11A antisense RNA 1 |
| 100996513 | *LOC100996513* | uncharacterized LOC100996513 |
| 101928388 | *LOC101928388* | uncharacterized LOC101928388 |
| 101928404 | *LOC101928404* | uncharacterized LOC101928404 |
| 101928474 | *LOC101928474* | uncharacterized LOC101928474 |
| 101929011 | *LOC101929011* | uncharacterized LOC101929011 |
| 101929715 | *LOC101929715* | uncharacterized LOC101929715 |

# Table. S12. Risk estimates for early-onset CRC associated with different PRS scores in the GECCO cohort.

| PRS | *N* (cases) | *N* (controls) | OR (95% CI) | *P* |
| --- | --- | --- | --- | --- |
| PRS (GWAS) |  |  |  |  |
| Quartile 1 (ref) | 230 | 5131 | Ref | Ref |
| Quartile 2 | 337 | 5023 | 1.49 (1.26-1.77) | 4.36×10^-6^ |
| Quartile 3 | 398 | 4962 | 1.78 (1.51-2.11) | 8.46×10^-12^ |
| Quartile 4 | 525 | 4835 | 2.42 (2.06-2.84) | 2.08×10^-27^ |
| PRS (Early-onset) |  |  |  |  |
| Quartile 1 (ref) | 192 | 5169 | Ref |  |
| Quartile 2 | 270 | 5090 | 1.42 (1.18-1.72) | 2.20×10^-4^ |
| Quartile 3 | 367 | 4993 | 1.97 (1.65-2.36) | 7.46×10^-14^ |
| Quartile 4 | 661 | 4699 | 3.79 (3.21-4.47) | 4.80×10^-56^ |
| PRS (Combined) |  |  |  |  |
| Quartile 1 (ref) | 157 | 5204 | Ref | Ref |
| Quartile 2 | 254 | 5106 | 1.64 (1.34-2.01) | 1.32×10^-6^ |
| Quartile 3 | 411 | 4949 | 2.75 (2.28-3.32) | 4.62×10^-26^ |
| Quartile 4 | 668 | 4692 | 4.71 (3.94-5.64) | 2.93×10^-65^ |

Note: Ref, reference; OR, odds ratio; CI, confidence interval; PRS, polygenic risk score.

The logistic regression models include sex and polygenic risk score.

# Table. S13. Risk estimates for early-onset CRC associated with different PRS scores in the UKB cohort.

| PRS | *N* (cases) | *N* (controls) | OR (95% CI) | *P* |
| --- | --- | --- | --- | --- |
| PRS (GWAS) |  |  |  |  |
| Quartile 1 (ref) | 140 | 6148 | Ref | Ref |
| Quartile 2 | 174 | 6113 | 1.28 (1.01-1.64) | 0.044 |
| Quartile 3 | 184 | 6103 | 1.61 (1.27-2.03) | 7.06×10^-5^ |
| Quartile 4 | 225 | 6063 | 2.39 (1.92-2.98) | 6.69×10^-15^ |
| PRS (Early-onset) |  |  |  |  |
| Quartile 1 (ref) | 117 | 6171 | Ref | Ref |
| Quartile 2 | 149 | 6138 | 1.25 (1.00-1.57) | 0.052 |
| Quartile 3 | 185 | 6102 | 1.32 (1.06-1.65) | 0.014 |
| Quartile 4 | 272 | 6016 | 1.63 (1.31-2.02) | 6.73×10^-6^ |
| PRS (Combined) |  |  |  |  |
| Quartile 1 (ref) | 97 | 6191 | Ref | Ref |
| Quartile 2 | 157 | 6130 | 1.64 (1.27-2.12) | 1.45×10^-4^ |
| Quartile 3 | 186 | 6101 | 1.95 (1.52-2.50) | 1.18×10^-7^ |
| Quartile 4 | 283 | 6005 | 3.02 (2.39-3.81) | 1.61×10^-20^ |

Note: Ref, reference; OR, odds ratio; CI, confidence interval; PRS, polygenic risk score.

The logistic regression models include sex and polygenic risk score.

# Table. S14. Risk estimates for early-onset CRC associated with different PRS scores with adjustment of sociodemographic factors in the UKB cohort.

| PRS | *N* (cases) | *N* (controls) | OR (95% CI) | *P* |
| --- | --- | --- | --- | --- |
| PRS (GWAS) |  |  |  |  |
| Quartile 1 (ref) | 140 | 6148 | Ref | Ref |
| Quartile 2 | 174 | 6113 | 1.28 (1.01-1.64) | 0.045 |
| Quartile 3 | 184 | 6103 | 1.62 (1.28-2.04) | 6.15×10^-5^ |
| Quartile 4 | 225 | 6063 | 2.39 (1.92-2.98) | 7.51×10^-15^ |
| PRS (Early-onset) |  |  |  |  |
| Quartile 1 (ref) | 117 | 6171 | Ref | Ref |
| Quartile 2 | 149 | 6138 | 1.25 (1.00-1.57) | 0.050 |
| Quartile 3 | 185 | 6102 | 1.32 (1.06-1.65) | 0.014 |
| Quartile 4 | 272 | 6016 | 1.64 (1.32-2.03) | 5.93×10^-6^ |
| PRS (Combined) |  |  |  |  |
| Quartile 1 (ref) | 97 | 6191 | Ref | Ref |
| Quartile 2 | 157 | 6130 | 1.64 (1.27-2.12) | 1.39×10^-4^ |
| Quartile 3 | 186 | 6101 | 1.96 (1.53-2.52) | 1.01×10^-7^ |
| Quartile 4 | 283 | 6005 | 3.02 (2.39-3.82) | 1.69×10^-20^ |

Note: Ref, reference; OR, odds ratio; CI, confidence interval; PRS, polygenic risk score.

The logistic regression models were adjusted for sex, ethnicity, drinking frequency, smoke status, and family history, and polygenic risk score.

# Table. S15. The potential functional annotations of the 49 candidate variants.

| Variants | Chr | Pos. | Ref | Alt | Total score | 3DSNP score | CADD score | RegulomeDB score | Chromatin Marks | TF Motifs | Nearby genes |
| --- | --- | --- | --- | --- | --- | --- | --- | --- | --- | --- | --- |
| **rs12794623** | **11** | **65029437** | **C** | **A** | **2.21** | **206.39** | **9.35** | **0.61** | **H3K4me3_Pro; H3K9ac_Pro; H3K27ac_Enh; H3K4me1_Enh** | **GR; Hic1** | *POLA2* |
| rs200948 | 6 | 27835272 | T | C | 1.91 | 217.08 | 6.62 | 0.67 | H3K4me3_Pro; H3K9ac_Pro; H3K27ac_Enh; H3K4me1_Enh | E2F; SREBP; SREBP; YY1; Znf143 | *HIST1H4J* |
| rs401763 | 6 | 27782528 | T | C | 1.54 | 192.94 | 4.73 | 0.62 | H3K4me3_Pro; H3K9ac_Pro; H3K27ac_Enh; H3K4me1_Enh | FXR; HNF4; PPAR; | *TRNAM2* |
| rs35501037 | 6 | 27739566 | T | A | 1.27 | 25.85 | 10.72 | 0.61 | H3K4me1_Enh; H3K4me3_Pro; H3K9ac_Pro; H3K27ac_Enh | Cdx; Foxd3 | *TRNAT18* |
| rs818174 | 2 | 10626376 | A | T | 1.00 | 2.39 | 10.4 | 0.11 | H3K9ac_Pro | AP-1; Irf; Lhx3; PRDM1 | *ODC1* |
| rs10505506 | 8 | 129045291 | C | G | 0.73 | 35.09 | 7.14 | 0.61 | H3K27ac_Enh; H3K4me1_Enh | VDR | *PVT1* |
| rs13197175 | 6 | 28129231 | C | T | 0.68 | 131.46 | 1.52 | 0.57 | H3K4me3_Pro; H3K9ac_Pro; H3K4me1_Enh; H3K27ac_Enh |  | *ZNF602P* |
| rs17092631 | 11 | 116476765 | T | C | 0.57 | 1.88 | 5.08 | 0.61 |  | BDP1; Ets; NERF1a; NF-I; RXRA; SRF | *LOC101929011* |
| rs10218752 | 1 | 163171868 | T | C | 0.56 | 22.98 | 5.94 | - | H3K4me3_Pro | DMRT3; Smad | *RGS5* |
| rs16974504 | 15 | 54612426 | A | G | 0.53 | 0.99 | 7.22 | - |  | HNF1; YY1 | *UNC13C* |
| rs10958911 | 9 | 10135016 | T | A | 0.51 | 1.17 | 3.01 | 0.64 |  | Dbx1; HMG-IY; HP1-site-factor; TATA | *PTPRD* |
| rs200965 | 6 | 27866384 | G | A | 0.41 | 44.82 | 4.21 | 0.55 | H3K9ac_Pro | EWSR1-FLI1; TCF12 | *HIST1H2BN* |
| rs17043770 | 2 | 53132960 | A | G | 0.33 | 1.34 | 4.37 | 0.33 |  | Barhl1; Barx1; CDP; CEBPB; Cphx; and other 11 motifs |  |
| rs28510083 | 4 | 55456806 | C | T | 0.32 | 2.58 | 5.24 | 0.61 |  | HDAC2; SP1 | *LOC339978* |
| rs34203164 | 20 | 38956548 | C | T | 0.31 | 1.79 | 5.87 | 0.18 |  | Maf; Nrf-2 |  |
| rs7668968 | 4 | 66726174 | A | G | 0.29 | 3.3 | 5.37 | 0.13 |  | Lmo2-complex; Rad21; TCF12 |  |
| rs9672119 | 14 | 62961239 | C | T | 0.28 | 6.68 | 1.5 | 0.18 |  | Mrg; Sin3Ak-20; Zfp410 |  |
| rs1620412 | 15 | 42308065 | A | G | 0.17 | 2.33 | 0.39 | 0.13 |  | AP-4; ATF3 | *EHD4* |
| rs1996878 | 4 | 189600503 | C | A | 0.14 | 1.65 | 3.69 | - |  | Maf; Pou2f2; Sox | *LINC01060* |
| rs187956 | 1 | 77508652 | A | G | 0.08 | 0.85 | 3.32 | 0.61 |  | Maf; Myb | *ST6GALNAC5* |
| rs17600200 | 8 | 41455004 | A | G | 0.06 | 4.19 | 1.11 | 0.13 |  | Otx2 | *AGPAT6* |
| rs1450884 | 7 | 131844887 | G | C | -0.06 | 1.53 | 2.2 | 0.94 |  | Foxp1; RREB-1 | *PLXNA4* |
| rs10848143 | 12 | 131025190 | G | C | -0.07 | 1.56 | 3.56 | 0.6 |  | Cart1; MAZ; p300 | *RIMBP2* |
| rs4675566 | 2 | 206787764 | T | C | -0.11 | 2.02 | 1.77 | 0.13 | H3K4me1_Enh | EBF; NF-kappaB; Smad |  |
| rs34409918 | 6 | 27685348 | A | G | -0.19 | 1.09 | 0.97 | 0.13 |  | Pax-4 | *TRNAI5* |
| rs73149853 | 7 | 130883780 | G | A | -0.26 | 1.94 | 4.55 | 0.41 |  | Arid3a; Barhl1; En-1; Gbx1; Gbx2; and other 17 motifs | *MKLN1* |
| rs138234416 | 6 | 27992898 | G | A | -0.28 | 3.99 | 2.2 | 0.18 |  | AP-1; Foxa; Foxj2; Irf; RREB-1 | *OR2W4P* |
| rs13199649 | 6 | 27868792 | C | T | -0.29 | 17.21 | 4.05 | 0.59 |  | Lhx8; Myc | *HIST1H2BN* |
| rs17078858 | 13 | 85537208 | C | T | -0.30 | 1.24 | 0.4 | 0.13 |  | PU.1; SPIB; p53 |  |
| rs11594481 | 10 | 133019289 | A | G | -0.32 | 3.27 | 4.05 | 0.03 |  | AP-2; CACD; CTCF; Nkx2; RXRA; and other 5 motifs | *TCERG1L* |
| rs9602610 | 13 | 85533819 | A | G | -0.42 | 1.33 | 3.88 | 0.18 |  | Pou2f2 |  |
| rs431845 | 13 | 113421026 | T | C | -0.49 | 5.09 | 1.29 | 0.7 |  | PRDM1; Sin3Ak-20; Spz1; YY1 | *ATP11A* |
| rs13197633 | 6 | 28174757 | G | A | -0.52 | 8.22 | 2.76 | 0.64 | H3K4me3_Pro; H3K9ac_Pro; H3K4me1_Enh; H3K27ac_Enh | FAC1; Foxo; HDAC2; Zfp105; Zfp691 | *ZKSCAN8* |
| rs11848737 | 14 | 62956957 | T | C | -0.53 | 0.51 | 1.58 | - |  | GATA; Pou1f1; Pou2f2 |  |
| rs200977 | 6 | 27854301 | T | C | -0.54 | 18.19 | 1.63 | 0.55 |  | CDP; Crx; Irf; Otx2 | *HIST1H2BN* |
| rs35702572 | 14 | 63087698 | T | C | -0.56 | 0.59 | 3.74 | 0.18 |  |  | *ATP5A1P4* |
| rs10139477 | 14 | 36444620 | A | G | -0.59 | 0.67 | 2.77 | 0.99 |  | AIRE; Evi-1; PEBP; Sox |  |
| rs486476 | 11 | 116312926 | C | G | -0.61 | 1.11 | 2.42 | 0.15 |  | DEC; Foxp1 |  |
| rs967699 | 8 | 144646890 | G | T | -0.67 | 4.94 | 1.26 | - | H3K4me3_Pro; H3K9ac_Pro; H3K27ac_Enh; H3K4me1_Enh | NF-AT; NF-AT1; STAT; TCF4 | *ZC3H3* |
| rs7071 | 3 | 122293773 | A | T | -0.70 | 2.8 | 1.63 | 0.45 | H3K9ac_Pro | Arid3a; Foxo; Gfi1; Irx; Mef2; TATA; Zfp105 | *PARP9* |
| rs12137323 | 1 | 222125528 | G | A | -0.71 | 1.7 | 2.5 | 0.13 | H3K4me3_Pro |  |  |
| rs7582080 | 2 | 76839833 | G | A | -0.71 | 2.42 | 1.29 | 1 |  | ATF2; CEBPA; E4BP4; GR; Gmeb1 |  |
| rs9552210 | 13 | 21067941 | G | A | -0.72 | 3.07 | 0.51 | 0.61 |  | GR; HDAC2; Maf; Mef2; PLZF; Sin3Ak-20 | *CRYL1* |
| rs1874629 | 16 | 84960383 | G | A | -0.73 | 1.21 | 1.45 | 0.81 | H3K9ac_Pro | HDAC2; Hsf; NF-AT; STAT | *CRISPLD2* |
| rs2358662 | 14 | 54807541 | T | C | -0.77 | 1.45 | 1.17 | 0.16 |  | BDP1; Egr-1; PU.1; STAT; UF1H3BETA |  |
| rs200996 | 6 | 27811828 | G | A | -0.77 | 9.46 | 0.28 | 0.82 |  | Arid3a; LXR; Mef2 | *TRNAQ10* |
| rs818154 | 2 | 10643637 | G | A | -0.79 | 1.51 | 0.8 | 0.13 |  | Hsf |  |
| rs72710971 | 8 | 127091691 | C | T | -0.81 | 1.8 | 0.1 | 0.61 |  | RXRA | *KNOP1P5* |
| rs818176 | 2 | 10625156 | A | C | -0.84 | 2.15 | 0.59 | 0.03 |  | GR; HMG-IY; Ik-3; Lhx3; Nkx6-1; Otx2; Pou3f4; STAT | *ODC1* |

Note: Chr, chromosome; Pos., position; Ref, reference allele; Alt, alternate allele; TF: transcription factor; Enh: Enhancer; Pro: promoter.

Total score is the mean value of respective Z-scores of RegulomeDB score, CADD score and 3DSNP score.

The chromatin marks annotations and protein binding annotations from the Roadmap Epigenomics and ENCODE projects in sigmoid colon, colonic mucosa, and rectal mucosa tissues.

HaploReg database, https://pubs.broadinstitute.org/mammals/haploreg/haploreg.php; Roadmap Epigenomics Consortium, https://www.ncbi.nlm.nih.gov/geo/roadmap/epigenomics/; ENCODE database, http://compbio.mit.edu/encode-motifs/; RegulomeDB Score: https://www.regulomedb.org/regulome-search; CADD Score: https://cadd.gs.washington.edu/score; 3DSNP Score: https://omic.tech/3dsnpv2/.
