# Supplementary material for: Identification of specific susceptibility loci for the early-onset colorectal cancer
Source: Genome Med. 2023 Mar 3;15:13. doi: 10.1186/s13073-023-01163-w (PMC9983269; doi:10.1186/s13073-023-01163-w)
Supplement: Supplementary file 3 — Additional file 3. The codes used for statistical analysis and generation of tables and figures. [file 13073_2023_1163_MOESM3_ESM.docx]

**Identification of specific susceptibility loci for the early-onset colorectal cancer**

**Additional file 3**

# Text S1: The codes used for statistical analysis and generation of tables and figures

## principal component analysis

perl smartpca.perl -i example.bed -a example.bim -b example.ind -k 10 -o example.pca -p example.plot -e example.eval -l example.log -m 0

./smartpca -p all_dataset.pca.par

-i example.geno : genotype file in any format

-a example.snp : snp file in any format

-b example.ind : indiv file in any format

-k k : (Default is 10) number of principal components to output

-o example.pca : output file of principal components. Individuals removed

as outliers will have all values set to 0.0 in this file.

-p example.plot : prefix of output plot files of top 2 principal components.

(labeling individuals according to labels in indiv file)

-e example.eval : output file of all eigenvalues

-l example.log : output logfile

-m maxiter : (Default is 5) maximum number of outlier removal iterations.

To turn off outlier removal, set -m 0.

-t topk : (Default is 10) number of principal components along which

to remove outliers during each outlier removal iteration.

-s sigma : (Default is 6.0) number of standard deviations which an

individual must exceed, along one of topk top principal

components, in order to be removed as an outlier.

## association analysis

plink --bfile file_mind_file_qcsample --hwe 0.00001 --geno 0.02 --maf 0.01 --make-bed --out file_qcsample_snp

plink --bfile all_case_1 --extract all_case1_rs.txt --ci 0.95 --covar case_covar.txt --hide-covar --linear --out all_case_age_onset

plink --bfile under+control --ci 0.95 --covar under50_control_covar.txt --hide-covar --logistic --out under50+control_lo

LD:

plink --bfile all_dataset --extract rs.txt --show-tags rs.txt --list-all

Frq:

plink --bfile all_dataset --extract rs.txt --freq --keep under_people.txt --out case_frq

## polygenic risk score

Rscript PRSice.R --dir . --prsice ./PRSice_linux --base e_g_PRS.txt --target ukb_under_cc --pheno ukb_people_pheno.txt --stat OR --binary-target T

PRSice 2.3.3 (2020-08-05)

https://github.com/choishingwan/PRSice

(C) 2016-2020 Shing Wan (Sam) Choi and Paul F. O'Reilly

GNU General Public License v3

If you use PRSice in any published work, please cite:

Choi SW, O'Reilly PF.

PRSice-2: Polygenic Risk Score Software for Biobank-Scale Data.

GigaScience 8, no. 7 (July 1, 2019)

./PRSice_linux \

--a1 A1 \

--bar-levels 0.001,0.05,0.1,0.2,0.3,0.4,0.5,1 \

--base e_g_PRS.txt \

--binary-target T \

--bp BP \

--clump-kb 250kb \

--clump-p 1.000000 \

--clump-r2 0.100000 \

--interval 5e-05 \

--lower 5e-08 \

--num-auto 22 \

--or \

--out PRSice \

--pheno ukb_people_pheno.txt \

--pvalue P \

--seed 3400813836 \

--snp SNP \

--stat OR \

--target ukb_under_cc \

--thread 1 \

--upper 0.5

## 10-fold cross validation

data <- read.table("1.txt",header = T)

set.seed(36)

require(caret)

folds <- createFolds(y=data[,1],k=10)

library(pROC)

max=0

num=0

auc_value<-as.numeric()

for(i in 1:10){

fold_test <- data[folds[[i]],]

fold_train <- data[-folds[[i]],]

fold_pre <- glm(Y~.,family=binomial(link='logit'),data=fold_train)

fold_predict <- predict(fold_pre,type='response',newdata=fold_test)

auc_value<- append(auc_value,as.numeric(auc(as.numeric(fold_test[,1]),fold_predict)))

}

num<-which.max(auc_value)

print(auc_value)

fold_test <- data[folds[[num]],]

fold_train <- data[-folds[[num]],]

fold_pre <- glm(Y~.,family=binomial(link='logit'),data=fold_train)

fold_predict <- predict(fold_pre,type='response',newdata=fold_test)

roc_curve <- roc(as.numeric(fold_test[,1]),fold_predict)

plot(smooth(roc_curve),type = "s",xlim=c(1,0),ylim=c(0,1),col="red",print.thres=TRUE,print.auc= TRUE,

main="ROC curve for the set with the largest AUC value")

## matchit

CRC<-read.delim("1.txt",header = T,check.names=F)

rownames(CRC)= CRC[,1]

CRC= CRC[,-1]

head(CRC)

library(MatchIt)

CRC_1 = matchit (case~sex+age+recruitment.center,data=CRC, method ="nearest", ratio =14)

summary(CRC_1)

mchdata1 <- match.data(CRC_1)

write.csv(mchdata1 , file = "data.match.3.csv",quote=F)

# pathway

a<-read.delim("pathway.txt",header = T)

library(ggplot2)

kegg_point=ggplot(a,aes(x=Pathway.Name,y=Gene.Ratio))+

geom_point(aes(color=-log10(P.value),size=Count),alpha=0.8)+

coord_flip()+

scale_color_gradient(low = "green",high = "red")+

theme(title=element_text(size = 10))+

theme(legend.margin=unit(0.3,"inches"),

legend.key.width=unit(0.3,"inches"),

legend.key.height=unit(0.3,"inches"),)+

theme(plot.title=element_text(size=rel(2),hjust=0.5),

axis.title=element_text(size=rel(1.5)),

axis.text=element_text(size=rel(1.5)))

kegg_point=kegg_point+scale_size_continuous(range=c(4,10))

kegg_point

#PCA

data <- read.table("PCA-age1.txt",header = T,row.names = 1,sep="\t",check.names = F)

head(data)

library(scatterplot3d)

colors.lib <- c("#CC0000","#1d419b")

colors <- colors.lib[as.numeric(data$TYPE)]

shapes.lib =c(1,16)

shapes <- shapes.lib[as.numeric(data$TYPE)]

scatterplot3d(data[,c("PC1","PC2","PC3")], pch = shapes, color = colors, angle=45,

main="PCA analysis",xlab="PCA1",ylab="PCA2",zlab="PCA3",cex.symbols= 1)

legend("bottom", c("Case","Control"),

col = colors.lib,pch = shapes.lib ,inset = -0.25,horiz = TRUE,xpd = TRUE)

##manhattan

library(qqman)

a<-read.delim("all_case_age.assoc.linear", sep="", header=T,check.names=F)

head(a)

manhattan(a,chr="CHR", bp="BP", snp="SNP",genomewideline = F,suggestiveline = F, p="P", main = "Stage age", ylim = c(1, 15),col = color_set,annotatePval = 0.0005)

##Q-Q plot

library(qqman)

a<-read.delim("all_case_age.assoc.linear", sep="", header=T,check.names=F)

qq(a$P, main = "Q-Q plot of age p-values", xlim = c(0, 7), ylim = c(0,12), pch = 18, col = "blue4", cex = 1.5, las = 1)

p_value=a$P

z = qnorm(p_value/ 2)

lambda = round(median(z^2, na.rm = TRUE) / 0.454, 3)

#Spearman #

library("corrplot")

a<-read.delim("1.txt", sep="", header=T,check.names=F)

head(a)

corr<-cor(a,method="spearman")

cor.test(a$HEMASG,a$OHGSG,alternative="two.side",method="spearman")

corrplot(corr)

corrplot(corr,type="upper", addCoef.col="black", diag=F, method="pie",

order="hclust",tl.col="black", tl.srt=45)
